# Structure–Activity Relationship Studies on Highly Functionalized Pyrazole Hydrazones and Amides as Antiproliferative and Antioxidant Agents

**DOI:** 10.3390/ijms25094607

**Published:** 2024-04-23

**Authors:** Matteo Lusardi, Maria Grazia Signorello, Eleonora Russo, Debora Caviglia, Marco Ponassi, Erika Iervasi, Camillo Rosano, Chiara Brullo, Andrea Spallarossa

**Affiliations:** 1 Department of Pharmacy, University of Genova, Viale Benedetto XV, 3, 16132 Genova, Italy; matteo.lusardi@edu.unige.it (M.L.); mariagrazia.signorello@unige.it (M.G.S.); eleonora.russo@unige.it (E.R.); debora.caviglia@edu.unige.it (D.C.); chiara.brullo@unige.it (C.B.); 2 Proteomics and Mass Spectrometry Unit, IRCCS Ospedale Policlinico San Martino, Largo R. Benzi 10, 16132 Genova, Italy; marco.ponassi@hsanmartino.it (M.P.); erika.iervasi@hsanmartino.it (E.I.); camillo.rosano@hsanmartino.it (C.R.)

**Keywords:** pyrazole synthesis, antiproliferative agents, antioxidant activity, ROS production inhibition, platelet aggregation

## Abstract

Aminopyrazoles represent interesting structures in medicinal chemistry, and several derivatives showed biological activity in different therapeutic areas. Previously reported 5-aminopyrazolyl acylhydrazones and amides showed relevant antioxidant and anti-inflammatory activities. To further extend the structure–activity relationships in this class of derivatives, a novel series of pyrazolyl acylhydrazones and amides was designed and prepared through a divergent approach. The novel compounds shared the phenylamino pyrazole nucleus that was differently decorated at positions 1, 3, and 4. The antiproliferative, antiaggregating, and antioxidant properties of the obtained derivatives **10**–**22** were evaluated in in vitro assays. Derivative **11a** showed relevant antitumor properties against selected tumor cell lines (namely, HeLa, MCF7, SKOV3, and SKMEL28) with micromolar IC_50_ values. In the platelet assay, selected pyrazoles showed higher antioxidant and ROS formation inhibition activity than the reference drugs acetylsalicylic acid and *N*-acetylcysteine. Furthermore, in vitro radical scavenging screening confirmed the good antioxidant properties of acylhydrazone molecules. Overall, the collected data allowed us to extend the structure–activity relationships of the previously reported compounds and confirmed the pharmaceutical attractiveness of this class of aminopyrazole derivatives.

## 1. Introduction

**Figure 1:**
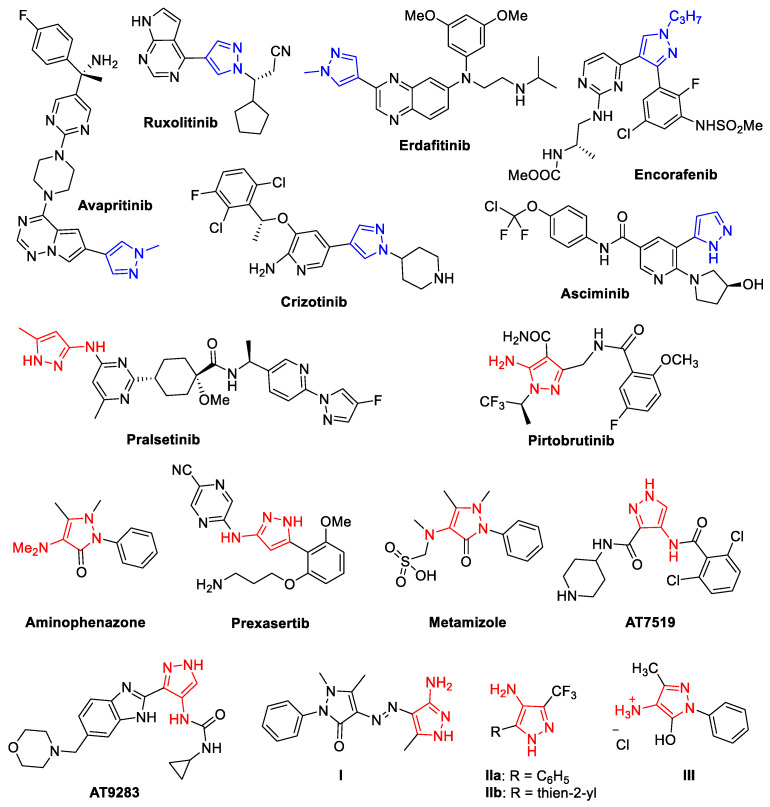
Selected examples of pyrazole compounds with relevant pharamacological activity. The pyrazole and aminopyrazole substructures are colored blue and red, respectively.

Pyrazole scaffold is a pharmaceutically relevant moiety [1,2,3,4,5,6], and pyrazole-containing compounds show antiviral [7], antibacterial [8,9], antimalarial [10], anti-inflammatory [11], antidiabetic [12], antiglaucoma [13,14], and anticancer [15,16,17,18,19,20,21] properties. Furthermore, pyrazole scaffolds are shared by several protein kinase inhibitors, including FDA-approved drugs Avapritinib, Asciminib, Crizotinib, Encorafenib, Erdafitinib, Pralsetinib, Pirtobrutinib, and Ruxolitinib (Figure 1) [22,23]. Among pyrazole series, aminopyrazoles (APs) represent an attractive framework in medicinal chemistry [24,25,26]; indeed, the decoration of the pyrazole ring with amino substituents at different positions led to the isolation of pharmacologically active derivatives including analgesic (e.g., Aminophenazone and Metamizole; Figure 1) and antitumor (e.g., AT7519, AT9283, Prexasertib, Pirtobrutinib/Jaypirca™; Figure 1) agents [23,27,28,29,30,31,32,33]. Additionally, the AP scaffold has been widely studied for its relevant activity in oxidative stress and inflammation. In detail, 3-AP **I** (Figure 1) showed weak antiproliferative activity against four tumor cell lines (i.e., HepG2, WI38, VERO, and MCF-7), but exhibited high antioxidant activity, due to the free amino group on the pyrazole ring [34]. 4-APs **IIa**,**b** (Figure 1) and their hydrochloride salts displayed excellent antiradical activity in the ABTS scavenging assay, with Trolox equivalent antioxidant capacity (TEAC) values of 1.35 and 1.10 and IC_50_ of 14.1 µM and 17.6 µM, respectively. Additionally, the two compounds confirmed their promising antioxidant properties in the oxygen radical absorbance capacity assay (ORAC) and in the oxidative erythrocyte hemolysis assay [35]. Structure–activity relationships (SARs) extension on **IIa**,**b** led to the isolation of pyrazole hydrochloride **III** (Figure 1) endowed with improved pharmacokinetic and antioxidant properties. Further statistical analysis and cytotoxicity studies confirmed the promising profile of the compound, which was taken as the lead structure for the development of effective agents against oxidative stress-related diseases [36].

**Figure 2:**
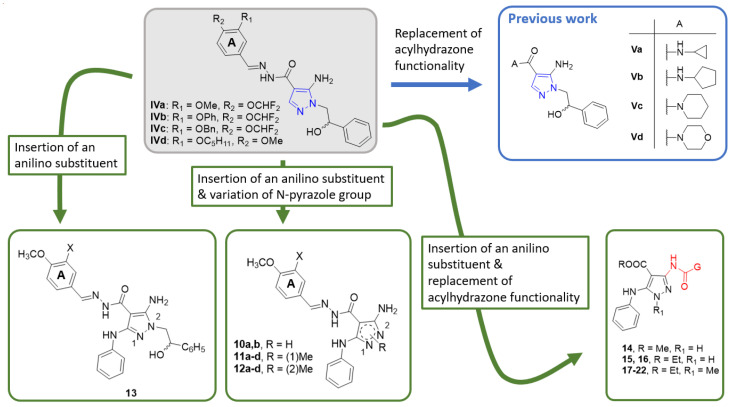
Developed SARs around pyrazolyl hydrazones **IV**.

More recently, 5-AP acylhydrazones **Iva**–**d** (Figure 2) proved to inhibit platelet aggregation and reactive oxygen species (ROS) production with IC_50_ values in the low micromolar range [37]. In particular, derivative **IVd** showed antioxidant and anti-inflammatory dual activity, inhibiting ROS production in fMLP-activated neutrophils and blocking PDE4B and PDE4D phosphodiesterase enzymes (IC_50_ = 1.05 µM and 0.55 µM, respectively), two PDE4 isoforms involved in inflammatory processes [37]. Furthermore, pyrazoles **IVa**–**c** strongly reduced superoxide anion production, lipid peroxidation, and NAPDH oxidase activity in H_2_O_2_-stimulated EA.hy926 cells, thus highlighting the potential of compounds on oxidative status and aerobic metabolism [38]. In previous work, the SARs of hydrazones **IV** were further extended through the preparation of amide derivatives **V** (Figure 2), able to inhibit both aggregation and ROS formation in platelets and p38MAPK phosphorylation [39].

To further exploit the pharmacological potentials of derivatives **IV** and **V** (Figure 2), a novel series of pyrazoles **10**–**22** have been studied for their antiproliferative and antioxidant activities. In particular, acylhydrazones **10**–**13** bear an anilino substituent on the pyrazole scaffold (not present in the structures of the lead derivatives **IV** and **V**) with or without concomitant variation of the pyrazole 2-hydroxy-2-phenylethyl chain (derivative **13**: no modification; derivatives **10**: removal of the chain; derivatives **11** and **12**: replacement of the chain with a methyl substituent). The substituents of ring A were selected according to the SARs developed for compounds **IV** (X = H, OMe, OBn, OPh). Additionally, to evaluate the effects on activity of the acylhydrazone moiety, pyrazolyl amides **14**–**22** were prepared. These compounds share with their acylhydrazone congeners the anilino substituent and bear, as G groups, cyclopropyl, or differently substituted phenyl rings (namely, 4-Cl, 4-OMe, 2,6-(OMe)_2_; 3,4-(OMe)_2_; 3,4,5-(OMe)_3_), characterizing the most effective derivatives **IV** or **V**.

## 2. Results

### 2.1. Chemistry

**Scheme 1:**
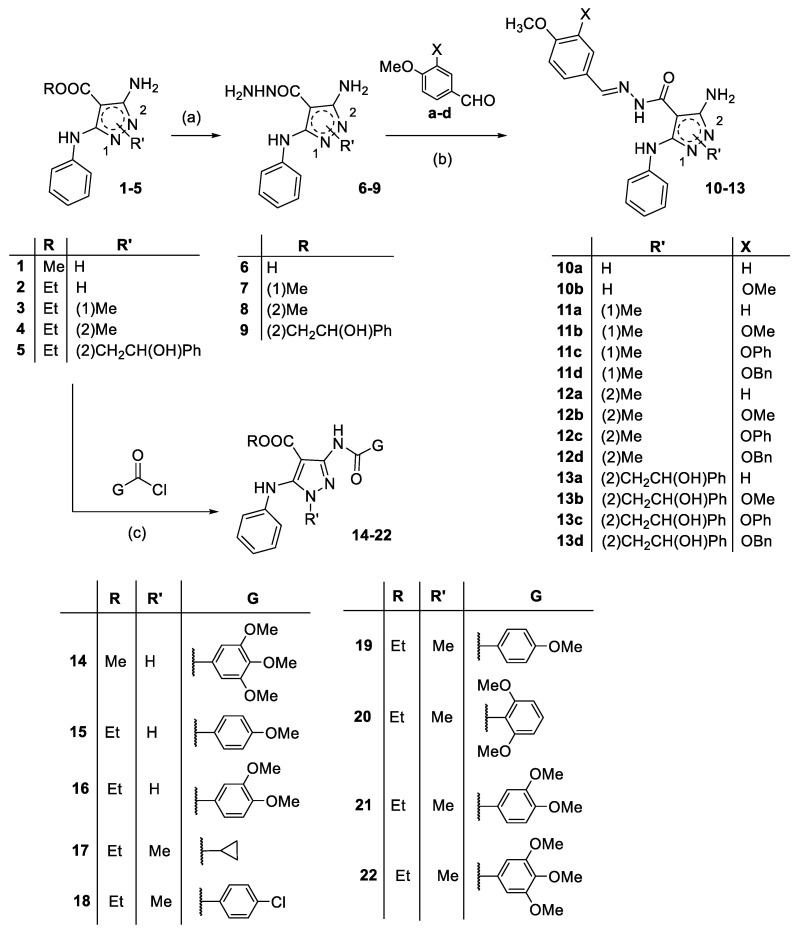
Synthesis of pyrazole acylhydrazones **10**–**13** and pyrazolyl amides **14**–**22**. Reaction conditions: (a) hydrazine monohydrate, EtOH_abs_, reflux, 4–6 h; (b) aldehyde **a**–**d**, EtOH_abs_, reflux, 16 h; (c) acyl chloride, TEA or TMEDA, DMF or ACN or DCM, various temperatures and times.

The desired compounds **10**–**22** were obtained through a divergent, stepwise approach, starting from the common AP intermediates **1**–**5** (Scheme 1). These key building blocks were prepared through the condensation of cyanoacetic ester, phenyl isothiocyanate, and (un)substituted hydrazine, as previously reported [40,41,42]. Thus, synthons **2**–**5** were condensed with hydrazine monohydrate, leading to the corresponding carbohydrazide intermediates **6**–**9** in good yields (Scheme 1); these derivatives were then reacted with 4-methoxy benzaldehydes **a–d** (commercially available or prepared by alkylation or arylation of isovanillin according to the published procedures) [43,44] in absolute ethanol to afford the desired compounds **10**–**13** in moderate to good yields (13–80%, Scheme 1). Interestingly, this reaction proved to be stereoselective, allowing the unique isolation of the E-isomer, as assessed by proton NMR analysis. In fact, acylhydrazones **10**–**13** showed the signal of the acylhydrazone proton at chemical shift values lower than 12 ppm (chemical shift range: 9.59–10.65 ppm), typical of the E-isomer as recently reported for similar hydrazones [37].

Amides **14**–**22** were prepared through the condensation of APs **1**–**3** with the proper acyl chloride (namely, cyclopropyl carbonyl chloride, 4-chlorobenzoyl chloride, and differently methoxy-substituted benzoyl chlorides; Scheme 1), selected according to the SAR developed for the acylhydrazone series. The different reactivity of the acyl chlorides towards the pyrazole amino group required the definition of different experimental protocols. Thus, for derivatives **14**–**16** and **20**–**22**, the reaction was carried out at rt in dichloromethane (DCM) with the addition of triethylamine (TEA), while pyrazoles **17** and **18** were prepared in anhydrous *N*,*N*-dimethylformamide (DMF) at 120 °C in the presence of *N*,*N*,*N’*,*N’*-tetramethylethylenediamine (TMEDA). Interestingly, TMEDA plays a dual role in the acylation reaction, acting as a HCl scavenger and further activating the acyl chloride through the formation of a pseudo-cyclic complex, as previously described in the literature [45,46]. Finally, compound **19** was prepared at rt in acetonitrile (ACN) using TEA as a base. The acylation of pyrazole **4** (regioisomer of **3**) was tried in different experimental conditions. However, the amino group proved to be unreactive, possibly due to the steric hindrance of the proximal methyl group.

### 2.2. Antiproliferative Properties

Pyrazole acylhydrazones **10**–**13** and amides **14**–**22** were tested by MTT assay to evaluate their antiproliferative and cytotoxicity properties against a panel of eight tumor cell lines and normal fibroblasts. The compounds were screened at a fixed concentration of 10 µM, and cisplatin (10 µM) was used as a reference drug. As reported in Table 1, the majority of acylhydrazones did not show any cytotoxic activity against tumor and fibroblast cells, displaying mean growth percentage values higher than 50%. However, AP **11a** significantly inhibits the proliferation of HeLa (25.00%, Table 1), MCF7 (33.56%, Table 1), SKOV3 (43.60%, Table 1), and SKMEL28 (49.44%, Table 1) cancer cells, resulting in more efficacy than cisplatin against HeLa and MCF7 cells. The antiproliferative activity of the compound marginally affected the growth of normal fibroblasts, resulting in less cytotoxicity than the reference cisplatin (69.81% vs. 39.52% mean growth percentages, respectively). 

The pyrazolyl amides **14**–**22** showed poor antiproliferative activity against all tested tumor cell lines, with exceptions made for derivatives **14** (active against SKMEL28, SKOV3, A549, and HeLa cells) and **16** (active against Hep-G2). In particular, **14** was found to be more effective than cisplatin against HeLa, A549, and SKMEL28 cells. Differently from its amide analogues, N-unsubstituted 3,4,5-trimethoxybenzoyl pyrazole **14** was as cytotoxic as cisplatin against normal GM6114 fibroblasts (mean growth percentage = 39.31%).

The remarkable antiproliferative, non-cytotoxic activity of **11a** was further investigated, and the IC_50_ values against HeLa, MCF7, SKOV3, and SKMEL28 cell lines were determined. The compound showed cell proliferation inhibition values in the micromolar concentration range (IC_50_(HeLa) = 4.63 ± 0.41 µM; IC_50_(MCF7) = 6.90 ± 0.34 µM; IC_50_(SKOV3) = 6.88 ± 0.23 µM; IC_50_(SKMEL28) = 9.45 ± 0.66 µM), confirming its promising antiproliferative profile.

In addition, **11a** and **17** (representative compounds of the acylhydrazone and amide series) were selected by the National Cancer Institute (NCI, Germantown, MD, USA) and tested at a fixed concentration of 10 µM against a panel of fifty-nine different cancer cell lines, including highly metastatic tumors (Table 2). Pyrazolyl amide **17** did not show any antiproliferative activity (growth percentage range = 81.47–118.49%), whereas **11a** confirmed its promising antitumor properties, showing growth percentage values lower than 20% against leukemia (HL-60(TB), K-562, SR cells), NSCLC (NCI-H460 and NCI-H522 cells), colon (HCT-116, HCT-15, HT29, and SW-620 cells), breast (MCF7, HS 578T, MDA-MB-468 cells), and melanoma (LOX IMVI, M14, and MDA-MB-435 cells).

### 2.3. Antioxidant Evaluation

**Figure 3:**
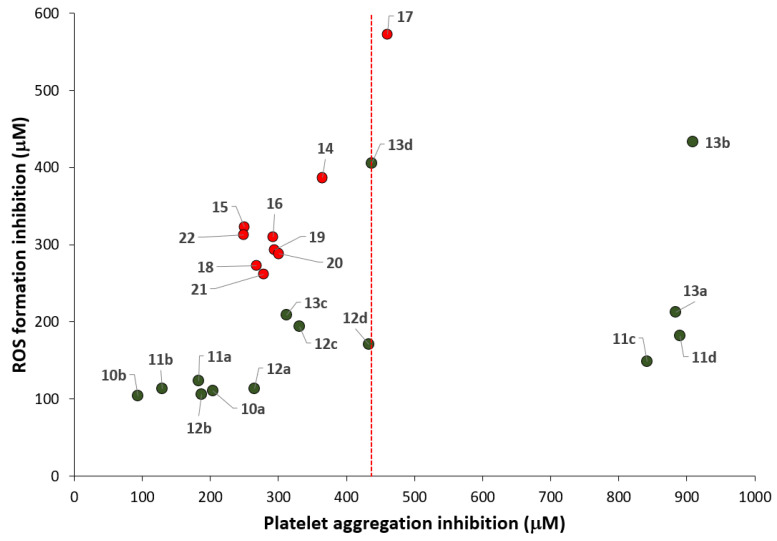
Bidimensional plot of ROS formation inhibition and antiaggregant activity of derivatives **10**–**22**. Pyrazolyl amides are colored red, and acylhydrazones are reported as green dots. The dashed red line indicates the antiaggregant IC_50_ value of the reference drug ASA (IC_50_ = 438 µM). All compounds were found to be more effective ROS formation inhibitors than NAC (IC_50_ = 872 µM).

The antioxidant properties of acylhydrazones **10**–**13** and pyrazolyl amides **14**–**22** were tested by evaluating their inhibition of platelet aggregation and ROS production (Figure 3, Table S1). In fact, human platelets could represent a fast and low-cost biological model to screen compounds as anticancer, anti-inflammatory, and antiaggregating agents [47,48,49]. Moreover, ROS production inhibition, related to human platelet aggregation, could provide a good indication of the anti-inflammatory and antioxidant properties of the newly synthesized compounds [47,50,51,52]. *N*-acetylcysteine (NAC) and acetyl salicylic acid (ASA) were used as reference drugs for antioxidant and antiaggregant activities, respectively. 

All derivatives blocked ROS production more effectively than NAC, and most of the tested compounds (18 out of 23) showed improved antiaggregant properties in comparison with ASA. 

### 2.4. DPPH Radical-Scavenging Activity

The antioxidant activity of the representative compounds of the two series (namely, acylhydrazones **10b**, **11a**, **11d**, **12d**, **13d**, and amides **14**, **22**) was measured in vitro using the 2,2-diphenyl-1-picrylhydrazyl (DPPH) assay [53]. The results were calculated as Trolox equivalent and expressed as a percentage of antioxidant activity (AA) (Table 3). Amides **14** and **22** showed poor antioxidant properties, whereas hydrazone compounds displayed significant AA values (range 15.53–76.45%). Derivatives **10b** and **12d** were endowed with the highest antioxidant activity, thus highlighting the relevance of both pyrazole N1 and acylhydrazone substituents on compounds’ radical scavenging properties.

## 3. Discussion

To further extend the SARs of lead derivatives **IV** and **V**, acylhydrazones **10**–**13** and amides **14**–**22** were prepared through a divergent, regioselective synthetic protocol. The novel acylhydrazone derivatives showed limited antiproliferative activity in cell-based assays, with an exception made for derivative **11a** that significantly inhibits the duplication of leukemia, non-small cell lung cancer (NSCLC), colon cancer, CNS cancer, melanoma, and breast cancer cells, showing the highest inhibitory activity against the cell line (Table 1 and Table 2). Noteworthy, derivative **11a** showed a lethal effect against melanoma MDA-MB-435 and breast cancer MDA-MB-468 cell lines, thus confirming the attractiveness of this molecule as a lead structure for the development of novel anticancer agents. Among amides, the N-unsubstituted compound **14** was more effective than cisplatin against cervical HeLa and lung A549 cancer cells, also affecting the proliferation of ovarian SKOV3. Unfortunately, the observed antiproliferative effects were coupled with significant cytotoxicity against normal fibroblasts. 

Derivatives **10**–**22** showed a reduced antioxidant activity in comparison with lead compounds **IV** and **V** [37,39], still resulting in more effective than reference NAC (IC_50_ = 872 µM) in inhibiting ROS production. Moreover, the majority of the compounds showed increased anti-platelet aggregation properties in comparison to ASA (IC_50_ = 438 µM). The ability of acyl hydrazone compounds **10**–**13** to inhibit ROS formation and platelet aggregation appears to be affected by the substitution of both the pyrazole nucleus and the phenyl carbohydrazide ring. Thus, unsubstituted pyrazoles **10** and *N*-methyl pyrazoles **11** and **12** proved to be more active than their sterically hindered congeners **13**. Moreover, compounds bearing a 4-methoxyphenyl or a 3,4-dimethoxyphenyl substituent (i.e., derivatives **10a**,**b**, **11a**,**b**, and **12a**,**b**) were endowed with the lower IC_50_ values for both platelet aggregation (94–265 µM, Table S1) and ROS production (104–123 µM, Table S1) inhibition. Within the pyrazolyl amide series, the aromatic nature of the amide substituent emerged to be critical for activity. Thus, benzamido analogues **14**–**16**, **18**–**22** showed anti-ROS and antiaggregant effects in a narrow IC_50_ range (ROS production IC_50_ range = 262–387 µM; antiaggregant IC_50_ range = 249–365 µM), resulting in greater effectiveness than the reference drugs. Conversely, the cyclopropyl amino analogue **17** was found to be less effective than its congeners (IC_50_ (ROS) = 573 µM; IC_50_ (aggregation) = 460 µM), with reduced antiaggregant properties in comparison with ASA.

In the DPPH radical-scavenging assay, amides **14** and **22** were less effective than their hydrazone analogues (Table 3), thus highlighting the relevance of this moiety for activity (Table 3). Among tested derivatives, pyrazole hydrazones **10b**, **12d**, and **13d** proved to be more effective than their analogues **11a**,**d**, indicating that the insertion of a methyl substituent on the pyrazole *N*-atom adjacent to the phenylamino group was detrimental for activity. Noteworthy, these data suggest that the antiproliferative activity of the prepared series (and, in particular, that of derivatives **11a** and **14**) does not correlate with the compounds’ in vitro anti-scavenging properties.

Conversely, the high radical scavenging properties of **10b** (AA% = 76.45%) well correlate with the compound’s antiaggregant and ROS inhibitory activities observed in platelets.

**Figure 4:**
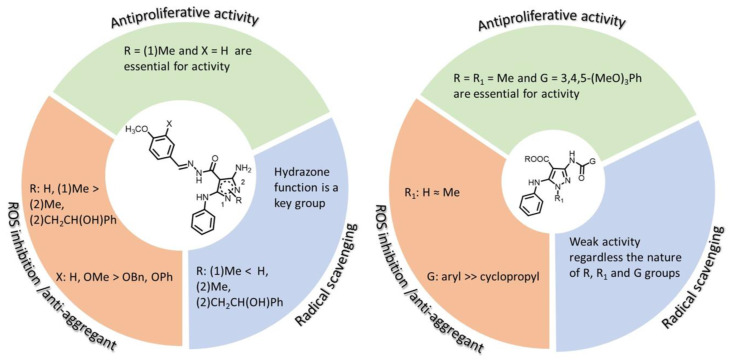
SARs developed for acylhydrazones **10**–**13** and pyrazolyl amides **14**–**22**.

The developed SARs for the two series of compounds are summarized in Figure 4.

## 4. Materials and Methods

### 4.1. Chemistry

Reagents were purchased by Alfa-Aesar and Sigma-Aldrich. 3,4-dimethoxybenzaldehyde, 3-methoxy-4-phenoxybenzaldehyde, and 4-(benzyloxy)-3-methoxybenzaldehyde were prepared according to published procedures [43,44,54]. All the solvents were reagent grade and were dried on molecular sieves (5 Å 1/16” inch pellets). Unless otherwise stated, all commercial reagents were used without further purification. Organic solutions were dried over anhydrous sodium sulphate. Aluminium-backed silica gel thin layer chromatography (TLC) plates (Merck DC-Alufolien Kieselgel 60 F_254_) were used for reaction monitoring and purity analyses. A DCM/MeOH 9:1 mixture was used as a developing solvent, and spots were detected by UV light and/or by iodine vapors. Melting points were measured on a Fisher-Johns apparatus and are uncorrected. ^1^H NMR and ^13^C NMR spectra were collected on a JEOL JNM-ECZR (Tokyo, Japan) instrument (Figures S1–S44); chemical shifts were reported in δ (ppm) units, and the splitting patterns were described as follows: s (singlet), bs (broad singlet), d (doublet), t (triplet), q (quartet), and m (multiplet). The first-order values reported for coupling constants *J* were given in Hz.The elemental composition of synthesized compounds was collected by an EA1110. Pyrazoles **1**–**5** were synthesized as previously reported [40,41,42]. 

#### 4.1.1. General Synthesis of Intermediates **6**–**9**

A mixture of the proper pyrazole **2**–**5** (2 mmol) and hydrazine monohydrate (2 mL) was refluxed for 4–6 h. After cooling at rt, water (15 mL) was added, and the solution was acidified with HCl 2 M. The precipitate was collected by filtration and used without further purification. For compound **6**, the excess of hydrazine was removed under reduced pressure, and the crude mixture was purified by column chromatography (silica gel, eluent: Et_2_O-Et_2_O/20% EtOH).

*3-Amino-5-(phenylamino)-1H-pyrazole-4-carbohydrazide* **6**

Colourless oil. Yield 55%. Calcd for C_10_H_12_N_6_O: C = 51.72; H = 5.21; N = 36.19. Found: C = 36.07; H = 5.28. N = 5.18.

*3-Amino-1-methyl-5-(phenylamino)-1H-pyrazole-4-carbohydrazide* **7**

Mp 226–228 °C (H_2_O); yield 83%. Calcd for C_11_H_14_N_6_O: C = 53.65; H = 5.73; N = 34.13. Found: C = 53.34; H = 5.74; N = 34.07.

*5-Amino-1-methyl-3-(phenylamino)-1H-pyrazole-4-carbohydrazide* **8**

Mp 200–204 °C (H_2_O); yield: 71%. Calcd for C_11_H_14_N_6_O: C = 53.65; H = 5.73; N = 34.13. Found: C = 53.88; H = 5.69; N = 34.21.

*5-Amino-1-(2-hydroxy-2-phenylethyl)-3-(phenylamino)-1H-pyrazole-4-carbohydrazide* **9**

Mp 177–180 °C (H_2_O); yield 53%. Calcd for C_18_H_20_N_6_O_2_: C = 61.35; H = 5.72; N = 23.85. Found: C = 61.40; H = 5.67; N = 24.03.

#### 4.1.2. General Synthetic Procedure for the Preparation of Pyrazole Acylhydrazones **10**–**13**

To a solution of the proper intermediate **6**–**9** (1 mmol) in absolute EtOH (5 mL), the suitable benzaldehyde **a**–**d** (1 mmol) was added. The reaction mixture was stirred at reflux for 16 h and then cooled at rt. The precipitate was collected by filtration and crystallized with ethanol.

*(E)-3-amino-N′-(4-methoxybenzylidene)-5-(phenylamino)-1H-pyrazole-4-carbohydrazide* **10a**.

Mp 227–232 °C (EtOH); Yield 24%. ^1^H NMR (400 MHz, DMSO-d_6_): δ 3.79 (s, 3H, OCH_3_); 6.15 (bs, 2H, NH_2_, exchangeable); 6.73–6.79 (m, 1H, arom. H); 6.97–7.02 (m, 2H, arom. H); 7.16–7.22 (m, 2H, arom. H); 7.35–7.41 (m, 2H, arom. H); 7.58–7.61 (m, 2H, arom. H); 8.13 (s, 1H, CH=C); 8.91 (bs, 1H, NH phenyl, exchangeable); 10.48 (bs, 1H, NH hydraz., exchangeable); 11.18 (bs, 1H, NH pyraz., exchangeable). ^13^C NMR (101 MHz, DMSO-d_6_): δ 55.32; 85.33; 114.45; 116.01; 118.96; 126.89; 128.37; 128.79; 142.53; 144.64; 148.48; 151.09; 160.62. Calcd for C_18_H_18_N_6_O_2_: C = 61.70; H = 5.18; N = 23.99. Found: C = 61.65; H = 5.11; N = 23.92.

*(E)-3-amino-N′-(3,4-dimethoxybenzylidene)-5-(phenylamino)-1H-pyrazole-4-carbohydrazide* **10b**.

Mp 263–266 °C (EtOH); Yield 13%. ^1^H NMR (400 MHz, DMSO-d_6_): δ 3.75 (s, 3H, OCH_3_); 3.79 (s, 3H, OCH_3_); 6.06 (bs, 2H, NH_2_, exchangeable); 6.73–6.80 (m, 1H, arom. H); 7.01–7.28 (m, 5H, arom. H); 7.44–7.49 (m, 2H, arom. H); 8.22 (s, 1H, CH=C); 9.24 (bs, 1H, NH phenyl, exchangeable); 10.65 (bs, 1H, NH hydraz., exchangeable); 11.37 (bs, 1H, NH pyraz., exchangeable). ^13^C NMR (101 MHz, DMSO-d_6_): δ 55.29; 55.58; 83.60; 115.44; 115.90; 116.02; 118.79; 118.98; 121.45; 128.79; 128.91; 141.91; 146.17; 151.26; 152.14; 171.47. Calcd for C_19_H_20_N_6_O_3_: C = 59.99; H = 5.30; N = 22.90. Found: C = 59.82; H = 5.33; N = 22.75.

*(E)-3-amino-N′-(4-methoxybenzylidene)-1-methyl-5-(phenylamino)-1H-pyrazole-4-carbohydrazide* **11a**.

Mp 208–210 °C (EtOH); Yield 51%. ^1^H NMR (400 MHz, DMSO-d_6_): δ 3.38 (s, 3H, NCH_3_); 3.78 (s, 3H, OCH_3_); 5.47 (bs, 2H, NH_2_, exchangeable); 6.58–6.63 (m, 2H, arom. H); 6.75–6.82 (m, 1H, arom. H); 6.93–7.00 (m, 2H, arom. H); 7.15–7.24 (m, 2H, arom. H); 7.53–7.59 (m, 2H arom. H); 7.91 (s, 1H, CH=C); 8.09 (bs, 1H, NH phenyl, exchangeable); 10.14 (bs, 1H, NH hydraz., exchangeable). ^13^C NMR (101 MHz, DMSO-d_6_): δ 34.60; 55.30; 93.60; 114.35; 119.80; 126.74; 128.45; 129.54; 138.04; 144.49; 145.17; 155.32; 160.66. Calcd for C_19_H_20_N_6_O_2_: C = 62.62; H = 5.53; N = 23.06. Found: C = 62.59; H = 5.51; N = 22.94.

*(E)-3-amino-N′-(3,4-dimethoxybenzylidene)-1-methyl-5-(phenylamino)-1H-pyrazole-4-carbohydrazide* **11b**.

Mp 218–220 °C (EtOH); Yield 60%. ^1^H NMR (400 MHz, DMSO-d_6_): δ 3.38 (s, 3H, NCH_3_); 3.77 (s, 3H, OCH_3_); 3.78 (s, 3H, OCH_3_); 5.47 (bs, 2H, NH_2_, exchangeable); 6.60–6.64 (m, 2H, arom. H); 6.77–6.82 (m, 1H, arom. H); 6.96–7.00 (m, 1H, arom. H); 7.10–7.14 (m, 1H, arom. H); 7.18–7.25 (m, 3H arom. H); 7.91 (s, 1H, CH=C); 8.09 (bs, 1H, NH phenyl, exchangeable); 10.16 (bs, 1H, NH hydraz., exchangeable). ^13^C NMR (101 MHz, DMSO-d_6_): δ 34.58; 55.38; 55.55; 93.61; 108.18; 109.03; 111.49; 114.32; 119.81; 121.37; 123.56; 126.89; 129.54; 144.49; 145.47; 149.01; 150.51; 151.62; 155.30; 160.85. Calcd for C_20_H_22_N_6_O_3_: C = 60.90; H = 5.62; N = 21.31. Found: C = 61.40; H = 5.54; N = 21.05.

*(E)-3-amino-N′-(4-methoxy-3-phenoxybenzylidene)-1-methyl-5-(phenylamino)-1H-pyrazole-4-carbohydrazide* **11c**.

Mp 226–228 °C (EtOH); Yield 49%. ^1^H NMR (400 MHz, DMSO-d_6_): δ 3.36 (s, 3H, NCH_3_); 3.79 (s, 3H, OCH_3_); 5.44 (bs, 2H, NH_2_, exchangeable); 6.57–6.62 (m, 2H, arom. H); 6.76–6.81 (m, 1H, arom. H); 6.87–6.90 (m, 2H, arom. H); 7.04–7.08 (m, 1H, arom. H); 7.16–7.22 (m, 3H arom. H); 7.26–7.35 (m, 3H, arom. H); 7.40–7.44 (m, 1H, arom. H); 7.90 (s, 1H, CH=C); 8.05 (bs, 1H, NH phenyl, exchangeable); 10.19 (bs, 1H, NH hydraz., exchangeable). ^13^C NMR (101 MHz, DMSO-d_6_): δ 34.59; 55.87; 93.55; 113.35; 114.33; 116.80; 118.03; 119.80; 122.75; 124.88; 127.44; 129.53; 129.93; 138.16; 144.41; 152.56; 155.31; 157.29; 159.90. Calcd for C_25_H_24_N_6_O_3_: C = 65.78; H = 5.30; N = 18.41. Found: C = 65.55; H = 5.28; N = 18.21.

*(E)-3-amino-N′-(3-(benzyloxy)-4-methoxybenzylidene)-1-methyl-5-(phenylamino)-1H-pyrazole-4-carbohydrazide* **11d**.

Mp 183–186 °C (EtOH); Yield 80%. ^1^H NMR (400 MHz, DMSO-d_6_): δ 3.34 (s, 3H, NCH_3_); 3.76 (s, 3H, OCH_3_); 5.05 (s, 2H, CH_2_Ph); 5.44 (bs, 2H, NH_2_, exchangeable); 6.55–6.61 (m, 2H, arom. H); 6.73–6.78 (m, 1H, arom. H); 6.96–7.00 (m, 1H, arom. H); 7.12–7.19 (m, 2H, arom. H); 7.28–7.44 (m, 7H arom. H); 7.86 (s, 1H, CH=C); 8.04 (bs, 1H, NH phenyl, exchangeable); 10.11 (bs, 1H, NH hydraz., exchangeable). ^13^C NMR (101 MHz, DMSO-d_6_): δ 34.60; 55.65; 69.83; 93.65; 110.09; 111.82; 114.32; 119.81; 121.54; 126.86; 127.92; 128.01; 128.46; 129.55; 136.88; 144.49; 148.05; 150.79; 155.29; 160.78. Calcd for C_26_H_26_N_6_O_3_: C = 66.37; H = 5.57; N = 17.86. Found: C = 66.45; H = 5.48; N = 17.72.

*(E)-5-amino-N′-(4-methoxybenzylidene)-1-methyl-3-(phenylamino)-1H-pyrazole-4-carbohydrazide* **12a**.

Mp 195–198 °C (EtOH); Yield 50%. ^1^H NMR (400 MHz, DMSO-d_6_): δ 3.52 (s, 3H, NCH_3_); 3.79 (s, 3H, OCH_3_); 6.39 (bs, 2H, NH_2_, exchangeable); 6.74–6.79 (m, 1H, arom. H); 6.98–7.02 (m, 2H, arom. H); 7.16–7.22 (m, 2H, arom. H); 7.35–7.39 (m, 2H, arom. H); 7.58–7.63 (m, 2H arom. H); 8.12 (s, 1H, CH=C); 8.90 (bs, 1H, NH phenyl, exchangeable); 10.52 (bs, 1H, NH hydraz., exchangeable). ^13^C NMR (101 MHz, DMSO-d_6_): δ 34.12; 55.32; 85.63; 114.45; 116.06; 119.06; 126.83; 128.36; 128.78; 130.03; 142.42; 144.58; 147.40; 149.62; 160.63. Calcd for C_19_H_20_N_6_O_2_: C = 62.62; H = 5.53; N = 23.06. Found: C = 62.56; H = 5.63; N = 22.99.

*(E)-5-amino-N′-(3,4-dimethoxybenzylidene)-1-methyl-3-(phenylamino)-1H-pyrazole-4-carbohydrazide* **12b**.

Mp 104–106 °C (EtOH); Yield 35%. ^1^H NMR (400 MHz, CDCl_3_): δ 3.59 (s, 3H, NCH_3_); 3.83 (s, 3H, OCH_3_); 3.92 (s, 3H, OCH_3_); 5.45 (bs, 2H, NH_2_, exchangeable); 6.82–6.85 (m, 1H, arom. H); 6.90–6.94 (m, 1H, arom. H); 7.01–7.05 (m, 1H, arom. H); 7.23–7.30 (m, 4H, arom. H); 7.32–7.36 (m, 2H, arom. H + CH=C); 7.72 (bs, 1H, NH phenyl, exchangeable); 9.80 (bs, 1H, NH hydraz., exchangeable). ^13^C NMR (101 MHz, CDCl_3_): δ 33.92; 55.94; 89.16; 108.12; 110.66; 116.70; 120.73; 122.31; 126.54; 129.34; 143.03; 145.57; 148.90; 149.41; 151.10; 163.16. Calcd for C_20_H_22_N_6_O_3_: C = 60.90; H = 5.62; N = 21.31. Found: C = 60.65; H = 5.38; N = 21.45.

*(E)-5-amino-N′-(4-methoxy-3-phenoxybenzylidene)-1-methyl-3-(phenylamino)-1H-pyrazole-4-carbohydrazide* **12c**.

Mp 234–236 °C (EtOH); Yield 68%. ^1^H NMR (400 MHz, DMSO-d_6_): δ 3.51 (s, 3H, NCH_3_); 3.80 (s, 3H, OCH_3_); 6.31 (bs, 2H, NH_2_, exchangeable); 6.74–6.78 (m, 1H, arom. H); 6.87–6.91 (m, 2H, arom. H); 7.04–7.09 (m, 1H, arom. H); 7.15–7.25 (m, 3H, arom. H); 7.30–7.37 (m, 5H arom. H); 7.44–7.48 (m, 1H, arom. H); 8.10 (s, 1H, CH=C); 8.79 (bs, 1H, NH phenyl, exchangeable); 10.50 (bs, 1H, NH hydraz., exchangeable). ^13^C NMR (101 MHz, DMSO-d_6_): δ 34.09; 55.89; 85.70; 113.45; 116.05; 116.70; 118.13; 119.08; 122.69; 124.82; 127.60; 128.77; 129.91; 142.45; 144.11; 144.40; 147.36; 149.47; 152.54; 157.33; 162.61. Calcd for C_25_H_24_N_6_O_3_: C = 65.78; H = 5.30. N = 18.41; Found: C = 65.70; H = 5.30; N = 18.35.

*(E)-5-amino-N′-(3-(benzyloxy)-4-methoxybenzylidene)-1-methyl-3-(phenylamino)-1H-pyrazole-4-carbohydrazide* **12d**.

Mp 131–133 °C (EtOH); Yield 91%. ^1^H NMR (400 MHz, DMSO-d_6_): δ 3.53 (s, 3H, NCH_3_); 3.81 (s, 3H, OCH_3_); 5.01 (s, 2H, CH_2_Ph); 6.41 (bs, 2H, NH_2_, exchangeable); 6.74–6.80 (m, 1H, arom. H); 7.01–7.06 (m, 1H, arom. H); 7.16–7.22 (m, 3H, arom. H); 7.35–7.44 (m, 8H, arom. H); 8.09 (s, 1H, CH=C); 8.90 (bs, 1H, NH phenyl, exchangeable); 10.59 (bs, 1H, NH hydraz., exchangeable). ^13^C NMR (101 MHz, DMSO-d_6_): δ 34.61; 56.16; 70.20; 86.14; 110.17; 112.37; 116.64; 119.63; 122.09; 127.42; 128.45; 128.93; 129.29; 137.32; 142.84; 145.11; 148.21; 148.65; 149.89; 151.25; 161.27. Calcd for C_26_H_26_N_6_O_3_: C = 66.37; H = 5.57; N = 17.86. Found: C = 66.34; H = 5.60. N = 17.68.

*(E)-5-amino-1-(2-hydroxy-2-phenylethyl)-N′-(4-methoxybenzylidene)-3-(phenylamino)-1H-pyrazole-4-carbohydrazide* **13a**.

Mp 120–122 °C (EtOH); Yield 52%. ^1^H NMR (400 MHz, CDCl_3_): δ 3.78 (s, 3H, OCH_3_); 3.85–4.04 (m, 3H, CH_2_N + CHOH); 5.12–5.17 (m, 1H, OH, exchangeable); 5.43 (bs, 2H, NH_2_, exchangeable); 6.76–6.81 (m, 2H, arom. H); 6.83–6.89 (m, 1H, arom. H); 7.14–7.23 (m, 4H, arom. H); 7.27–7.39 (m, 5H, arom. H); 7.45–7.50 (m, 2H, arom. H); 7.55 (s, 1H, CH=C); 8.61 (bs, 1H, NH phenyl, exchangeable); 9.59 (bs, 1H, NH hydraz., exchangeable). ^13^C NMR (101 MHz, CDCl_3_): δ 55.26; 55.47; 73.50; 89.27; 114.36; 116.86; 120.71; 126.04; 126.18; 128.16; 128.75; 129.13; 129.32; 130.36; 140.97; 142.71; 145.55; 149.12; 161.45. Calcd for C_26_H_26_N_6_O_3_: C = 66.37; H = 5.57; N = 17.86. Found: C = 66.40; H = 5.30. N = 17.63.

*(E)-5-amino-N′-(3,4-dimethoxybenzylidene)-1-(2-hydroxy-2-phenylethyl)-3-(phenylamino)-1H-pyrazole-4-carbohydrazide* **13b**.

Mp 158–159 °C (EtOH); Yield 65%. ^1^H NMR (400 MHz, DMSO-d_6_): δ 3.74 (s, 3H, OCH_3_); 3.80 (s, 3H, OCH_3_); 3.92–4.12 (m, 2H, CH_2_N); 5.00–5.05 (m, 1H, CHOH); 5.73–5.76 (m, 1H, OH, exchangeable); 6.28 (bs, 2H, NH_2_, exchangeable); 6.75–6.80 (m, 1H, arom. H); 6.98–7.02 (m, 1H, arom. H); 7.12–7.22 (m, 3H, arom. H); 7.27–7.29 (m, 2H, arom. H); 7.32–7.36 (m, 4H, arom. H); 7.42–7.46 (m, 2H, arom. H); 8.09 (s, 1H, CH=C); 8.86 (bs, 1H, NH phenyl, exchangeable); 10.56 (bs, 1H, NH hydraz., exchangeable). ^13^C NMR (101 MHz, DMSO-d_6_): δ 53.76; 55.29; 55.59; 71.33; 86.03; 107.84; 111.55; 116.13; 119.10; 121.47; 126.35; 126.98; 127.40; 128.14; 128.79; 142.53; 142.77; 144.72; 148.26; 149.11; 149.38; 150.49; 163.07. Calcd for C_27_H_28_N_6_O_4_: C = 64.79; H = 5.64; N = 16.79. Found: C = 64.55; H = 5.76. N = 16.52.

*(E)-5-amino-1-(2-hydroxy-2-phenylethyl)-N′-(4-methoxy-3-phenoxybenzylidene)-3-(phenylamino)-1H-pyrazole-4-carbohydrazide* **13c**.

Mp 144–145 °C (EtOH); Yield 59%. ^1^H NMR (400 MHz, DMSO-d_6_): δ 3.80 (s, 3H, OCH_3_); 3.90–4.10 (m, 2H, CH_2_N); 4.97–5.03 (m, 1H, CHOH); 5.71–5.74 (m, 1H, OH, exchangeable); 6.18 (bs, 2H, NH_2_, exchangeable); 6.73–6.79 (m, 1H, arom. H); 6.87–6.91 (m, 2H, arom. H); 7.03–7.09 (m, 1H, arom. H); 7.14–7.25 (m, 3H, arom. H); 7.27–7.37 (m, 8H, arom. H); 7.39–7.47 (m, 3H, arom. H); 8.09 (s, 1H, CH=C); 8.72 (bs, 1H, NH phenyl, exchangeable); 10.46 (bs, 1H, NH hydraz., exchangeable). ^13^C NMR (101 MHz, DMSO-d_6_): δ 53.69; 55.91; 71.32; 86.12; 113.47; 116.09; 116.69; 118.17; 119.11; 122.69; 124.86; 126.33; 127.39; 127.61; 128.12; 128.78; 129.91; 142.60; 142.71; 144.11; 144.38; 148.12; 149.27; 152.56; 157.35. Calcd for C_32_H_30_N_6_O_4_: C = 68.31; H = 5.37; N = 14.94. Found: C = 68.01; H = 5.15; N = 15.00.

*(E)-5-amino-N′-(3-(benzyloxy)-4-methoxybenzylidene)-1-(2-hydroxy-2-phenylethyl)-3-(phenylamino)-1H-pyrazole-4-carbohydrazide* **13d**.

Mp 170–173 °C (EtOH); Yield 74%. ^1^H NMR (400 MHz, DMSO-d_6_): δ 3.81 (s, 3H, OCH_3_); 3.92–4.11 (m, 2H, CH_2_N); 5.02 (s, 2H, CH_2_Ph); 5.12–5.16 (m, 1H, CHOH); 5.73–5.76 (m, 1H, OH, exchangeable); 6.29 (bs, 2H, NH_2_, exchangeable); 6.74–6.81 (m, 1H, arom. H); 7.02–7.06 (m, 1H, arom. H); 7.16–7.22 (m, 3H, arom. H); 7.32–7.237 (m, 5H, arom. H); 7.38–7.45 (m, 8H, arom. H); 8.08 (s, 1H, CH=C); 8.87 (bs, 1H, NH phenyl, exchangeable); 10.58 (bs, 1H, NH hydraz., exchangeable). ^13^C NMR (101 MHz, DMSO-d_6_): δ 53.78; 55.66; 69.73; 71.33; 85.99; 109.55; 111.86; 116.14; 119.09; 121.66; 123.73; 126.34; 126.92; 127.38; 128.01; 128.11; 128.43; 128.48; 128.79; 136.82; 142.49; 142.75; 148.17; 149.34; 150.75; 151.91; 160.77. Calcd for C_33_H_32_N_6_O_4_: C = 68.73; H = 5.59; N = 14.57. Found: C = 68.78; H = 5.52; N = 14.67.

#### 4.1.3. General Synthetic Procedure for the Synthesis of Pyrazole Amides **14**–**16** and **20**–**22**

To a DCM solution (10 mL) of **1** or **2** (1 mmol), TEA (211 µL, 1.5 mmol) and the suitable acyl chloride (1.2 mmol) were sequentially added. After stirring at rt for 24h, the reaction mixture was washed with saturated NaHCO_3_ (2 × 10 mL), water (1 × 10 mL), and dried with anhydrous Na_2_SO_4_. Evaporating in vacuo gave crude product that was purified by crystallization from the suitable solvent or solvent mixture.

*Methyl 5-(phenylamino)-3-(3,4,5-trimethoxybenzamido)-1H-pyrazole-4-carboxylate* **14**.

Mp 164–166 °C (DCM/MeOH); Yield 55%. ^1^H NMR (400 MHz, DMSO-d_6_): δ 3.85 (s, 12H, OCH_3_); 6.97–7.05 (m, 1H, arom. H); 7.21–7.34 (m, 4H, arom. H); 7.68–7.76 (m, 2H, arom. H); 10.96 (s, 1H, CONH, exchangeable); 11.24 (s, 1H, NH phenyl, exchangeable). Calcd for C_21_H_22_N_4_O_6_: C = 59.15; H = 5.20; N = 13.14. Found: C = 59.40; H = 5.38; N = 12.86.

*Ethyl 3-(4-methoxybenzamido)-5-(phenylamino)-1H-pyrazole-4-carboxylate* **15**.

Mp 155–157 °C (Et_2_O); Yield 65%. ^1^H NMR (400 MHz, DMSO-d_6_): δ 1.35 (t, 3H, *J* = 7.1 Hz, CH_3_); 3.88 (s, 3H, OCH_3_); 4.34 (q, 2H, *J* = 7.1 Hz, CH_2_O); 6.88–6.94 (m, 1H, arom. H); 7.08–7.14 (m, 2H, arom. H); 7.23–7.30 (m, 2H, arom. H); 7.51–7.57 (m, 2H, arom. H); 7.67 (bs, 1H, CONH, exchangeable); 8.13–8.20 (m, 2H, arom. H); 8.25 (bs, 1H, NH phenyl, exchangeable). ^13^C NMR (101 MHz, DMSO-d_6_): δ 14.45; 55.60; 59.83; 82.87; 113.22; 117.17; 120.91; 124.67; 128.94; 133.18; 140.17; 151.07; 153.56; 162.76; 163.78; 168.15. Calcd for C_20_H_20_N_4_O_4_: C = 63.15; H = 5.30; N = 14.73. Found: C = 63.51; H = 5.23; N = 15.11.

*Ethyl 3-(3,4-dimethoxybenzamido)-5-(phenylamino)-1H-pyrazole-4-carboxylate* **16**.

Mp 143–145 °C (EtOH); Yield 71%. ^1^H NMR (400 MHz, DMSO-d_6_): δ 1.35 (t, 3H, *J* = 7.1 Hz, CH_3_); 3.79 (s, 3H, OCH_3_); 3.88 (s, 3H, OCH_3_); 4.34 (q, 2H, *J* = 7.1 Hz, CH_2_O); 6.87–6.96 (m, 1H, arom. H); 7.10–7.18 (m, 1H, arom. H); 7.21–7.30 (m, 2H, arom. H); 7.53–7.60 (m, 2H, arom. H); 7.69 (bs, 1H, CONH, exchangeable); 7.78–7.88 (m, 2H, arom. H); 8.30 (bs, 1H, NH phenyl, exchangeable). ^13^C NMR (101 MHz, DMSO-d_6_): δ 14.45; 55.48; 55.76; 59.87; 82.86; 110.64; 114.08; 117.15; 121.00; 124.53; 125.33; 128.90; 140.18; 147.46; 151.18; 152.65; 153.67; 163.80; 168.08. Calcd for C_21_H_22_N_4_O_5_: C = 61.46; H = 5.40; N = 13.65. Found: C = 61.08; H = 5.07; N = 13.72.

*Ethyl 3-(2,6-dimethoxybenzamido)-1-methyl-5-(phenylamino)-1H-pyrazole-4-carboxylate* **20**.

Mp 158–160 °C (DCM/MeOH); Yield 30%. ^1^H NMR (400 MHz, DMSO-d_6_): δ 0.92 (t, 3H, *J* = 7.1 Hz, CH_3_); 3.57 (s, 3H, CH_3_N); 3.78 (s, 6H, 2 x OCH_3_); 3.96 (q, 2H, *J* = 7.1 Hz, CH_2_); 6.66–6.75 (m, 3H, arom. H); 6.78–6.84 (m, 1H, arom. H); 7.15–7.22 (m, 2H, arom. H); 7.33–7.44 (m, 2H, arom. H); 8.18 (bs, 1H, NH phenyl, exchangeable); 9.56 (bs, 1H, NH amide, exchangeable). ^13^C NMR (101 MHz, DMSO-d_6_): δ 14.11; 35.80; 56.34; 56.60; 59.95; 95.10; 104.71; 110.97; 115.44; 120.15; 129.58; 133.28; 142.77; 144.86; 157.38; 157.86; 160.77; 163.44. Calcd for C_22_H_24_N_4_O_5_: C = 62.25; H = 5.70; N = 13.20. Found: C = 62.18; H = 5.53; N = 13.60.

*Ethyl 3-(3,4-dimethoxybenzamido)-1-methyl-5-(phenylamino)-1H-pyrazole-4-carboxylate* **21**.

Mp 186–187 °C (DCM/Et_2_O); Yield 39%. ^1^H NMR (400 MHz, DMSO-d_6_): δ 0.86 (t, 3H, *J* = 7.1 Hz, CH_3_); 3.59 (s, 3H, CH_3_N); 3.82 (s, 3H, OCH_3_); 3.83 (s, 3H, OCH_3_); 3.91 (q, 2H, *J* = 7.1 Hz, CH_2_); 6.63–6.70 (m, 3H, arom. H); 6.76–6.85 (m, 1H, arom. H); 7.04–7.11 (m, 1H, arom. H); 7.15–7.24 (m, 2H, arom. H); 7.51–7.62 (m, 2H, arom. H); 8.21 (bs, 1H, NH phenyl, exchangeable); 10.13 (bs, 1H, NH amide, exchangeable). ^13^C NMR (101 MHz, DMSO-d_6_): δ 13.70; 35.34; 55.57; 55.66; 59.18; 98.82; 110.79; 110.99; 114.71; 119.56; 120.96; 126.17; 129.13; 142.95; 144.49; 145.20; 148.31; 151.70; 162.09; 164.74. Calcd for C_22_H_24_N_4_O_5_: C = 62.25; H = 5.70; N = 13.20. Found: C = 62.18; H = 5.37; N = 13.08.

*Ethyl 1-methyl-5-(phenylamino)-3-(3,4,5-trimethoxybenzamido)-1H-pyrazole-4-carboxylate* **22**.

Mp 135–138 °C (Et_2_O); Yield 22%. ^1^H NMR (400 MHz, DMSO-d_6_): δ 0.87 (t, 3H, *J* = 7.1 Hz, CH_3_); 3.59 (s, 3H, CH_3_N); 3.73 (s, 3H, OCH_3_); 3.85 (s, 6H, 2 x OCH_3_); 3.91 (q, 2H, *J* = 7.1 Hz, CH_2_); 6.63–6.70 (m, 2H, arom. H); 6.76–6.85 (m, 1H, arom. H); 7.16–7.24 (m, 2H, arom. H); 7.28–7.31 (m, 2H, arom. H); 8.21 (bs, 1H, NH phenyl, exchangeable); 10.21 (bs, 1H, NH amide, exchangeable). ^13^C NMR (101 MHz, DMSO-d_6_): δ 13.72; 35.35; 56.03; 59.16; 60.15; 99.35; 105.13; 114.67; 119.57; 129.13; 140.33; 143.03; 144.51; 144.87; 152.66; 161.88; 164.87. Calcd for C_23_H_26_N_4_O_6_: C = 60.78; H = 5.77; N = 12.33. Found: C = 60.97; H = 5.67; N = 12.65.

#### 4.1.4. Synthesis of Pyrazole Amides **17** and **18**

To a dry DMF solution (5 mL) of pyrazole **3** (266 mg, 1 mmol), TMEDA (169 µL, 1.1 mmol) and the proper acyl chloride (1.1 mmol) were sequentially added. After stirring at 120 °C for 2 h, the reaction mixture was cooled at rt, and water (40 mL) was added. The precipitated solid was collected by filtration and recrystallized from the proper solvent or solvent mixture.

*Ethyl 3-(cyclopropanecarboxamido)-1-methyl-5-(phenylamino)-1H-pyrazole-4-carboxylate* **17**.

Mp 144–145 °C (EtOH); Yield 43%. ^1^H NMR (400 MHz, CDCl_3_): δ 0.82–0.93 (m, 2H, CH_2_-cycloprop); 1.08–1.18 (m, 2H, CH_2_-cycloprop); 1.33 (t, 3H, *J* = 7.1 Hz, CH_3_); 1.44–1.74 (m, 1H, CHCO); 3.46 (s, 3H, CH_3_N); 4.30 (q, 2H, *J* = 7.1 Hz, CH_2_O); 6.75–6.85 (m, 3H, arom. H + NH amide, exchangeable); 6.99–7.08 (m, 1H, arom. H); 7.23–7.35 (m, 2H, arom. H); 9.18 (bs, 1H, NH phenyl, exchangeable). Calcd for C_17_H_20_N_4_O_3_: C = 62.18; H = 6.14; N = 17.06. Found: C = 61.86; H = 5.96; N = 16.65.

*Ethyl 3-(4-chlorobenzamido)-1-methyl-5-(phenylamino)-1H-pyrazole-4-carboxylate* **18**.

Mp 158–161 °C (Et_2_O/ligroin); Yield 36%. ^1^H NMR (400 MHz, DMSO-d_6_): δ 0.85 (t, 3H, *J* = 7.1 Hz, CH_3_); 3.59 (s, 3H, CH_3_N); 3.91 (q, 2H, *J* = 7.1 Hz, CH_2_); 6.62–6.70 (m, 2H, arom. H); 6.75–6.86 (m, 1H, arom. H); 7.12–7.24 (m, 2H, arom. H); 7.55–7.65 (m, 2H, arom. H); 7.94–7.99 (m, 2H, arom. H); 8.23 (bs, 1H, NH phenyl, exchangeable); 10.34 (bs, 1H, NH amide, exchangeable). ^13^C NMR (101 MHz, DMSO-d_6_): δ 13.69; 35.40; 59.21; 98.96; 114.76; 119.62; 128.66; 129.14; 129.54; 132.74; 136.67; 143.12; 144.41; 144.77; 161.93; 164.45. Calcd for C_20_H_19_ClN_4_O_3_: C = 60.23; H = 4.80; N = 14.05. Found: C = 59.87; H = 4.81; N = 14.39.

#### 4.1.5. Synthesis of Ethyl 3-(4-Methoxybenzamido)-1-methyl-5-(phenylamino)-1H-pyrazole-4-carboxylate **19**

To a dry ACN solution (10 mL) of **3** (266 mg, 1 mmol), TEA (214 µL, 1.5 mmol), and *p*-methoxybenzoyl chloride (164 µL, 1.2 mmol) dissolved in dry ACN (2 mL) were sequentially added. After stirring at rt for 72 h, the reaction mixture was refluxed for 0.5 h. After cooling at rt, the solvent was evaporated in vacuo and saturated NaHCO_3_ (10 mL) was added. The mixture was extracted with DCM (2 × 10 mL), and the pooled organic phases were washed with water (1 × 10 mL), dried and filtered. Evaporating in vacuo gave a crude residue, which was purified by column chromatography (silica gel, eluent: Et_2_O-Et_2_O/5% EtOH). 

Mp 143–145 °C (Et_2_O); Yield 30%. ^1^H NMR (400 MHz, DMSO-d_6_): δ 0.85 (t, 3H, *J* = 7.1 Hz, CH_3_); 3.58 (s, 3H, CH_3_N); 3.83 (s, 3H, OCH_3_); 3.91 (q, 2H, *J* = 7.1 Hz, CH_2_); 6.63–6.71 (m, 2H, arom. H); 6.76–6.85 (m, 1H, arom. H); 7.02–7.10 (m, 2H, arom. H); 7.15–7.24 (m, 2H, arom. H); 7.90–7.97 (m, 2H, arom. H); 8.21 (bs, 1H, NH phenyl, exchangeable); 10.11 (bs, 1H, NH amide, exchangeable). ^13^C NMR (101 MHz, DMSO-d_6_): δ 13.67; 35.34; 55.44; 59.19; 98.46; 113.75; 114.75; 119.58; 126.15; 129.12; 129.48; 142.93; 144.45; 145.30; 162.04; 162.19; 164.57. Calcd for C_21_H_22_N_4_O_4_: C = 63.95; H = 5.62; N = 14.20. Found: C = 63.56; H = 5.55; N = 14.39.

### 4.2. Biology

#### 4.2.1. MTT Assays

All reagents were purchased from EuroClone, Milan, Italy). The following cell lines were used for MTT assays: SKOV-3 (ovarian adenocarcinoma, ATCC, Manassas, VA, USA); MCF-7 (breast adenocarcinoma, Biologic Bank and Cell Factory, IRCCS Policlinico San Martino, Genoa, Italy); Hep-G2 (hepatocellular carcinoma, ATCC, Manassas, VA, USA); SK-MEL28 (skin melanoma, Biologic Bank and Cell Factory, IRCCS Policlinico San Martino, Genoa, Italy), GM-6114 (embryonic human fibroblast, ATCC, Manassas, VA, USA); MDA-MB231 (breast adenocarcinoma, Biologic Bank and Cell Factory, IRCCS Policlinico San Martino, Genoa, Italy); HeLa (cervical adenocarcinoma, Biologic Bank and Cell Factory, IRCCS Policlinico San Martino, Genoa, Italy); SK-BR3 (breast andenocarcinoma, Biologic Bank and Cell Factory, IRCCS Policlinico San Martino, Genoa, Italy); A549 (lung carcinoma, Biologic Bank and Cell Factory, IRCCS Policlinico San Martino, Genoa, Italy); HUVEC (Human Umbilical Vein Endothelial Cells, ATCC, Manassas, VA, USA). All cell lines were grown in their medium with 10% FBS, 2 mM Glutamine, and 1% penstrep and incubated at 37 °C in 5% CO_2_ in a humidified environment. The cell lines were plated in 96-well plates at an adequate number to reach 80–90% confluence at the end of the assay. 16 h after cell plating, a 10 mM DMSO stock solution of the compounds was diluted in growth medium and added at a final working concentration of 10 μM. After 48h of incubation, a 2 mg/mL PBS solution of MTT (3-(4,5-dimethyl-2-thiazolyl)-2,5-diphenyl-2*H*-tetrazolium bromide) was added (30 μL/well). After 4h, the supernatant was removed, and the Formazan precipitates were dissolved in DMSO (100 μL/well). The 96-well plates were incubated for 20 min, and absorbance was measured at 570 nm using a plate reader. The results are expressed as a percentage ratio over control samples (100%) in which the cells were incubated with the same amount of DMSO but without compounds. Each value is the mean of three independent experiments run in six replicates. 

The IC_50_ values were extrapolated from nonlinear regression analysis of concentration–response curves (used concentrations: 1, 5, 10 μM), using the MS Excel software (Microsoft 365 suite). Each IC_50_ value is the mean of three independent experiments run in duplicate.

#### 4.2.2. Blood Collection

Freshly drawn venous blood from healthy volunteers from “Centro Trasfusionale” (IRCCS Policlinico San Martino, Genoa, Italy) was collected into a 130 mM aqueous trisodium citrate anticoagulant solution (9:1). The donors claimed to not have taken drugs known to interfere with platelet function during the two weeks prior to blood collection and gave their informed consent. Whole blood was centrifuged at 100× *g* for 20 min to afford platelet-rich plasma that was then spun at 1100× *g* for 15 min. The obtained pellet was washed once with a pH 5.2 ACD solution (75 mM trisodium citrate, 42 mM citric acid, and 136 mM glucose), centrifuged at 1100× *g* for 15 min, and then re-suspended in pH 7.4 Hepes buffer (145 mM NaCl, 5 mM KCl, 1 mM MgSO4, 10 mM glucose, and 10 mM HEPES).

#### 4.2.3. ROS Assay

2′,7′-Dichlorofluorescein diacetate (DCFH-DA) and thrombin were purchased from Sigma-Aldrich/Merck Millipore. DMSO solutions of **10**–**22** were diluted in saline immediately before each experiment. ROS production was quantified by DCFH-DA, a ROS-sensitive probe that yields, upon oxidation, the fluorescent adduct DCF that is trapped inside the cells [55]. Briefly, washed platelets (1.0 × 10^8^/mL), pre-incubated with saline solutions of **10**–**22** for 15 min at 37 °C, were stimulated by 0.1 U/mL thrombin. Incubation was stopped by cooling samples in an ice bath, and then samples were immediately analyzed in a Merck Millipore Bioscience Guava easyCyte flow cytometer (Merk Millipore, Burlington, MA, USA). The reported IC_50_ values represent the molar concentration of the compounds able to inhibit 50% of the maximal aggregation induced by the agonist and are calculated as the percentage inhibition of the maximal aggregation measured in the presence of the agent compared with the measure in a control sample containing saline, carried out under the same conditions. The IC_50_ values were extrapolated from nonlinear regression analysis of concentration–response curves (three points) using MS Excel software (Microsoft 365 suite). Each IC_50_ value is the mean of six independent experiments. 

#### 4.2.4. Platelet Aggregation

Thrombin was purchased from Sigma-Aldrich/Merck Millipore. A DMSO solution of compounds **10**–**22** was diluted in saline immediately before each experiment and added to the washed platelets (3.0 × 10^8^/mL) at 37 °C. After 3 min, 0.1 U/mL thrombin was added, and platelet aggregation was quantified according to Born’s method [56] using a Bio-Data Aggregometer (Bio-Data Corporation, Horsham, PA, USA). The IC_50_ values were calculated as detailed above. 

### 4.3. DPPH Radical-Scavenging Activity

Compounds **10b**, **11a**, **11d**, **12d**, **13d**, **14**, and **22** (ca. 3 mg) were dissolved in DMSO (1 mL), and then 100 µL of this solution was mixed with 3.9 mL of DPPH methanol solution (65 µM). Absorbance was measured at 517 nm after reacting for 30 min in the dark. The linear calibration curve was obtained using Trolox standards (ranging between 20 and 200 mg/L, R^2^ = 0.9955). The result was calculated as Trolox equivalent in mg/L, and the percentage of antioxidant activity (AA%) was calculated from the ratio of decreasing absorbance of sample solution (A0 − As) to absorbance of blank DPPH solution (A0), as expressed in Equation (1) [57,58].

(1) $$AA\%=\frac{\left(A0-As\right)}{A0}\times 100$$

All analyses were carried out in duplicate (*n* = 2), and values are given as means ± standard deviation (SD).

## 5. Conclusions

To further extend the SARs of antioxidant derivatives **IV** and **V**, pyrazolyl acylhydrazones **10**–**13** and amides **14**–**22** were prepared from APs **1**–**5** through a divergent approach. The novel compounds were evaluated for (i) antiproliferative activity in cell-based assays; (ii) antioxidant and antiaggregating properties in platelets; and (iii) anti-scavenging efficacy. Compound **11a** displayed micromolar IC_50_ values against selected tumor cell lines (namely, HeLa, MCF7, SKOV3, and SKMEL28 cells), and NCI screening on a large panel of tumor cell lines confirmed the promising cytotoxic activity of this derivative. Different from all its analogues, pyrazolyl amide **14** showed relevant and unexpected antiproliferative activity against melanoma (SKMEL28), lung (A549), and cervical (HeLa) tumors. Unfortunately, the compound was as cytotoxic as cisplatin against GM6114 normal fibroblasts. Despite resulting in less activity compared to lead compounds **IV** and **V**, selected pyrazole acylhydrazones and amides significantly inhibited aggregation and ROS production in platelets and proved to be more effective than ASA and NAC. Moreover, the antiproliferative activity does not seem to correlate with the antioxidant/antiaggregant values. Finally, DPPH experiments indicate relevant radical scavenging properties of acylhydrazones, which can, therefore, represent a privilege scaffold for the development of novel antiproliferative and antioxidant agents.

## Figures and Tables

**Table 1:** Antiproliferative activity of pyrazole acylhydrazones **10**–**22**.

	Mean Growth Percentage ^a^								
Cpd	MCF7	MDA-MB231	SK-BR3	SKMEL28	SKOV3	Hep-G2	A549	HeLa	GM-6114
**10a**	95.06	99.11	95.51	86.73	103.95	115.01	103.47	98.41	110.40
**10b**	95.34	100.35	91.61	94.45	114.49	115.09	91.07	105.04	107.36
**11a**	33.56	71.95	56.96	49.44	43.60	75.04	60.77	25.00	69.81
**11b**	94.77	85.73	86.00	93.00	100.15	116.18	94.84	99.47	104.95
**11c**	84.53	93.38	83.07	87.64	109.68	113.17	91.74	101.22	105.27
**11d**	82.90	98.59	90.21	89.00	102.55	111.19	100.95	95.15	99.22
**12a**	88.54	97.88	94.98	76.17	101.78	110.74	97.14	94.80	100.38
**12b**	100.63	91.72	94.24	73.31	98.59	92.72	95.30	92.81	99.78
**12c**	88.28	100.47	82.13	94.56	104.19	111.34	98.46	85.20	104.08
**12d**	85.17	94.14	90.59	80.78	106.17	105.32	102.33	85.68	102.79
**13a**	80.99	96.39	91.82	81.84	93.80	85.94	94.48	83.66	99.43
**13b**	101.49	92.11	92.24	95.69	113.65	106.33	104.07	98.84	106.86
**13c**	99.75	103.87	87.45	103.71	128.71	124.76	94.38	97.88	99.14
**13d**	107.57	93.75	97.87	93.89	111.82	119.55	90.40	96.65	95.39
**14**	66.12	56.98	53.75	41.49	38.51	67.24	35.10	9.45	39.31
**15**	65.94	129.84	99.76	71.65	88.37	85.48	67.91	104.70	68.40
**16**	62.39	95.97	82.54	54.02	66.87	49.15	52.58	57.65	53.68
**17**	92.75	127.34	106.09	85.89	86.92	102.74	75.80	108.52	65.92
**18**	63.52	140.96	80.87	68.43	96.88	78.47	69.64	91.46	72.99
**19**	95.25	136.09	103.30	85.18	97.34	105.12	92.92	123.77	66.69
**20**	92.09	123.59	106.45	83.62	84.42	108.07	70.83	110.92	64.71
**21**	90.28	149.42	107.39	84.59	90.24	97.90	112.33	108.07	85.88
**22**	114.93	140.34	103.43	84.12	94.16	102.14	105.54	108.73	57.94
**CisPt**	72.74	86.07	70.59	44.40	36.83	38.07	59.09	29.33	39.52

^a^ Data mean values for three separate experiments; variation among triplicate samples was less than 10%.

**Table 2:** NCI screening of compounds **11a** and **17**. Negative values indicate lethality.

	Growth Percentage (%)				Growth Percentage (%)	
Panel/Cell Line	11a	17		Panel/Cell Line	11a	17
*Leukemia*				*Ovarian Cancer*		
CCRF-CEM	38.45	97.75		IGROV1	39.64	100.75
HL-60(TB)	15.46	96.44		OVCAR-4	87.03	99.35
K-562	19.25	96.41		OVCAR-5	76.88	99.49
MOLT-4	49.41	101.92		OVCAR-8	70.80	101.92
RPMI-8226	61.65	99.62		NCI/ADR-RES	32.98	103.29
SR	18.90	94.23		SK-OV-3	49.64	99.73
*Non-Small Cell Lung Cancer*				*Renal Cancer*		
A549/ATCC	57.09	101.59		786-0	57.97	101.26
EKVX	69.71	96.82		A498	33.08	92.87
HOP-62	38.79	110.36		ACHN	49.99	104.77
HOP-92	46.79	81.47		CAKI-1	39.84	86.39
NCI-H226	51.61	98.86		RXF 393	37.26	101.07
NCI-H23	66.86	98.44		SN12C	57.59	104.27
NCI-H322M	89.96	94.41		TK-10	89.16	107.78
NCI-H460	14.63	101.02		UO-31	51.18	87.45
NCI-H522	19.34	97.23		*Prostate Cancer*		
*Colon Cancer*				PC-3	55.90	95.84
COLO 205	35.82	106.91		DU-145	76.34	104.67
HCC-2998	59.18	114.15		*Breast Cancer*		
HCT-116	14.99	104.12		MCF7	17.56	92.37
HCT-15	19.10	96.95		MDA-MB-231/ATCC	65.06	100.37
HT29	18.58	99.47		HS 578T	3.81	96.86
KM12	27.82	105.43		BT-549	14.04	118.46
SW-620	16.01	94.82		T-47D	42.38	93.07
*CNS Cancer*				MDA-MB-468	−12.56	102.11
SF-268	58.65	101.17		*Melanoma*		
SF-295	98.52	102.51		LOX IMVI	18.93	102.01
SF-539	40.20	97.42		MALME-3M	44.75	98.67
SNB-19	36.78	98.39		M14	13.00	102.31
SNB-75	35.14	94.70		MDA-MB-435	-31.00	101.76
U251	33.33	100.80		SK-MEL-2	70.70	100.69
				SK-MEL-28	57.48	107.16
				SK-MEL-5	57.21	99.45
				UACC-257	56.35	99.58
				UACC-62	23.72	93.55

**Table 3:** DPPH antioxidant activity of selected acylhydrazone and amide derivatives.

Cpd	A (λ = 517 nm) ^a^	DPPH (%) ^b^	AA (%) ^b^
**10b**	0.2115	23.55 ± 0.39	76.45 ± 0.39
**11a**	0.6685	74.44 ± 2.44	25.56 ± 2.44
**11d**	0.7585	84.47 ± 0.24	15.53 ± 0.24
**12d**	0.2775	30.90 ± 0.39	69.10 ± 0.39
**13d**	0.3925	43.71 ± 1.18	56.29 ± 1.18
**14**	0.8655	96.38 ± 0.08	3.62 ± 0.08
**22**	0.8765	97.61 ± 0.24	2.39 ± 0.24

^a^ Absorbance. ^b^ Mean value ± standard deviation (SD) of two independent experiments (*n* = 2).

## Data Availability

Samples of the compounds are available from the authors.

## Supplementary Materials

The following supporting information can be downloaded at: https://www.mdpi.com/article/10.3390/ijms25094607/s1.
