# Supplementary material for: Structure–Activity Relationship Studies on Highly Functionalized Pyrazole Hydrazones and Amides as Antiproliferative and Antioxidant Agents
Source: Int J Mol Sci. 2024 Apr 23;25(9):4607. doi: 10.3390/ijms25094607 (PMC11083148; doi:10.3390/ijms25094607)
Supplement: Supplementary file 1 [file ijms-25-04607-s001.zip › ijms-2964253-supplementary.pdf]

Supporting Information

Structure-activity relationship studies on highly functionalized pyrazole hydrazones and amides as antiproliferative and antioxidant agents

Matteo Lusardi [1], Maria Grazia Signorello [1], Eleonora Russo [1], Debora Caviglia[1], Marco Ponassi [2], Erika Iervasi [2], Camillo Rosano [2], Chiara Brullo [1] and Andrea Spallarossa [1,*]

[1] Department of Pharmacy, University of Genova, viale Benedetto XV, 3, 16132, Genova, Italy; matteo.lusardi@edu.unige.it (M.L.); mariagrazia.signorello@unige.it (M.G.S); chiara.brullo@unige.it (C.B.); eleonora.russo@unige.it (E.R.); debora.caviglia@edu.unige.it (D.C.).

[2] Proteomics and Mass Spectrometry Unit, IRCCS Ospedale Policlinico San Martino, Largo R. Benzi 10, 16132, Genova, Italy; erika.iervasi@hsanmartino.it (E.I.); camillo.rosano@hsanmartino.it (C.R.); marco.ponassi@hsanmartino.it (M.P.).

*Correspondence: andrea.spallarossa@unige.it

Table of contents

Figure S1. ^{1}H-NMR (400 MHz, d_6-DMSO) spectrum of compound **10a**

Figure S2. ^{13}C-NMR (101 MHz, d_6-DMSO) spectrum of compound **10a**

Figure S3. ^{1}H-NMR (400 MHz, d_6-DMSO) spectrum of compound **10b**

Figure S4. ^{13}C-NMR (101 MHz, d_6-DMSO) spectrum of compound **10b**

Figure S5. ^{1}H-NMR (400 MHz, d_6-DMSO) spectrum of compound **11a**

Figure S6. ^{13}C-NMR (101 MHz, d_6-DMSO) spectrum of compound **11a**

Figure S7. ^{1}H-NMR (400 MHz, d_6-DMSO) spectrum of compound **11b**

Figure S8. ^{13}C-NMR (101 MHz, d_6-DMSO) spectrum of compound **11b**

Figure S9. ^{1}H-NMR (400 MHz, d_6-DMSO) spectrum of compound **11c**

Figure S10. ^{13}C-NMR (101 MHz, d_6-DMSO) spectrum of compound **11c**

Figure S11. ^{1}H-NMR (400 MHz, d_6-DMSO) spectrum of compound **11d**

Figure S12. ^{13}C-NMR (101 MHz, d_6-DMSO) spectrum of compound **11d**

Figure S13. ^{1}H-NMR (400 MHz, d_6-DMSO) spectrum of compound **12a**

Figure S14. ^{13}C-NMR (101 MHz, d_6-DMSO) spectrum of compound **12a**

Figure S15. ^{1}H-NMR (400 MHz, d_6-DMSO) spectrum of compound **12b**

Figure S16. ^{13}C-NMR (101 MHz, d_6-DMSO) spectrum of compound **12b**

Figure S17. ^{1}H-NMR (400 MHz, d_6-DMSO) spectrum of compound **12c**

Figure S18. ^{13}C-NMR (101 MHz, d_6-DMSO) spectrum of compound **12c**

Figure S19. ^{1}H-NMR (400 MHz, d_6-DMSO) spectrum of compound **12d**

Figure S20. ^{13}C-NMR (101 MHz, d_6-DMSO) spectrum of compound **12d**

Figure S21. ^{1}H-NMR (400 MHz, d_6-DMSO) spectrum of compound **13a**

Figure S22. ^{13}C-NMR (101 MHz, d_6-DMSO) spectrum of compound **13a**

Figure S23. ^{1}H-NMR (400 MHz, d_6-DMSO) spectrum of compound **13b**

Figure S24. ^{13}C-NMR (101 MHz, d_6-DMSO) spectrum of compound **13b**

Figure S25. ^{1}H-NMR (400 MHz, d_6-DMSO) spectrum of compound **13c**

Figure S26. ^{13}C-NMR (101 MHz, d_6-DMSO) spectrum of compound **13c**

Figure S27. ^{1}H-NMR (400 MHz, d_6-DMSO) spectrum of compound **13d**

Figure S28. ^{13}C-NMR (101 MHz, d_6-DMSO) spectrum of compound **13d**

Figure S29. ^{1}H-NMR (400 MHz, d_6-DMSO) spectrum of compound **14**

Figure S30. ^{1}H-NMR (400 MHz, d_6-DMSO) spectrum of compound **15**

Figure S31. ^{13}C-NMR (101 MHz, d_6-DMSO) spectrum of compound **15**

Figure S32. ^{1}H-NMR (400 MHz, d_6-DMSO) spectrum of compound **16**

Figure S33. ^{13}C-NMR (101 MHz, d_6-DMSO) spectrum of compound **16**

Figure S34. ^{1}H-NMR (400 MHz, d_6-DMSO) spectrum of compound **17**

Figure S35. ^{1}H-NMR (400 MHz, d_6-DMSO) spectrum of compound **18**

Figure S36. ^{13}C-NMR (101 MHz, d_6-DMSO) spectrum of compound **18**

Figure S37. ^{1}H-NMR (400 MHz, d_6-DMSO) spectrum of compound **19**

Figure S38. ^{13}C-NMR (101 MHz, d_6-DMSO) spectrum of compound **19**

Figure S39. ^{1}H-NMR (400 MHz, d_6-DMSO) spectrum of compound **20**

Figure S40. ^{13}C-NMR (101 MHz, d_6-DMSO) spectrum of compound **20**

Figure S41. ^{1}H-NMR (400 MHz, d_6-DMSO) spectrum of compound **21**

Figure S42. ^{13}C-NMR (101 MHz, d_6-DMSO) spectrum of compound **21**

Figure S43. ^{1}H-NMR (400 MHz, d_6-DMSO) spectrum of compound **22**

Figure S44. ^{13}C-NMR (101 MHz, d_6-DMSO) spectrum of compound **22**

Table S1. Inhibitory effect of compounds **10-22** on platelet aggregation and ROS production

Figure S1. ^{1}H-NMR (400 MHz, d_6-DMSO) spectrum of compound **10a**

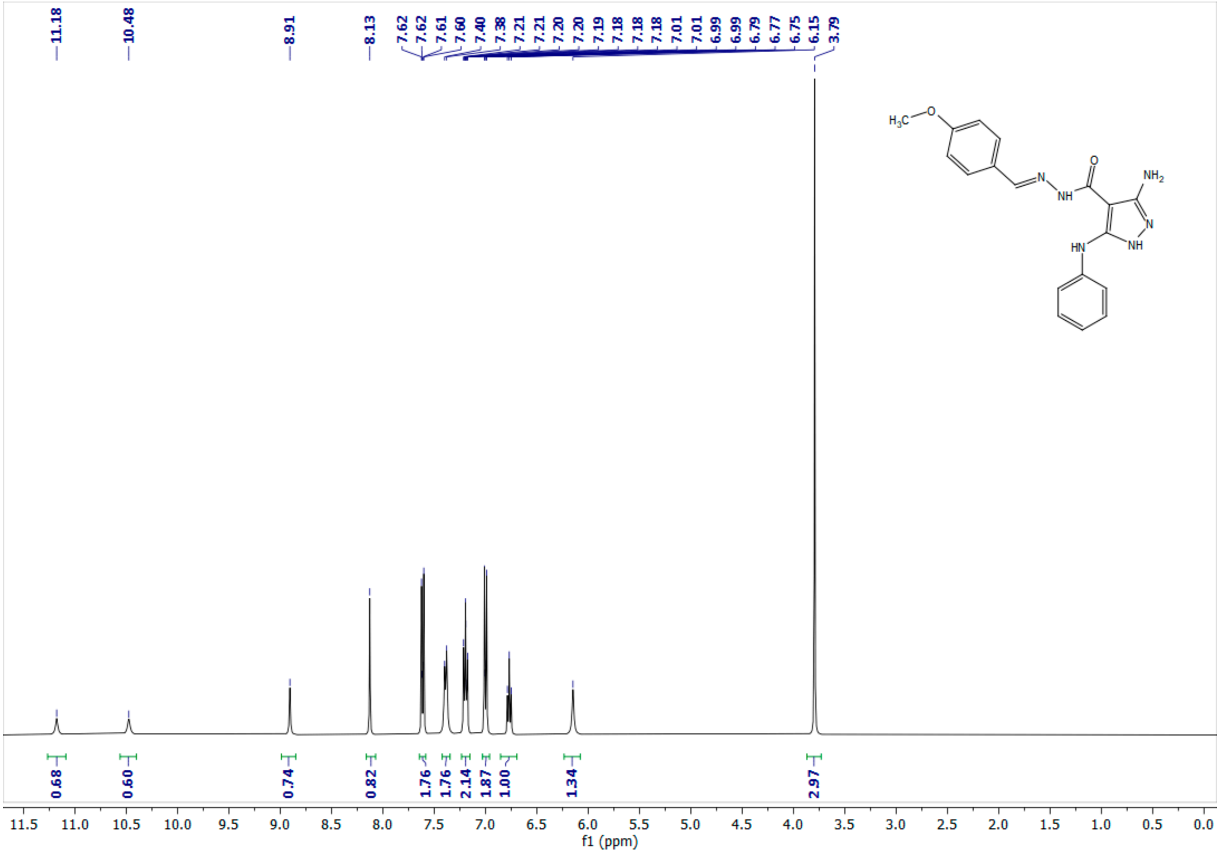

Figure S2. ^{13}C-NMR (101 MHz, d_6-DMSO) spectrum of compound **10a**

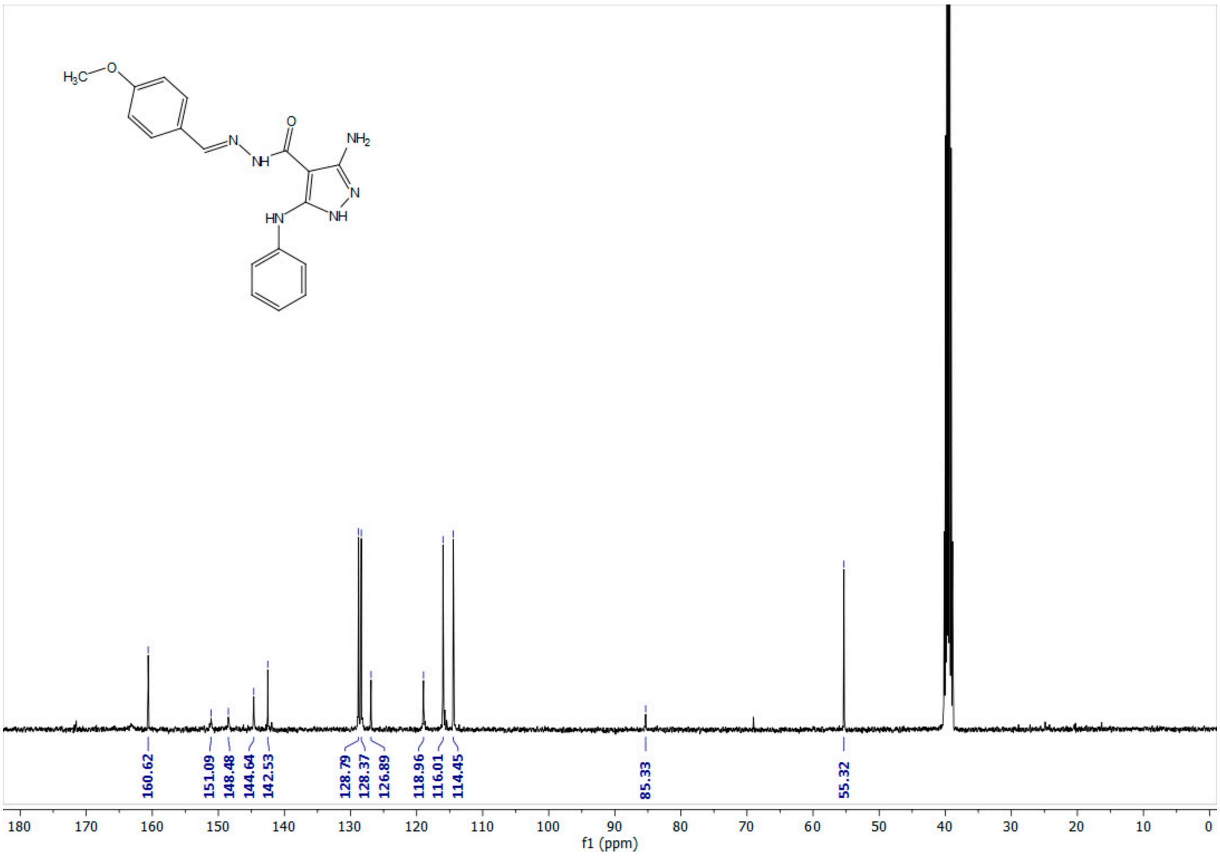

Figure S3. ^{1}H-NMR (400 MHz, d_6-DMSO) spectrum of compound **10b**

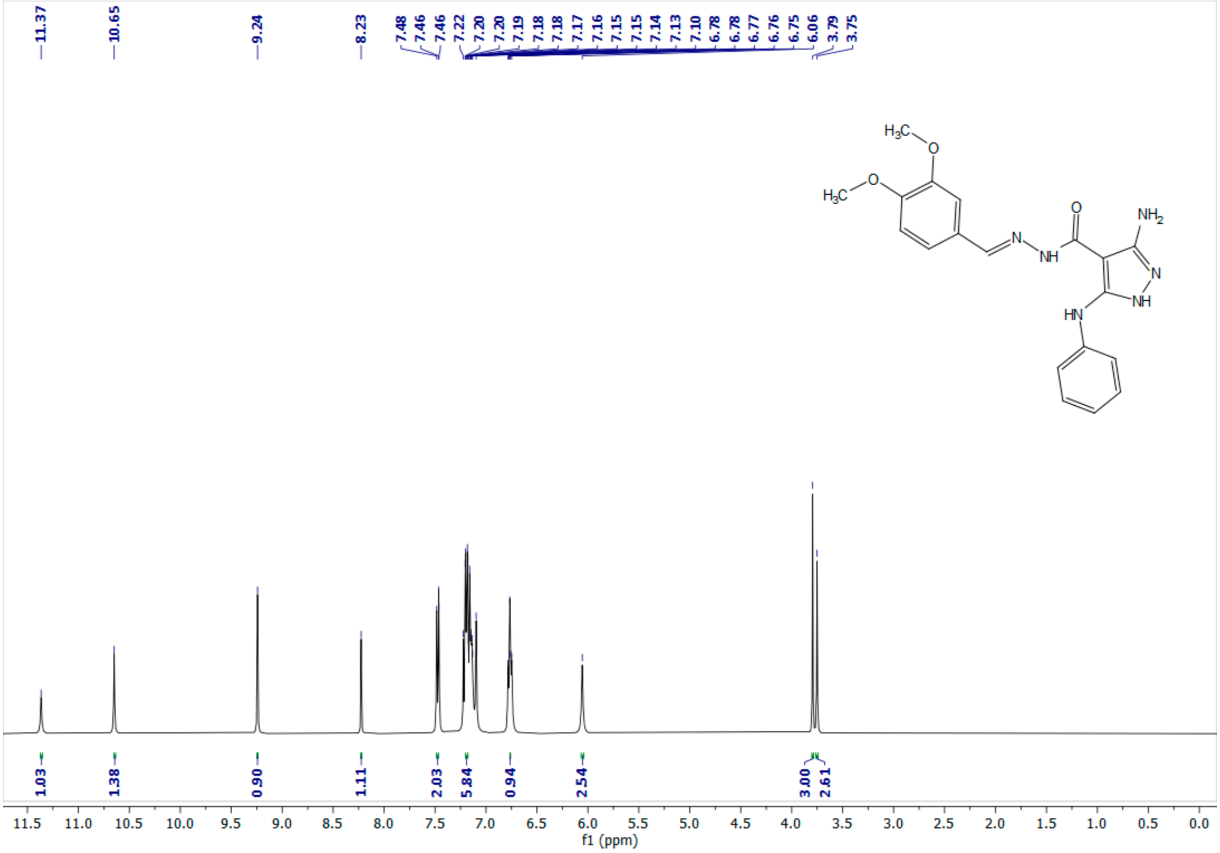

Figure S4. ^{13}C-NMR (101 MHz, d_6-DMSO) spectrum of compound **10b**

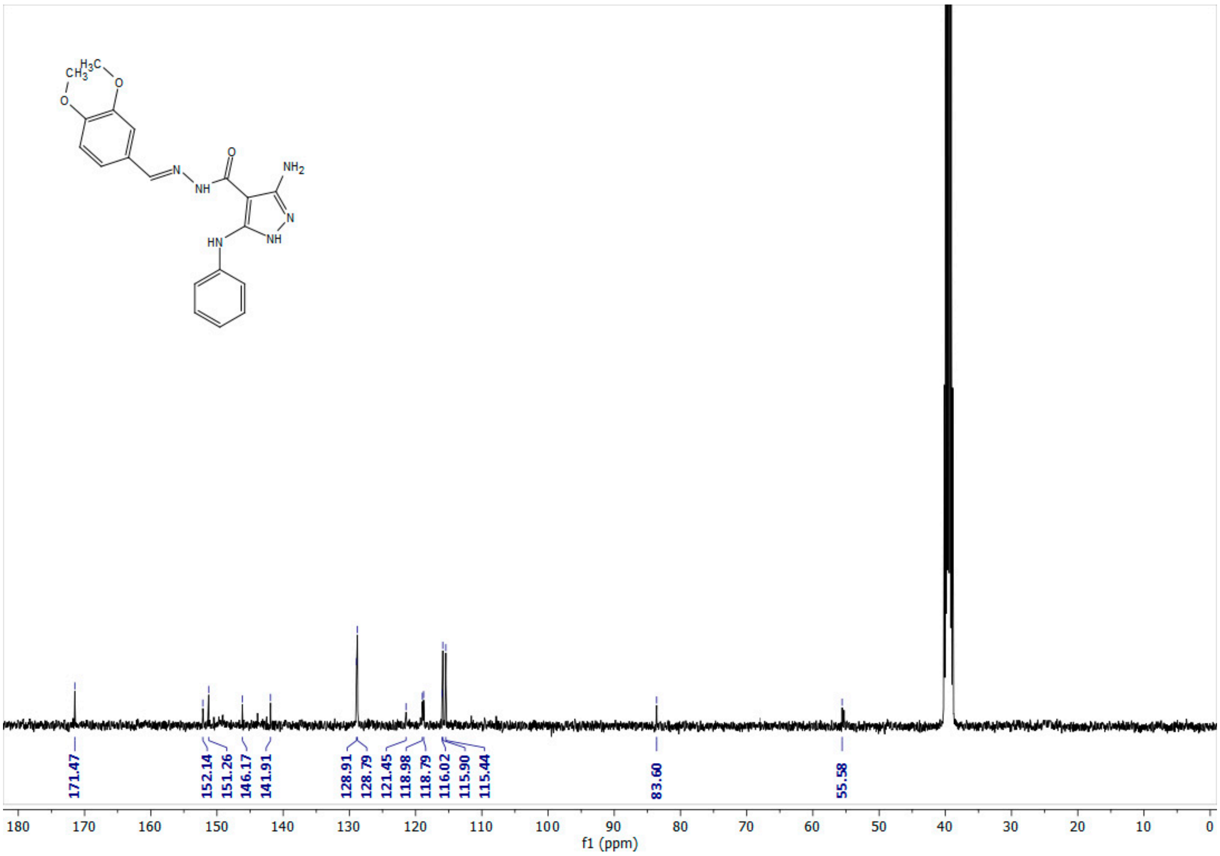

Figure S5. ^{1}H-NMR (400 MHz, d_6-DMSO) spectrum of compound **11a**

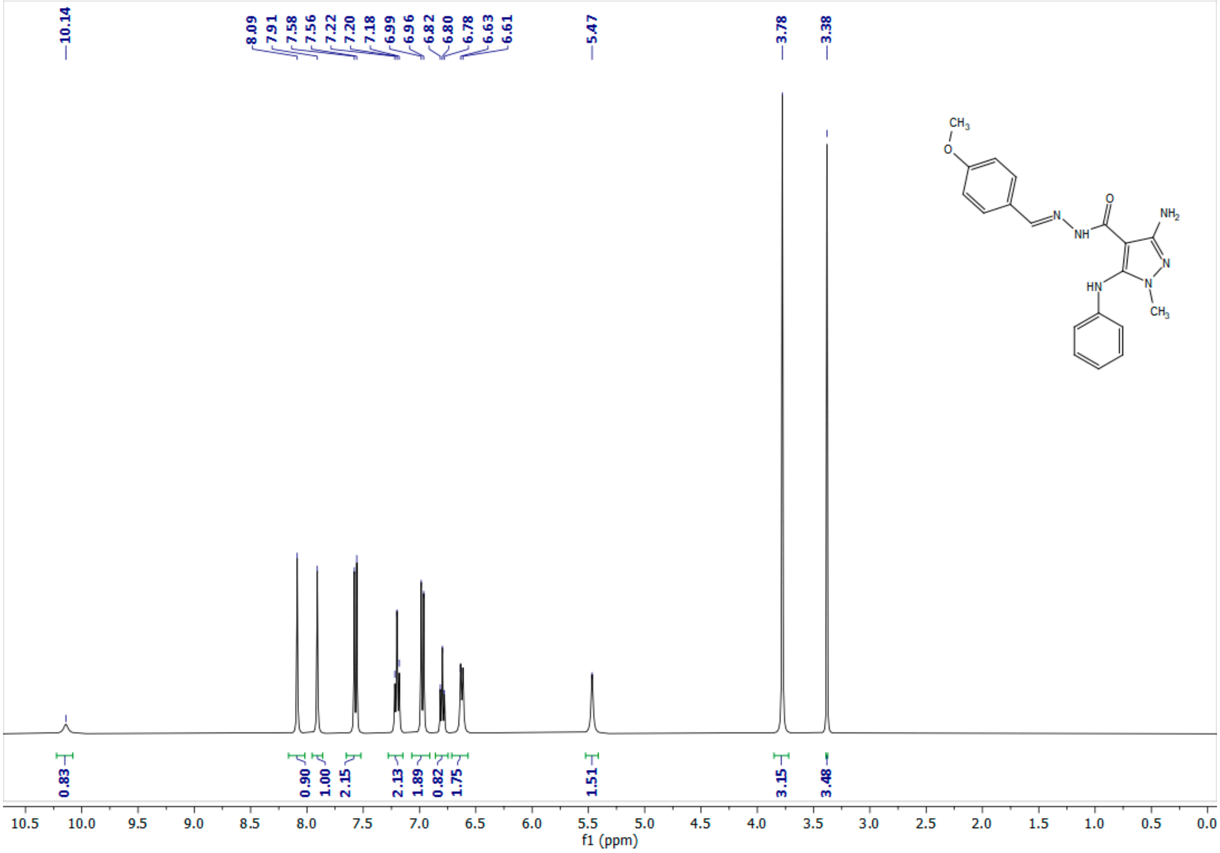

Figure S6. ^{13}C-NMR (101 MHz, d_6-DMSO) spectrum of compound **11a**

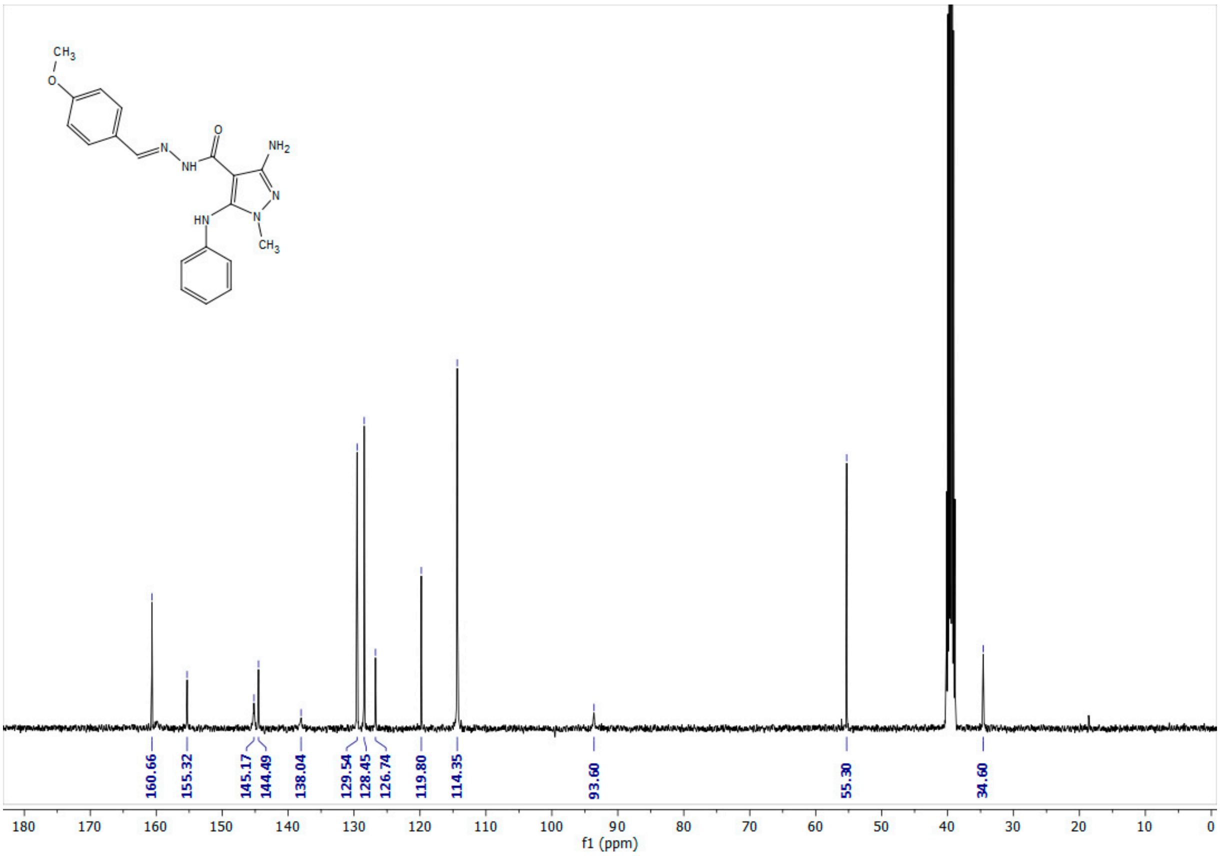

Figure S7. ^{1}H-NMR (400 MHz, d_6-DMSO) spectrum of compound **11b**

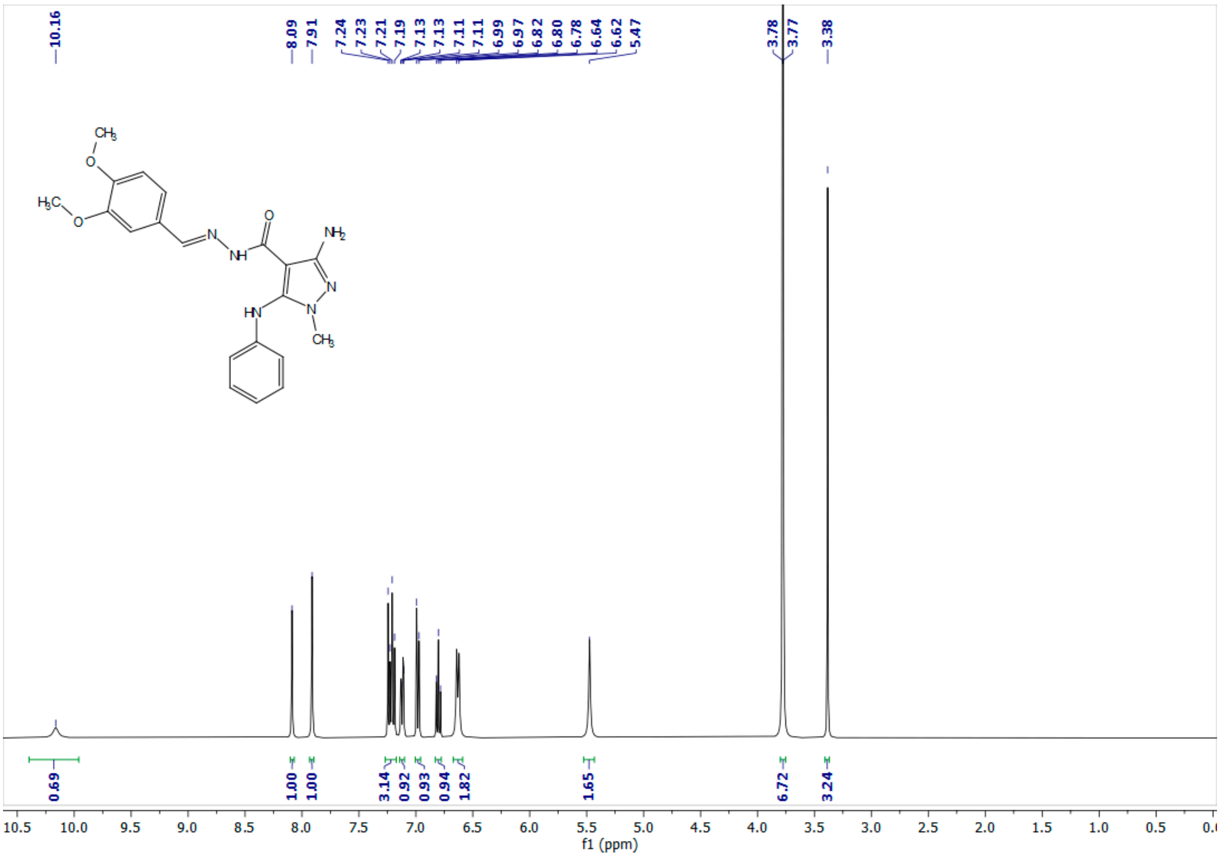

Figure S8. ^{13}C-NMR (101 MHz, d_6-DMSO) spectrum of compound **11b**

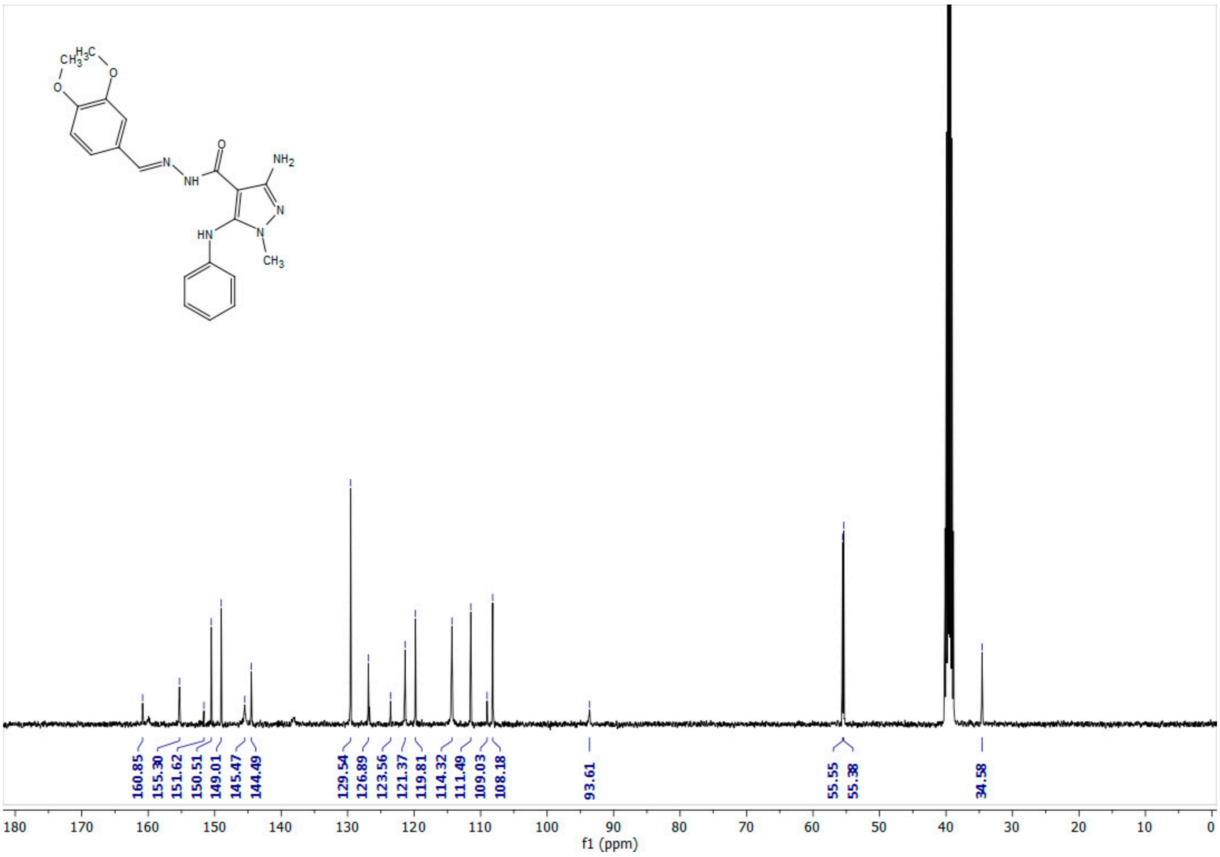

Figure S9. ^{1}H-NMR (400 MHz, d_6-DMSO) spectrum of compound **11c**

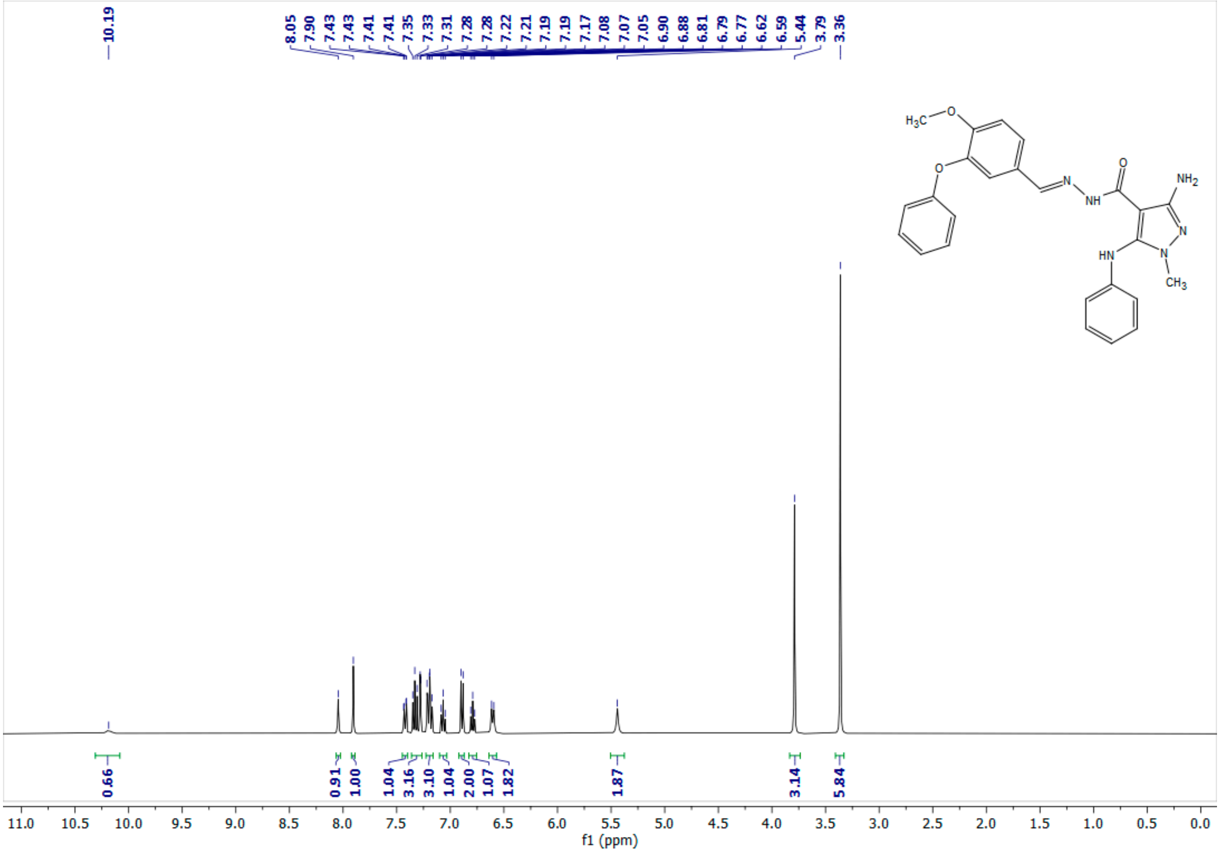

Figure S10. ^{13}C-NMR (101 MHz, d_6-DMSO) spectrum of compound **11c**

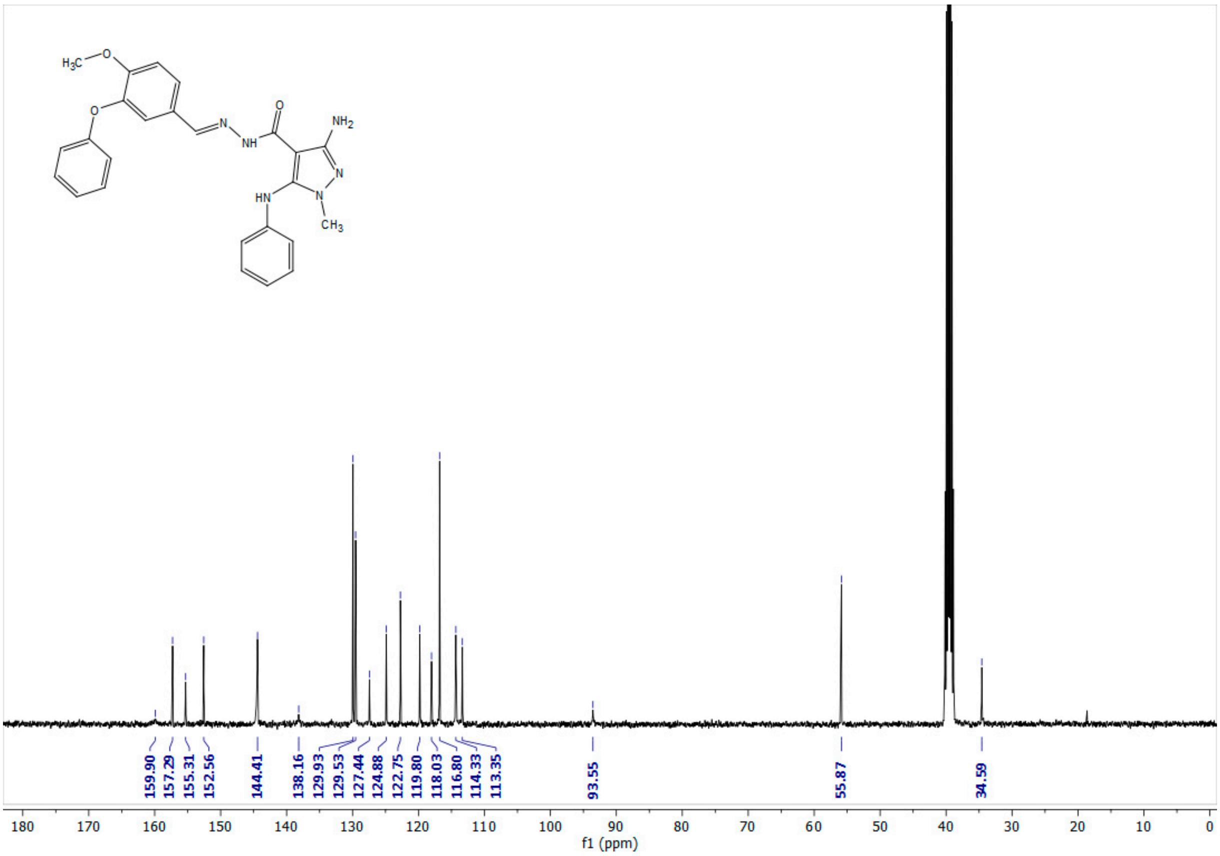

Figure S11. ^{1}H-NMR (400 MHz, d_6-DMSO) spectrum of compound **11d**

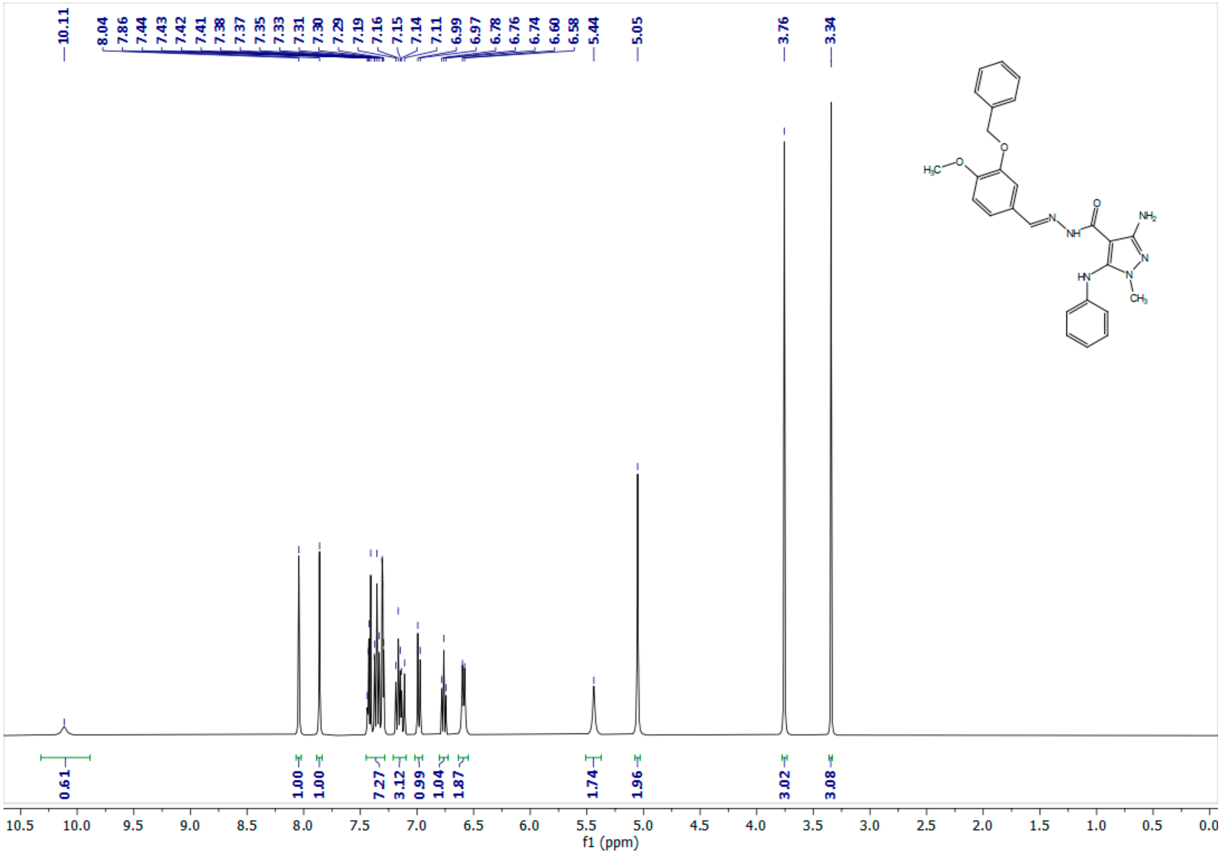

Figure S12. ^{13}C-NMR (101 MHz, d_6-DMSO) spectrum of compound **11d**

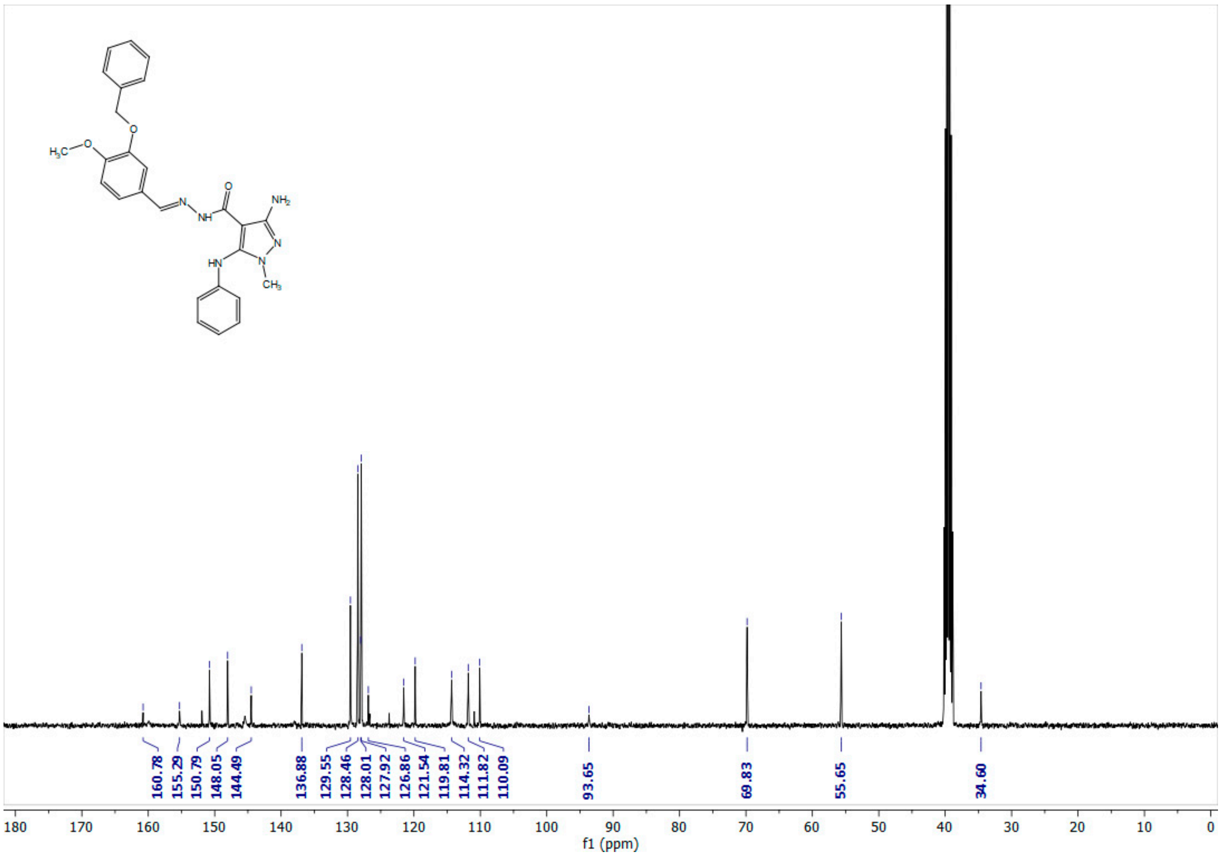

Figure S13. ^{1}H-NMR (400 MHz, d_6-DMSO) spectrum of compound **12a**

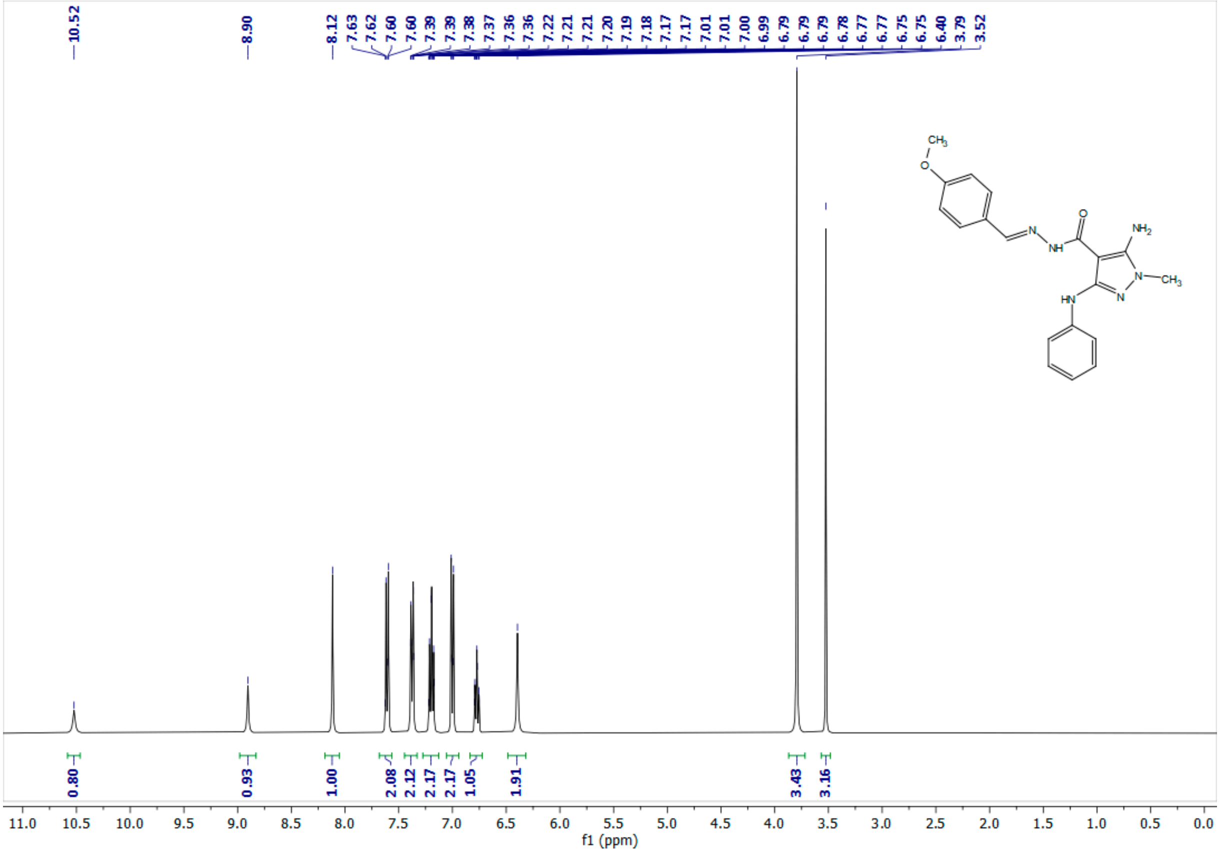

Figure S14. ^{13}C-NMR (101 MHz, d_6-DMSO) spectrum of compound **12a**

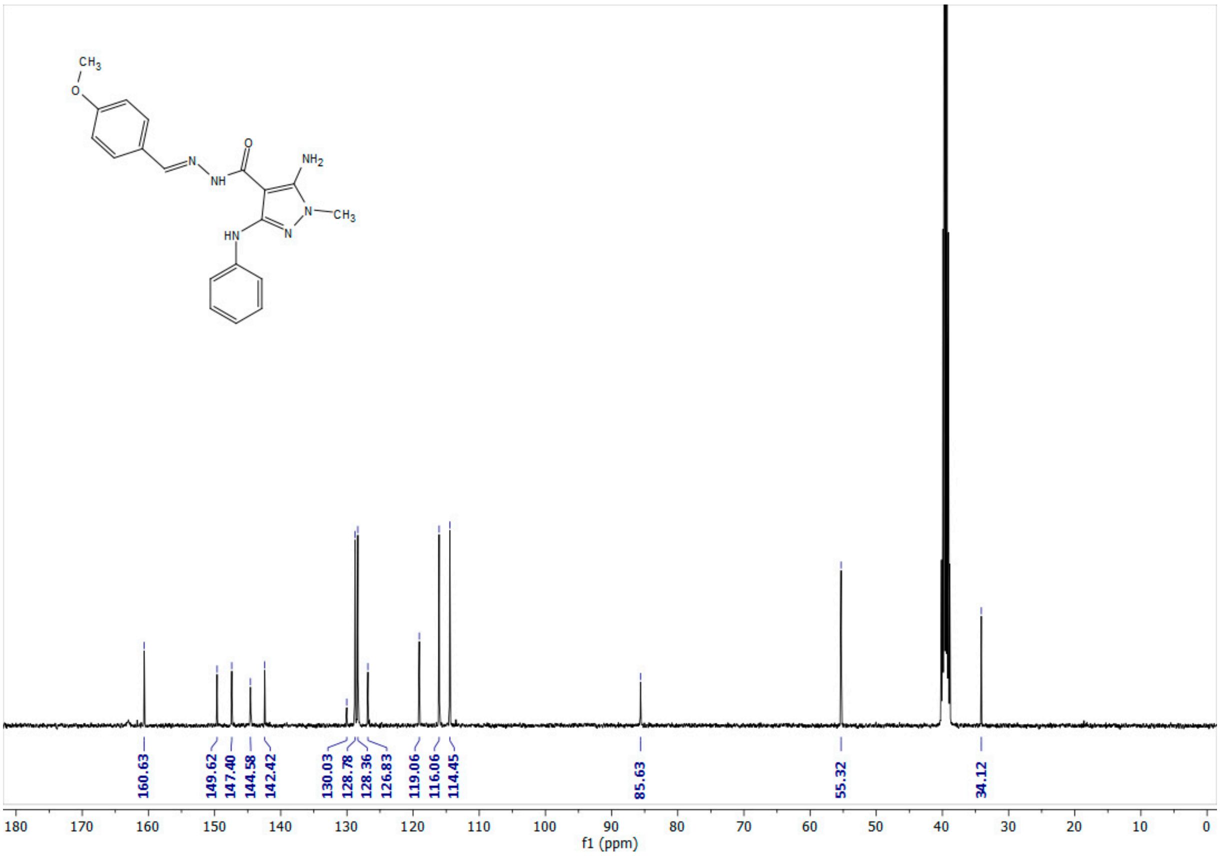

Figure S15. ^{1}H-NMR (400 MHz, d_6-DMSO) spectrum of compound **12b**

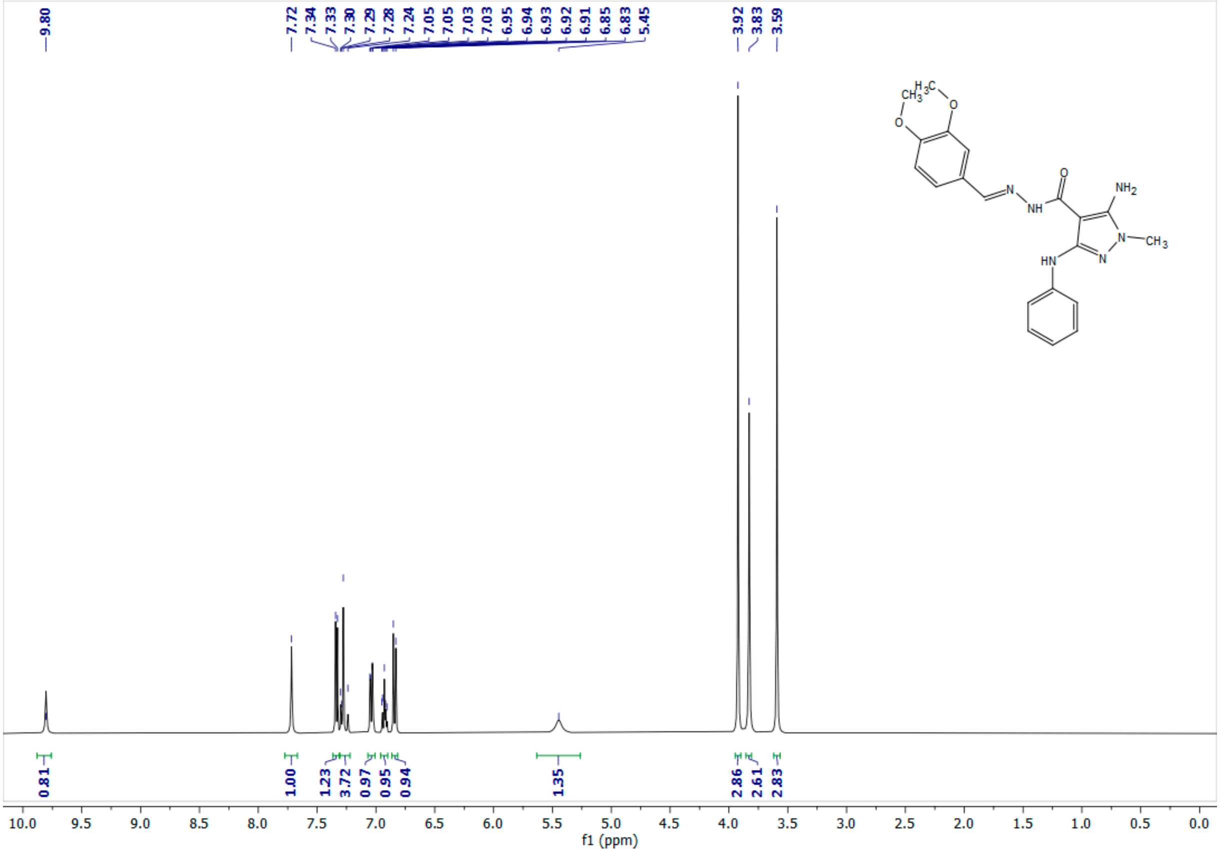

Figure S16. ^{13}C-NMR (101 MHz, d_6-DMSO) spectrum of compound **12b**

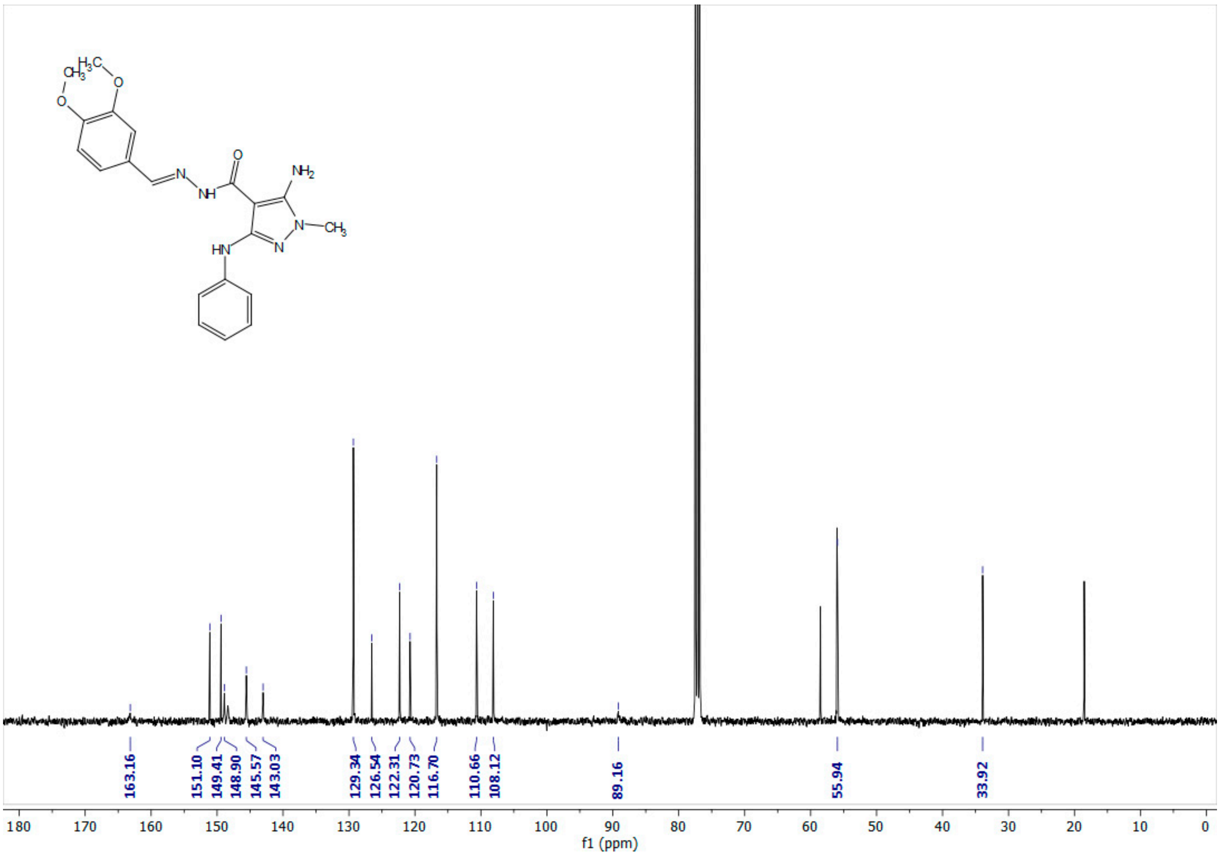

Figure S17. ^{1}H-NMR (400 MHz, d_6-DMSO) spectrum of compound **12c**

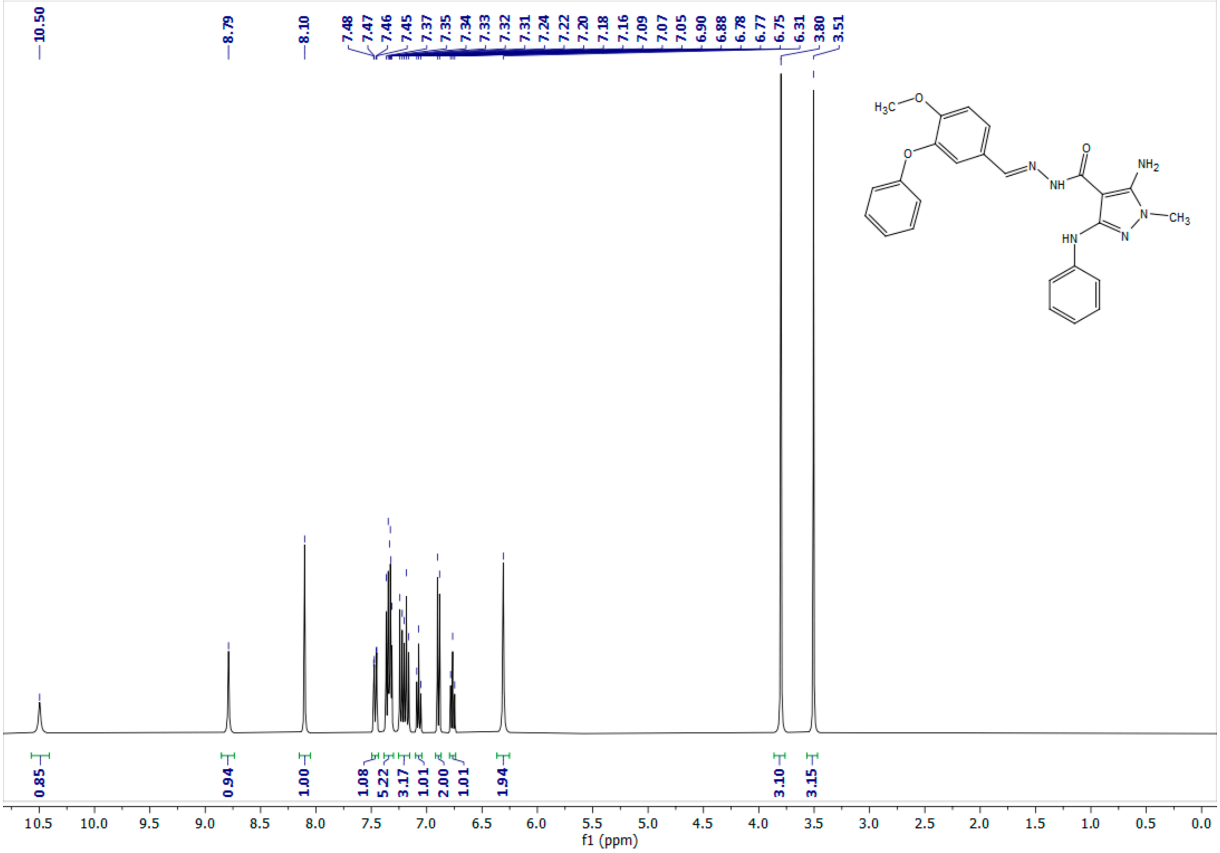

Figure S18. ^{13}C-NMR (101 MHz, d_6-DMSO) spectrum of compound **12c**

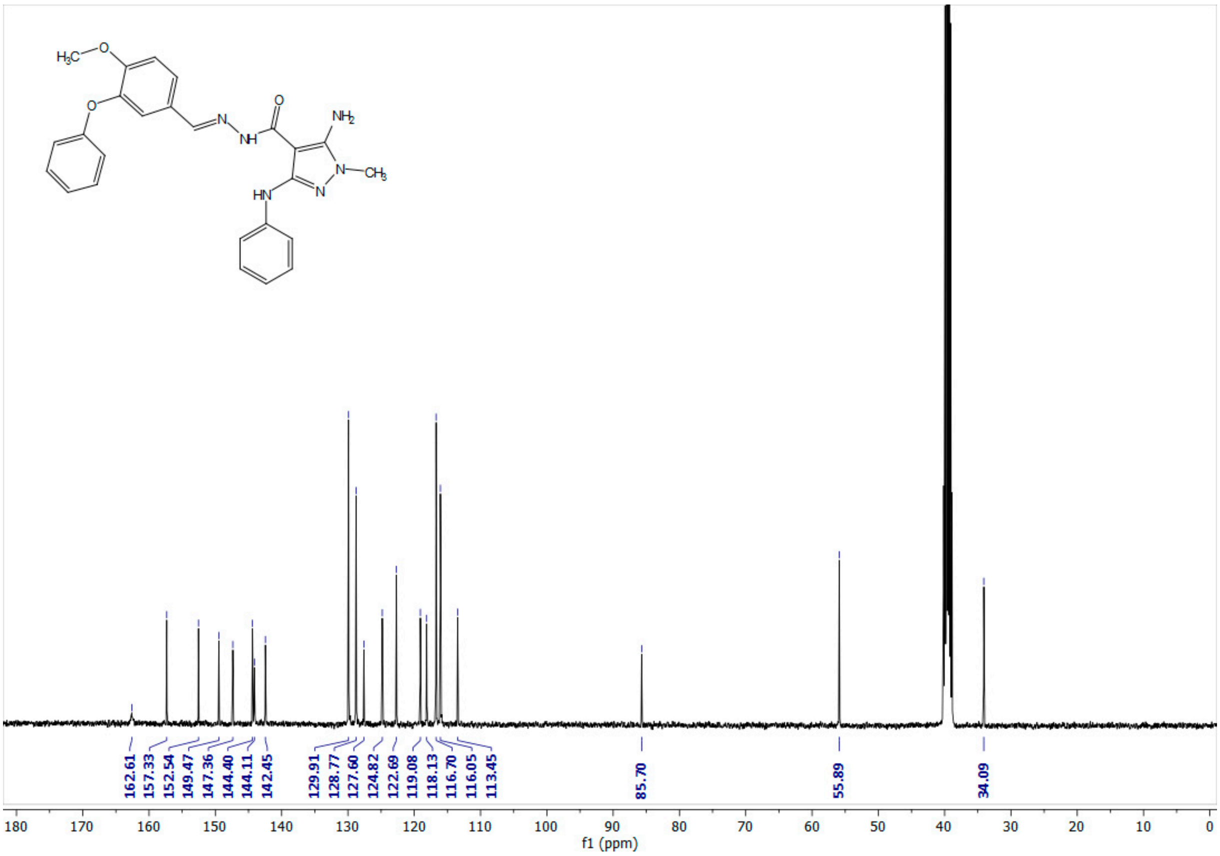

Figure S19. ^{1}H-NMR (400 MHz, d_6-DMSO) spectrum of compound **12d**

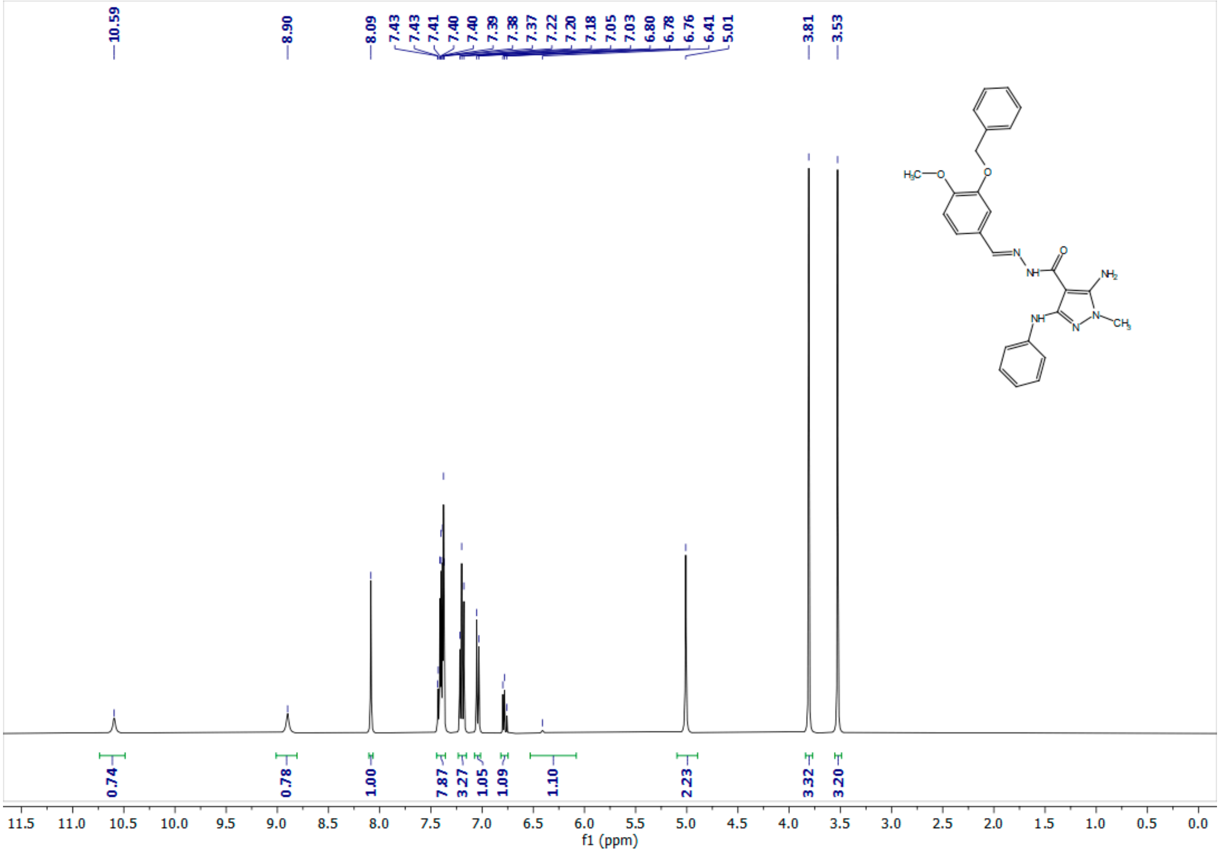

Figure S20. ^{13}C-NMR (101 MHz, d_6-DMSO) spectrum of compound **12d**

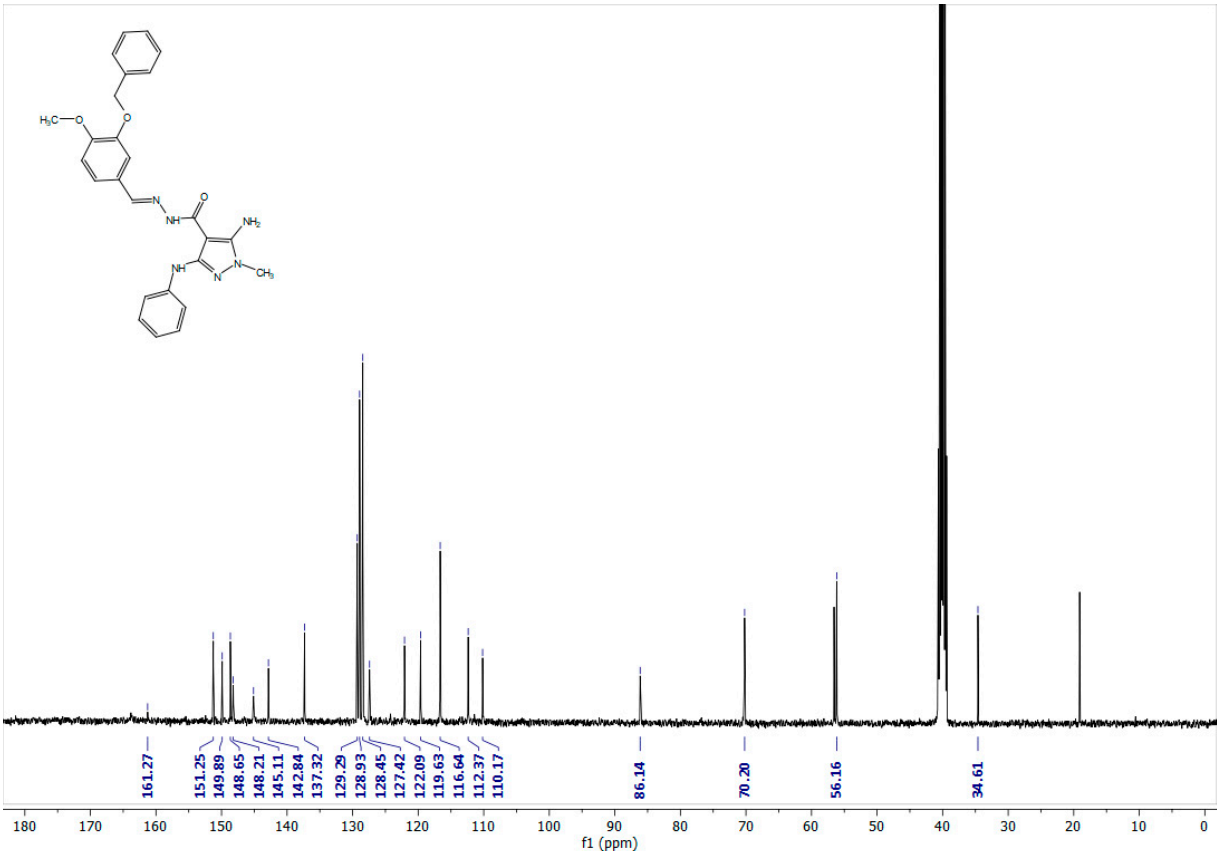

Figure S21. ^{1}H-NMR (400 MHz, d_6-DMSO) spectrum of compound **13a**

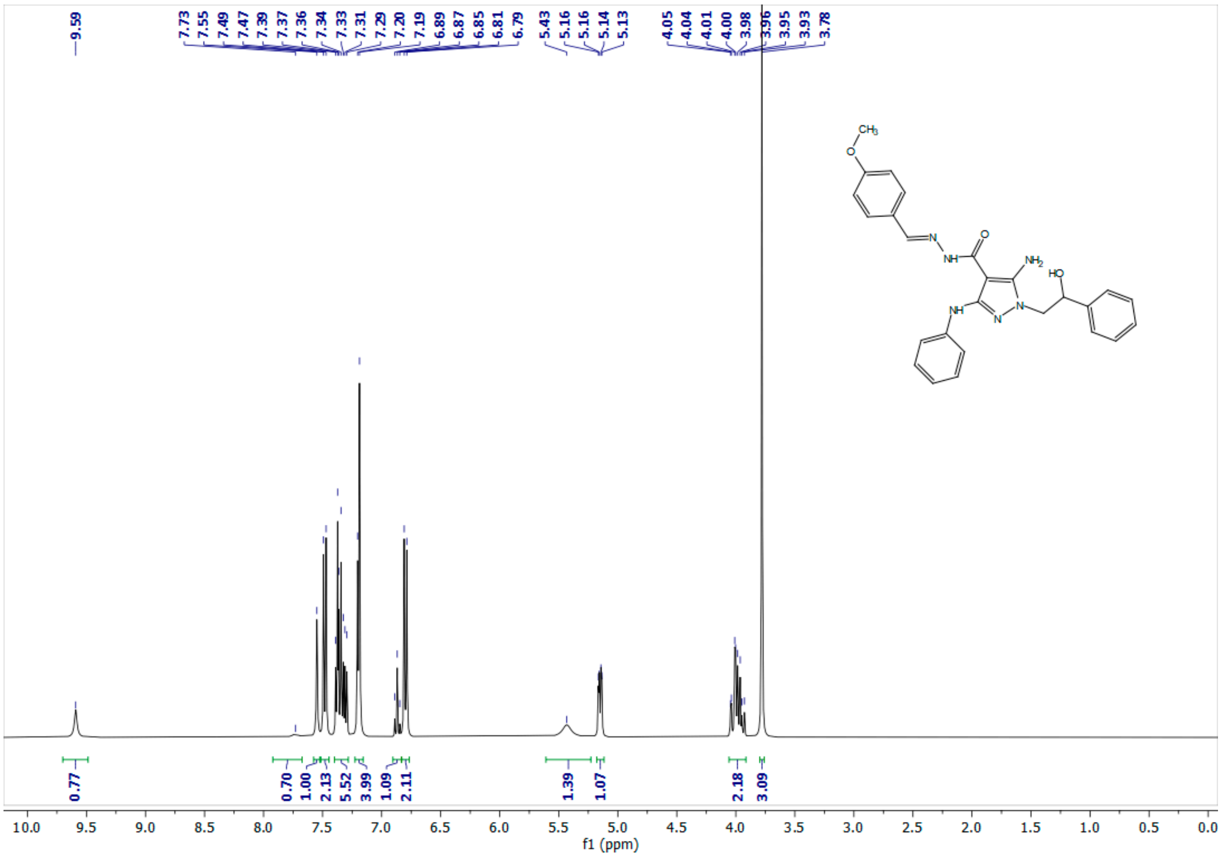

Figure S22. ^{13}C-NMR (101 MHz, d_6-DMSO) spectrum of compound **13a**

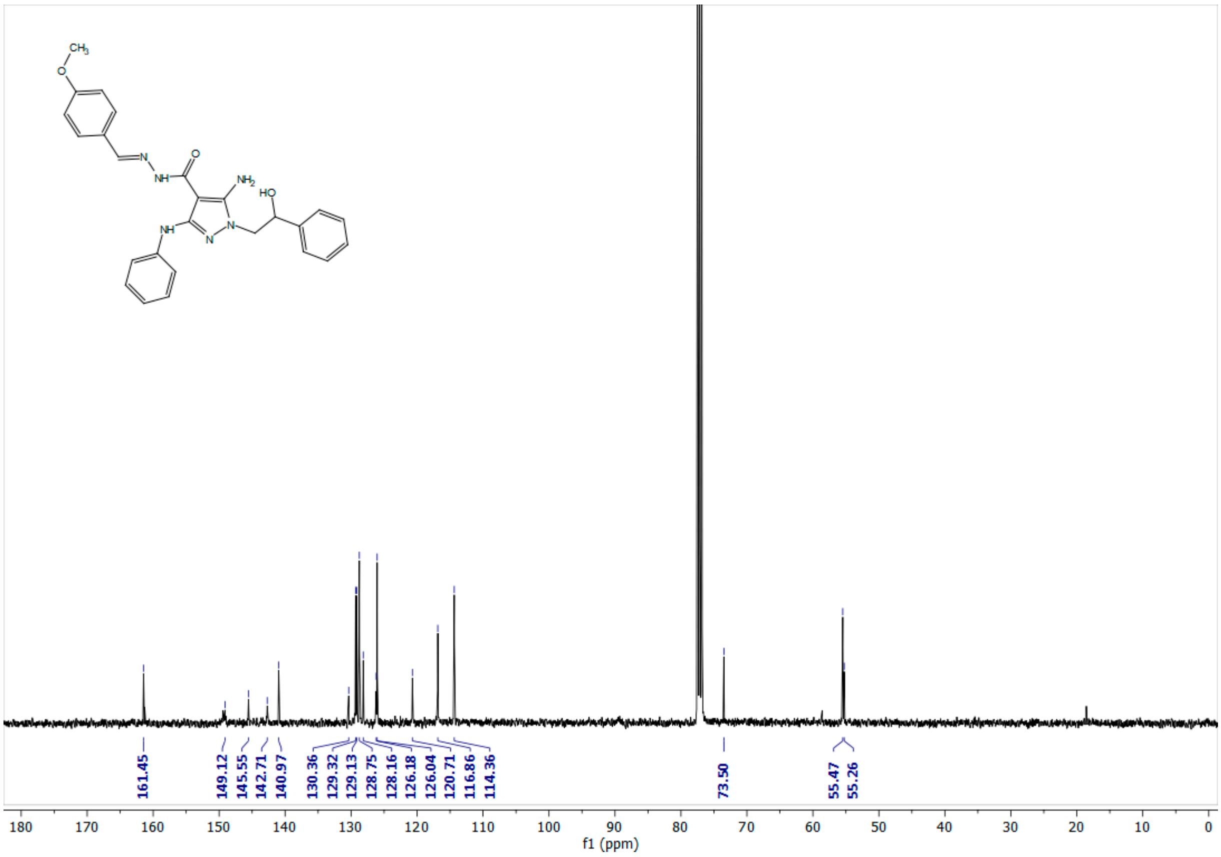

Figure S23. ^{1}H-NMR (400 MHz, d_6-DMSO) spectrum of compound **13b**

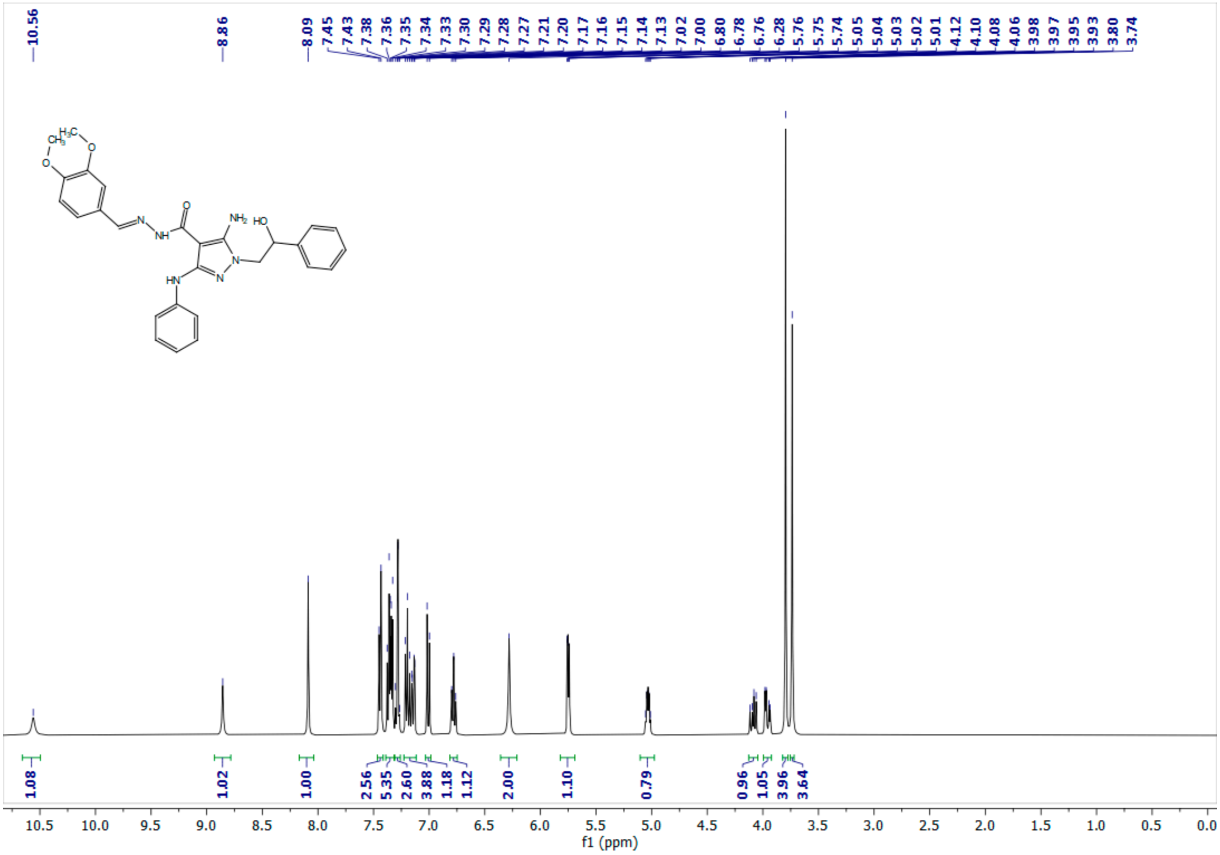

Figure S24. ^{13}C-NMR (101 MHz, d_6-DMSO) spectrum of compound **13b**

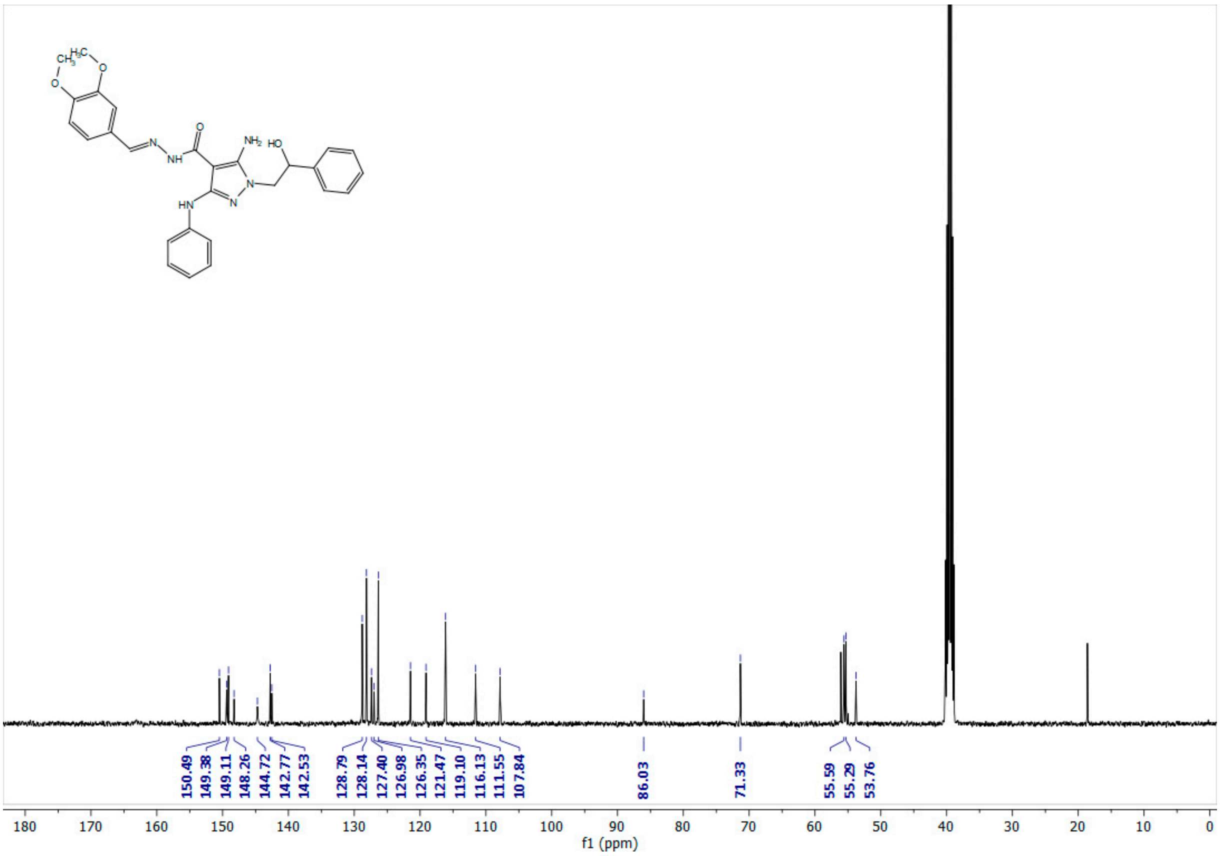

Figure S25. ^{1}H-NMR (400 MHz, d_6-DMSO) spectrum of compound **13c**

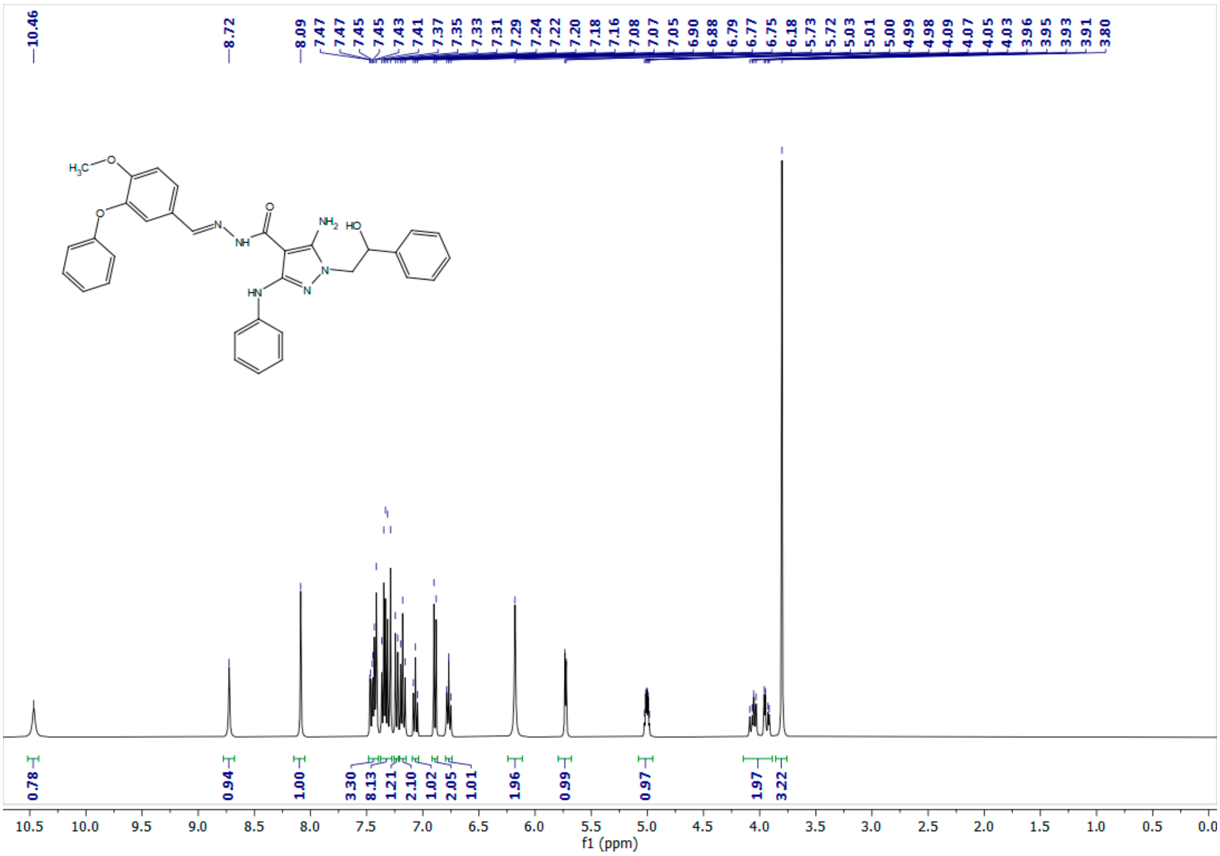

Figure S26. ^{13}C-NMR (101 MHz, d_6-DMSO) spectrum of compound **13c**

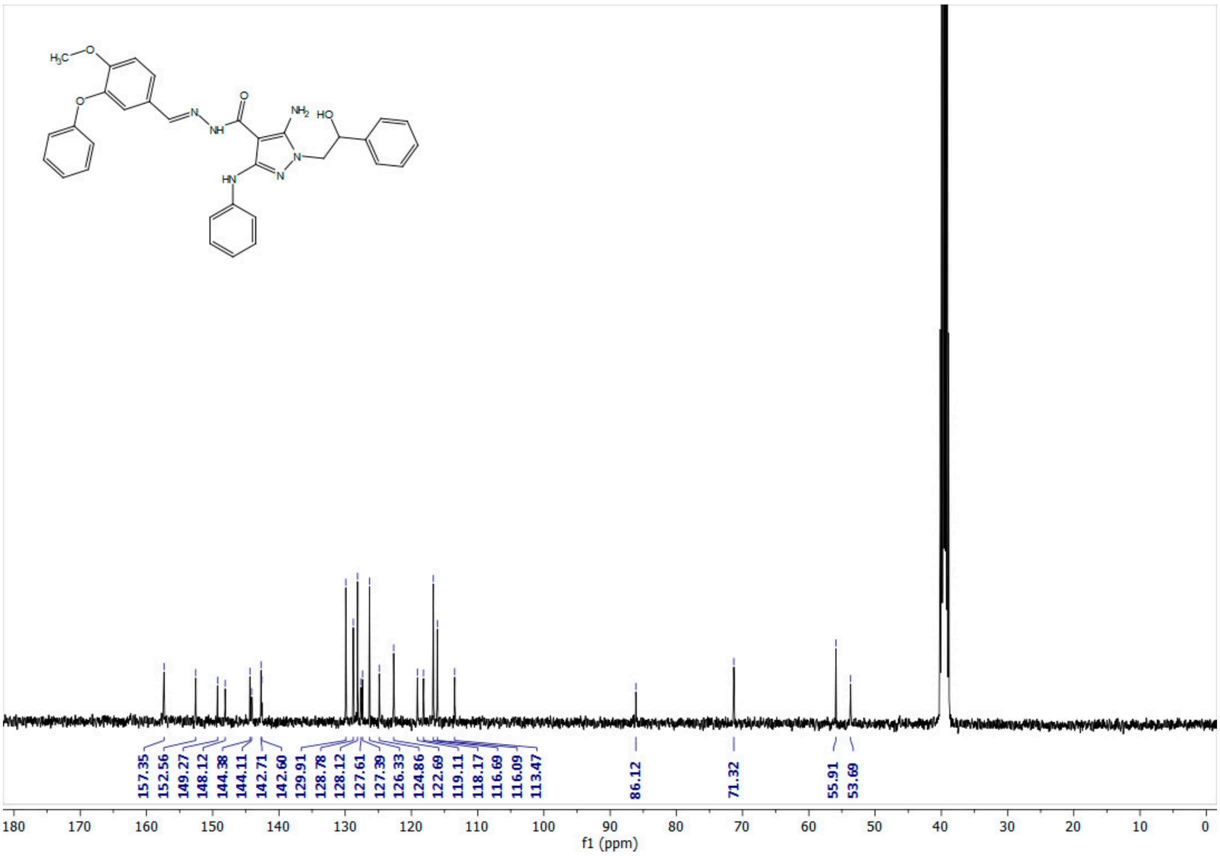

Figure S27. ^{1}H-NMR (400 MHz, d_6-DMSO) spectrum of compound **13d**

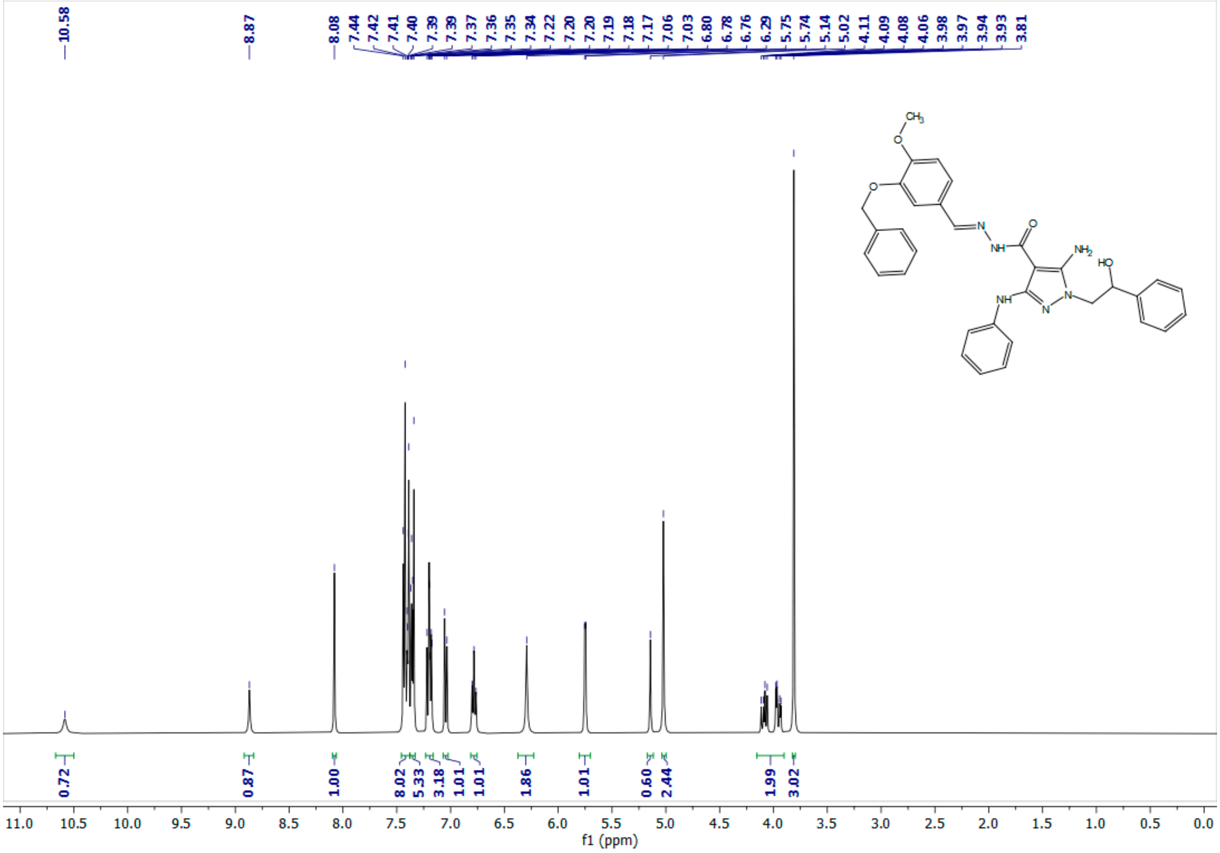

Figure S28. ^{13}C-NMR (101 MHz, d_6-DMSO) spectrum of compound **13d**

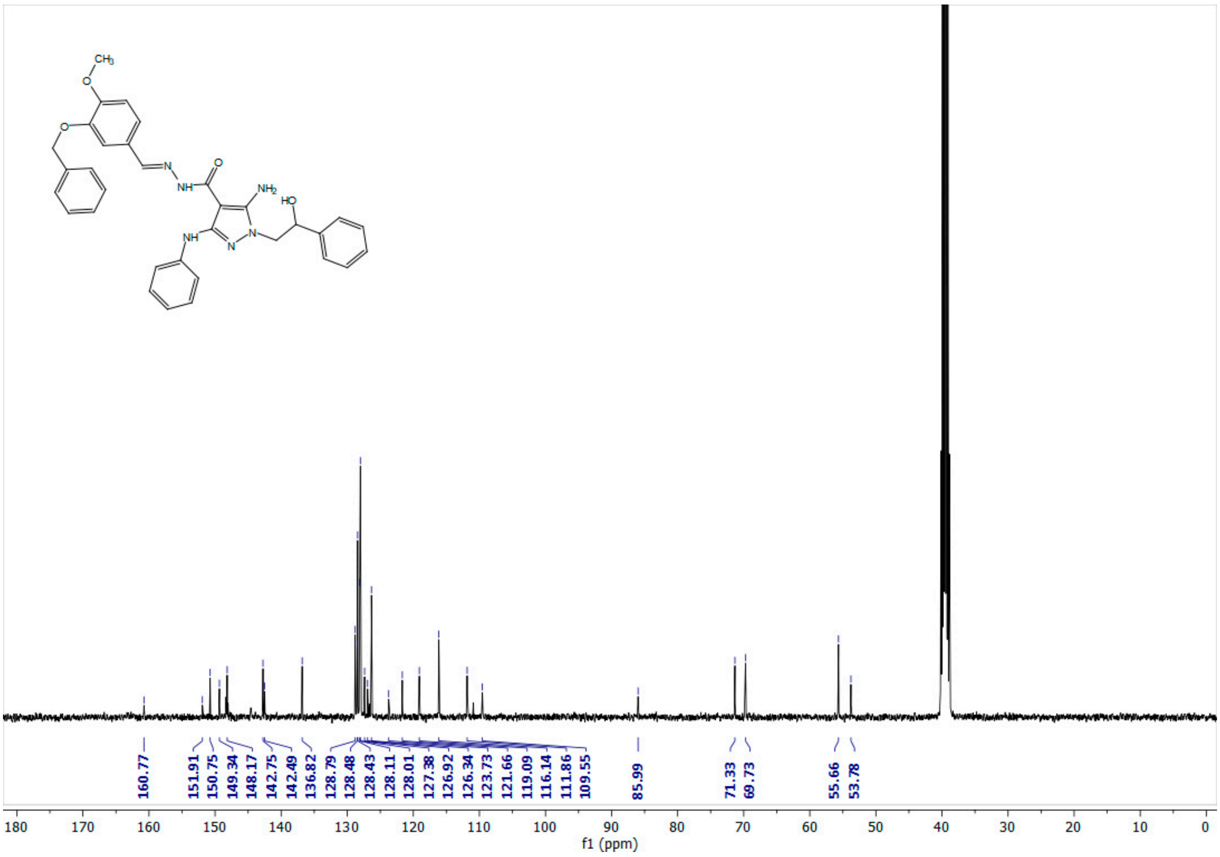

Figure S29. ^{1}H-NMR (400 MHz, d_6-DMSO) spectrum of compound **14**

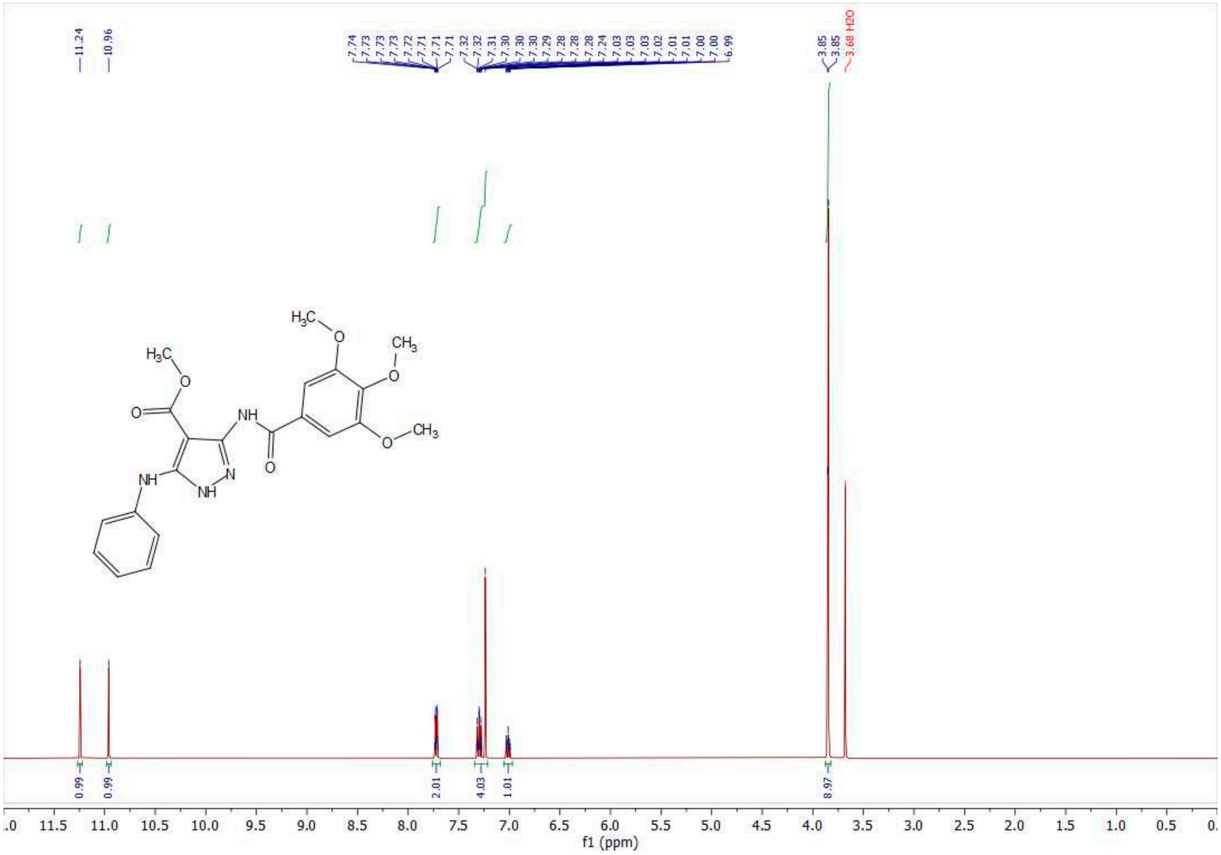

Figure S30. ^{1}H-NMR (400 MHz, d_6-DMSO) spectrum of compound **15**

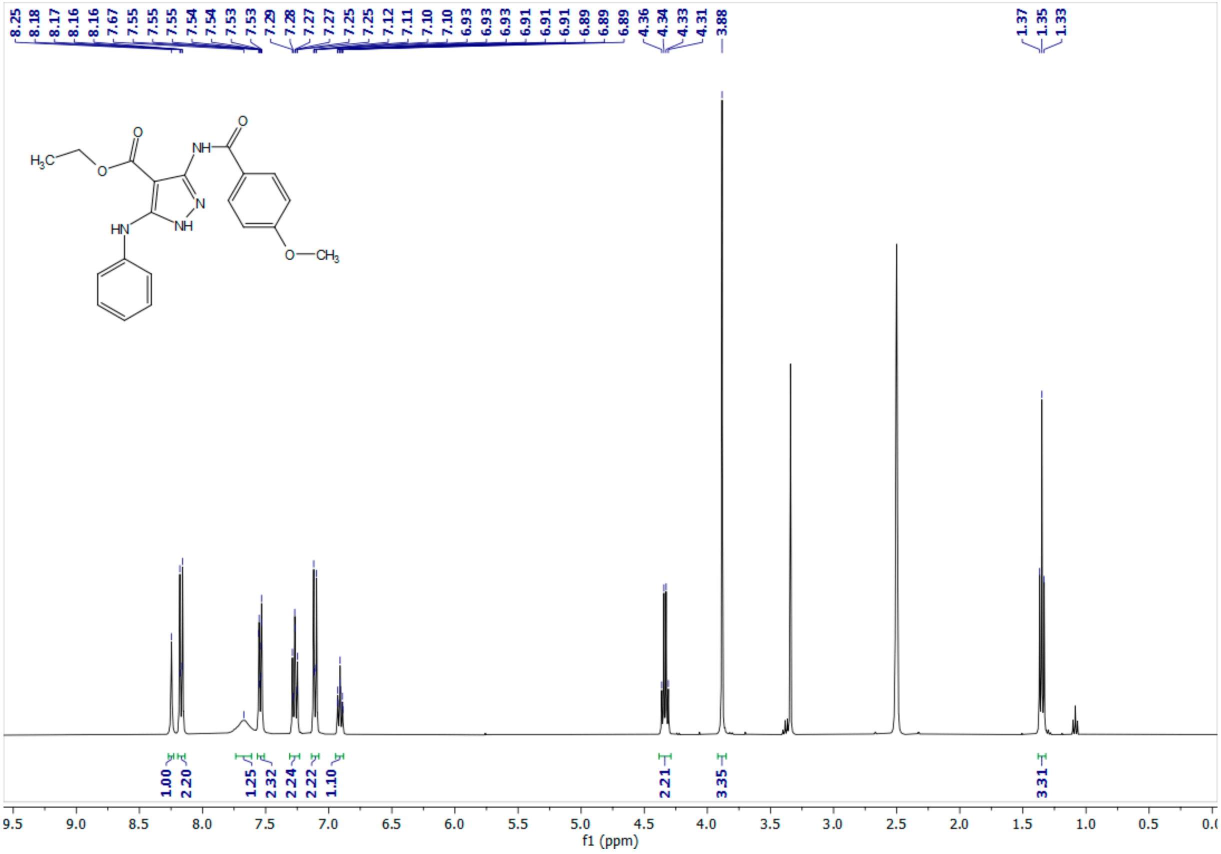

Figure S31. ^{13}C-NMR (101 MHz, d_6-DMSO) spectrum of compound **15**

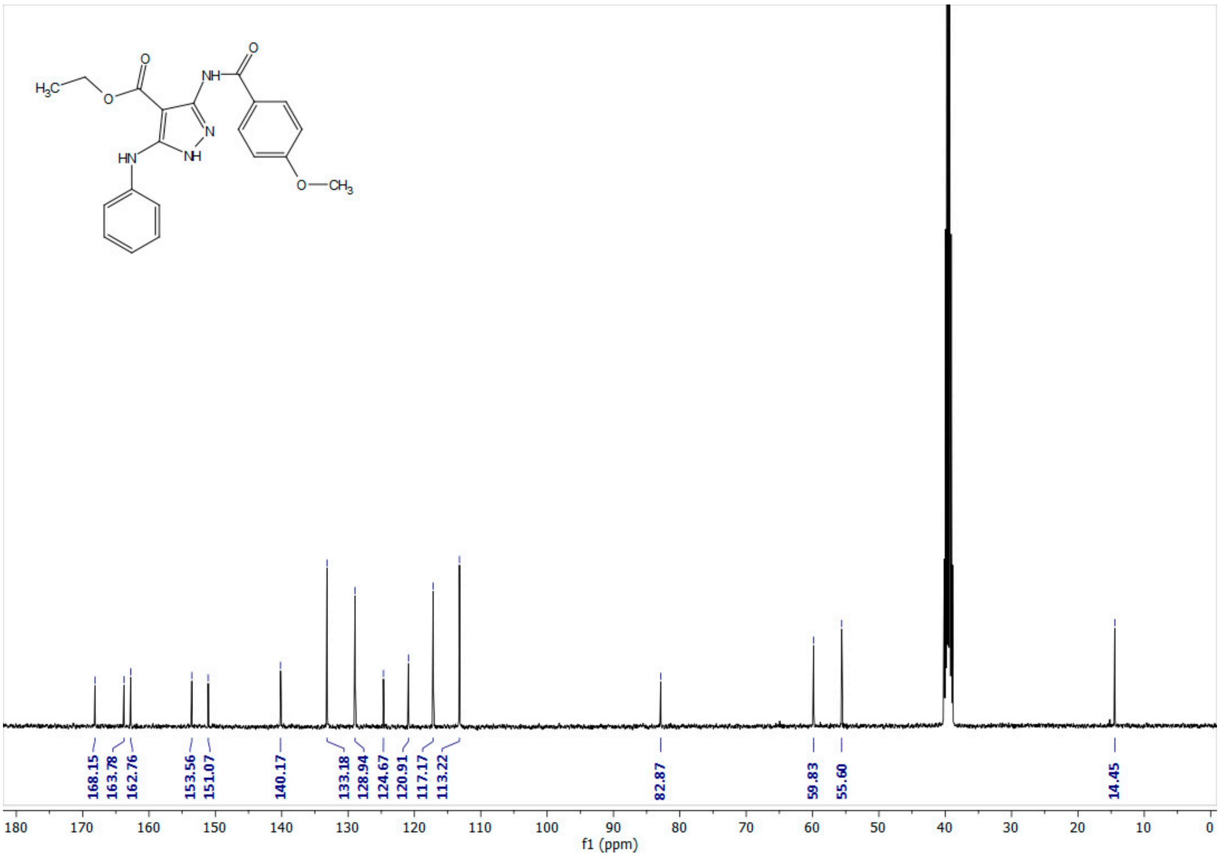

Figure S32. ^{1}H-NMR (400 MHz, d_6-DMSO) spectrum of compound **16**

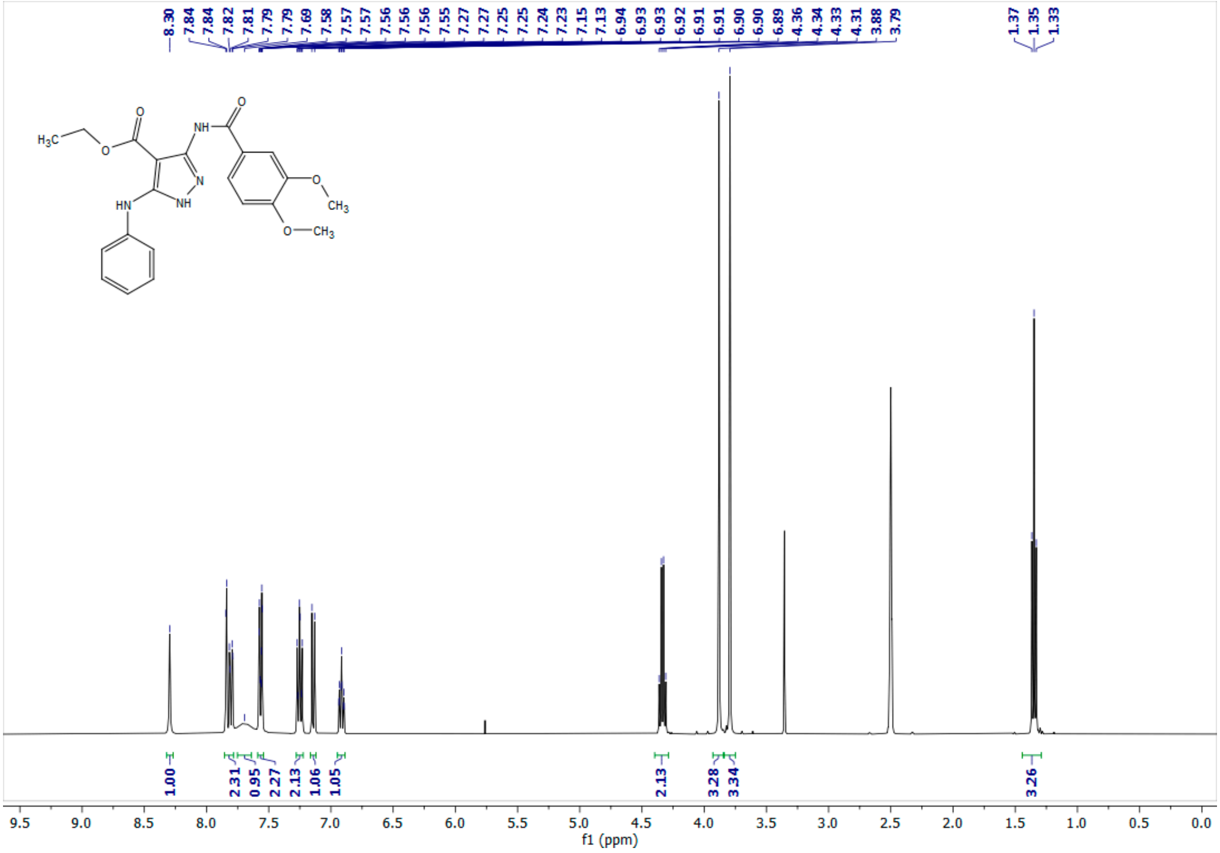

Figure S33. ^{13}C-NMR (101 MHz, d_6-DMSO) spectrum of compound **16**

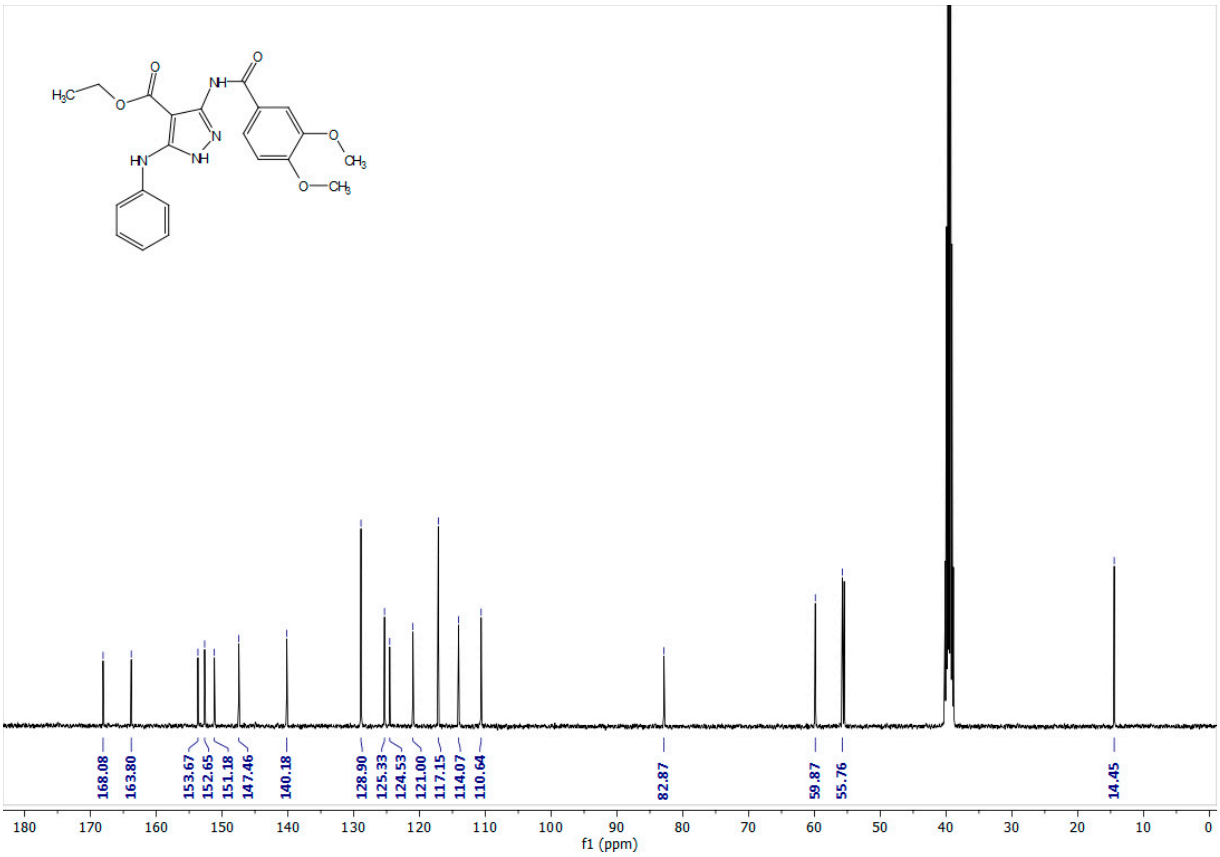

Figure S34. ^{1}H-NMR (400 MHz, d_6-DMSO) spectrum of compound **17**

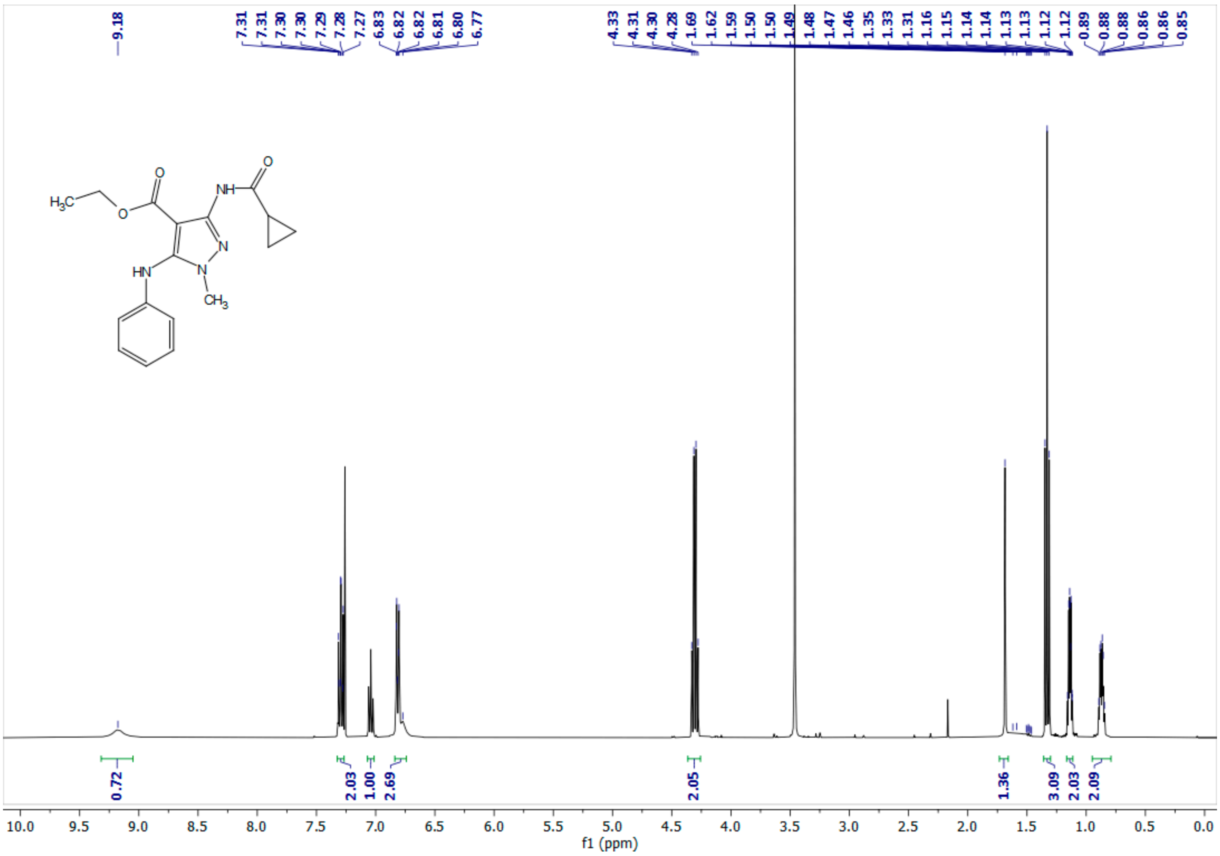

Figure S35. ^{1}H-NMR (400 MHz, d_6-DMSO) spectrum of compound **18**

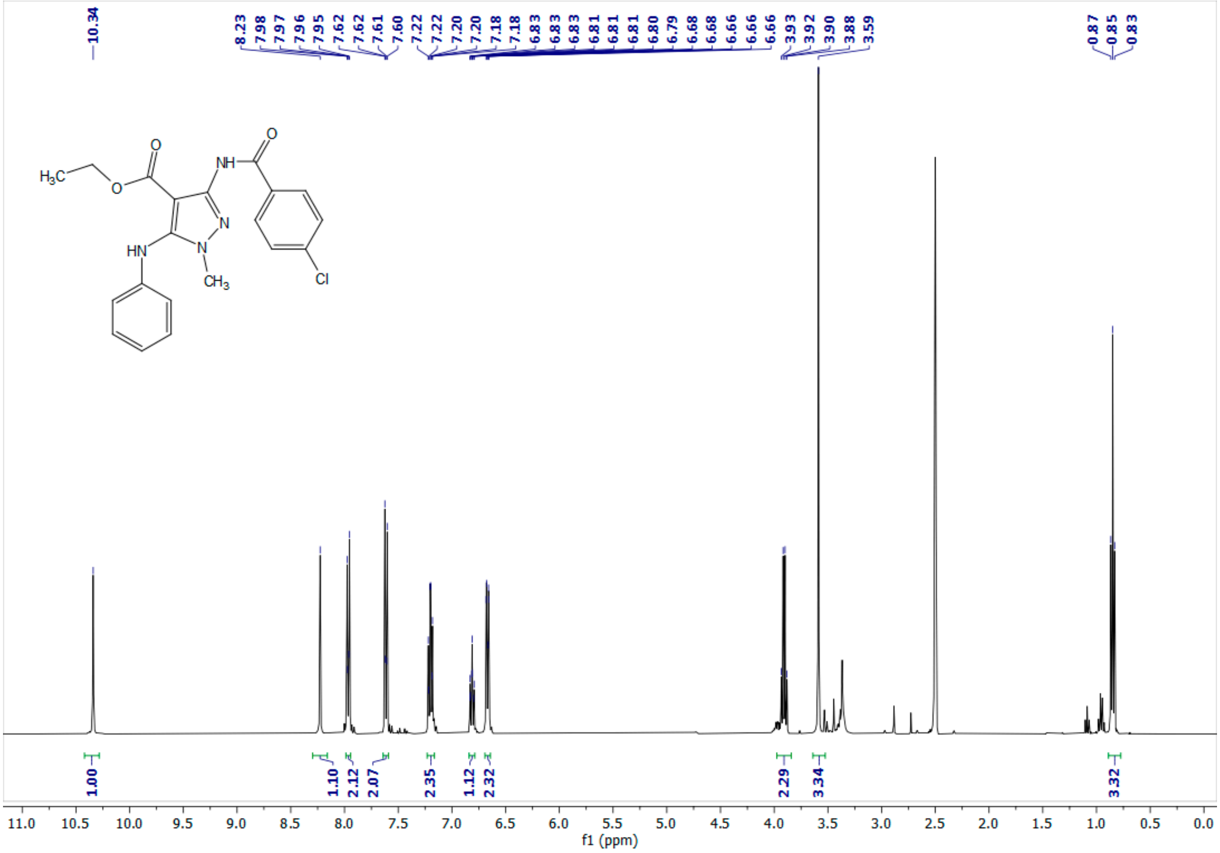

Figure S36. ^{13}C-NMR (101 MHz, d_6-DMSO) spectrum of compound **18**

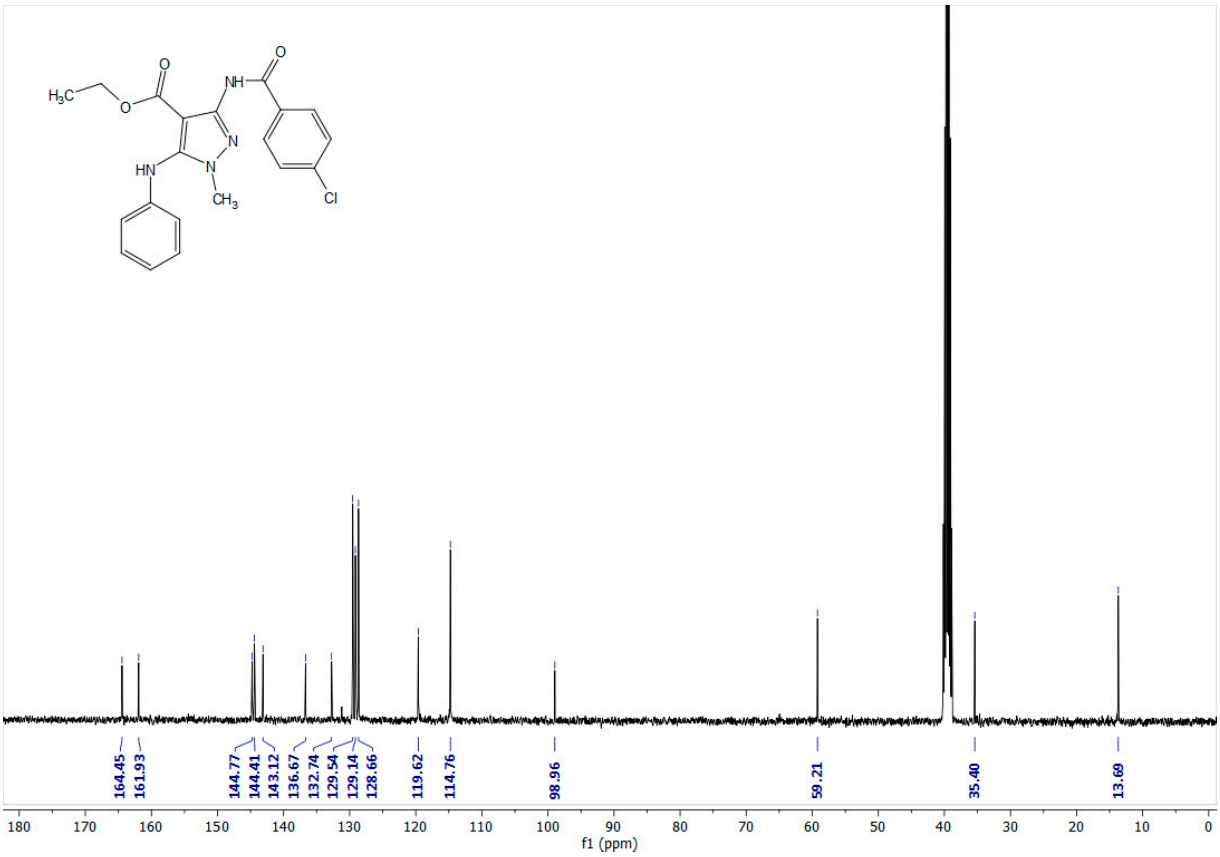

Figure S37. ^{1}H-NMR (400 MHz, d_6-DMSO) spectrum of compound **19**

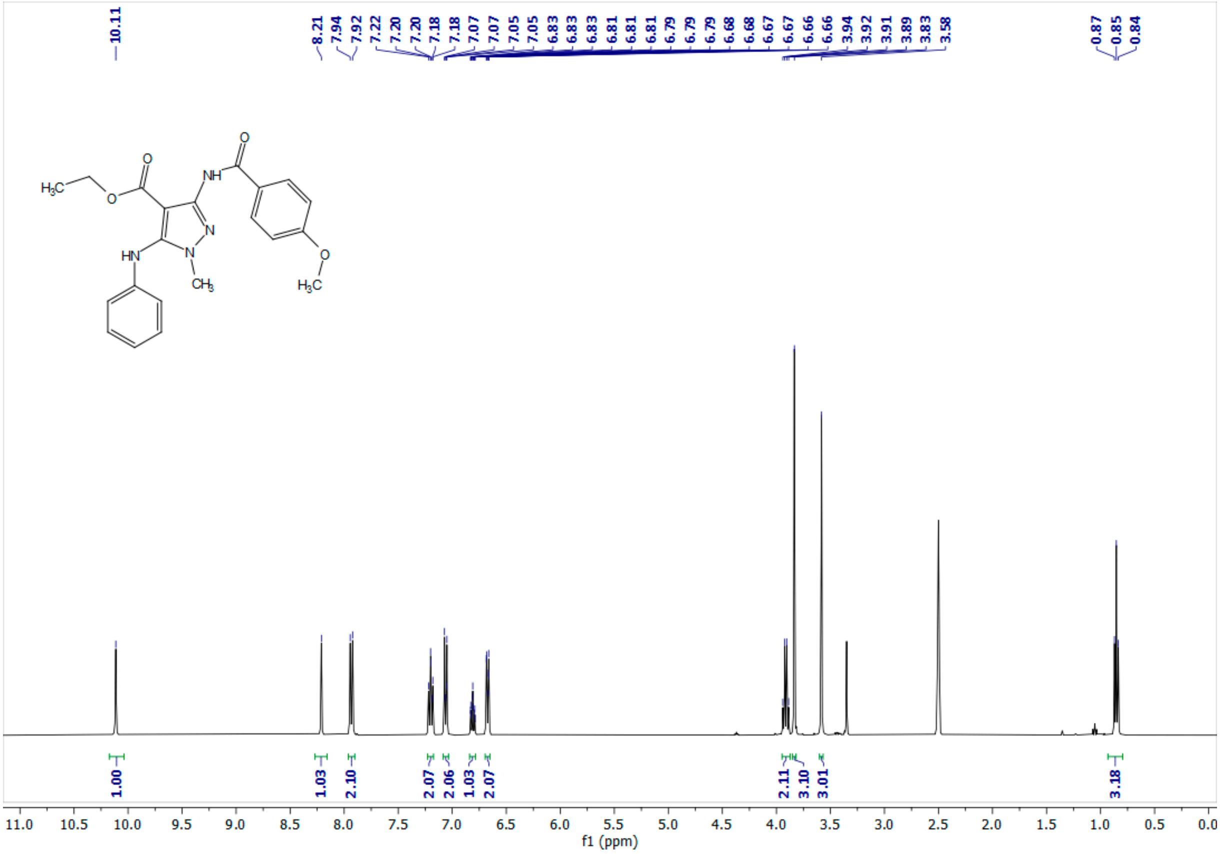

Figure S38. ^{13}C-NMR (101 MHz, d_6-DMSO) spectrum of compound **19**

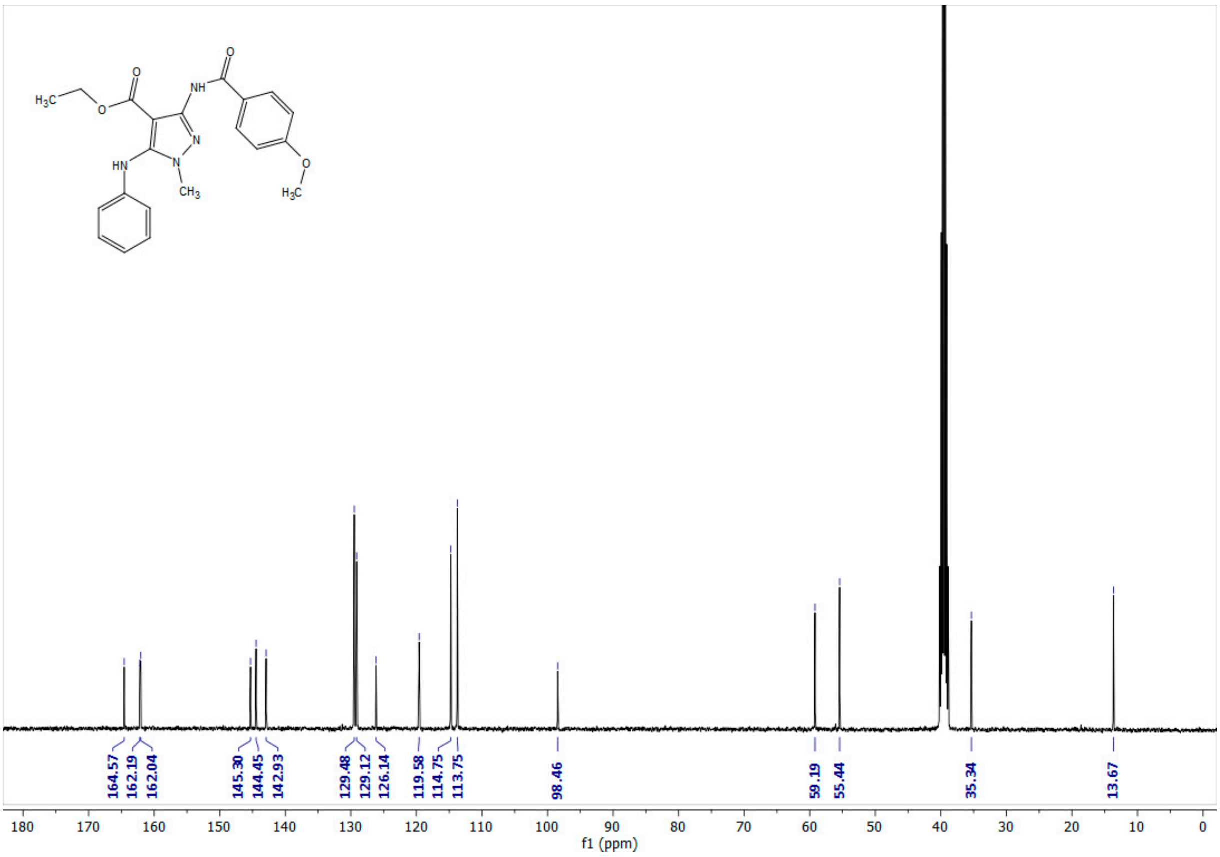

Figure S39. ^{1}H-NMR (400 MHz, d_6-DMSO) spectrum of compound **20**

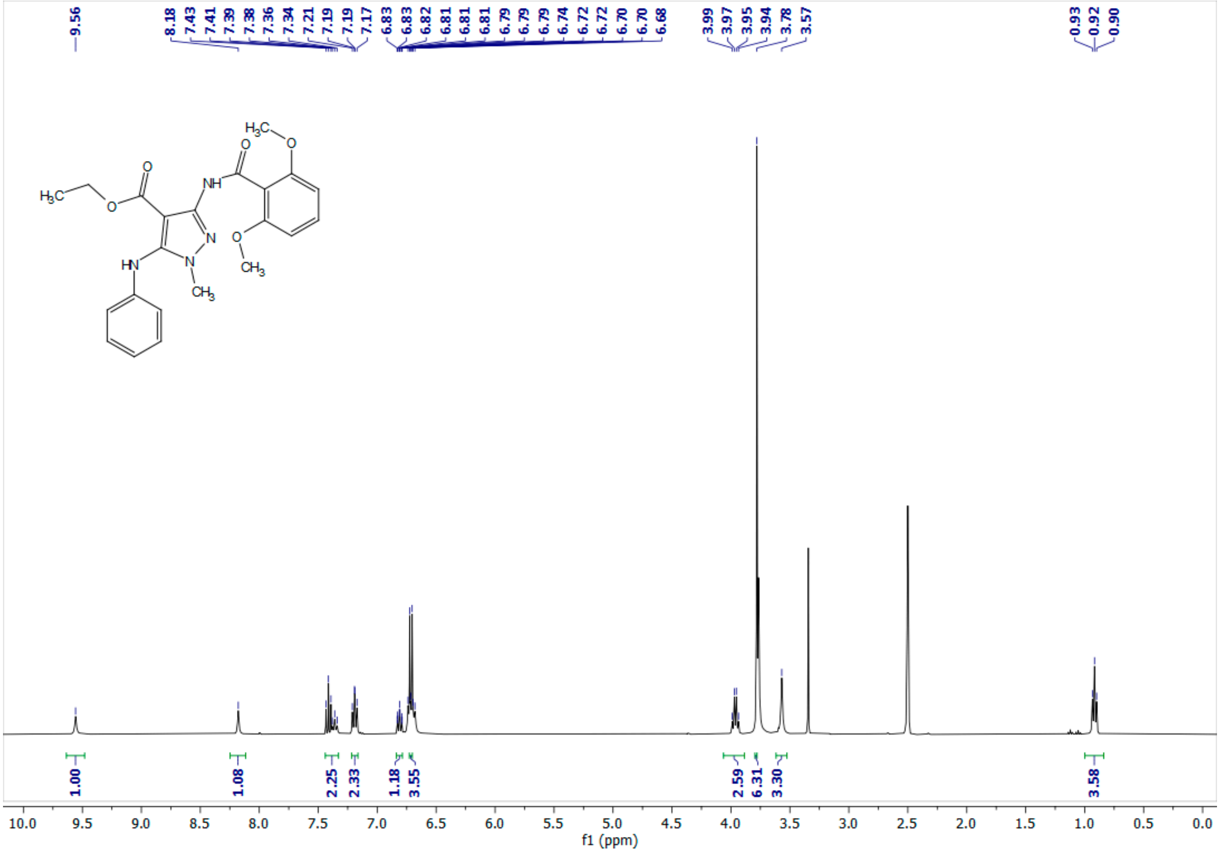

Figure S40. ^{13}C-NMR (101 MHz, d_6-DMSO) spectrum of compound **20**

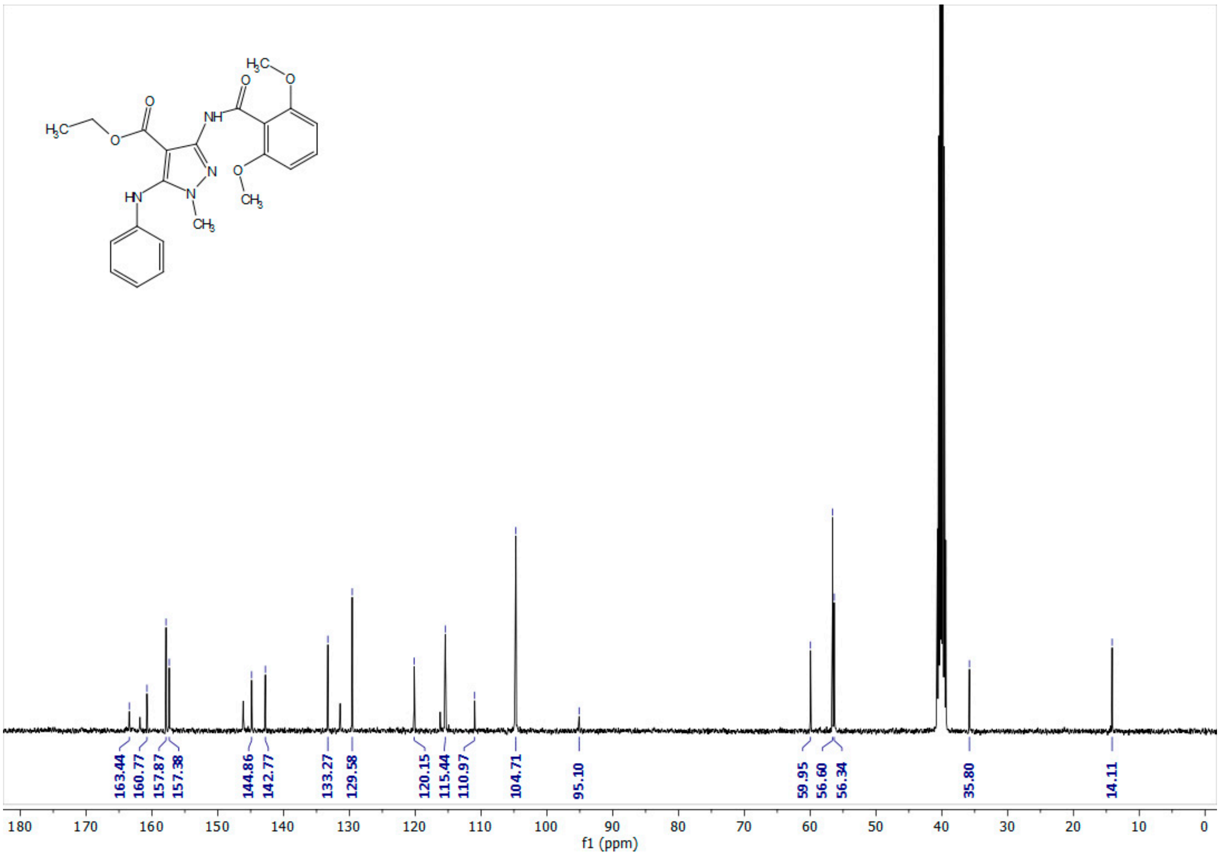

Figure S41. ^{1}H-NMR (400 MHz, d_6-DMSO) spectrum of compound **21**

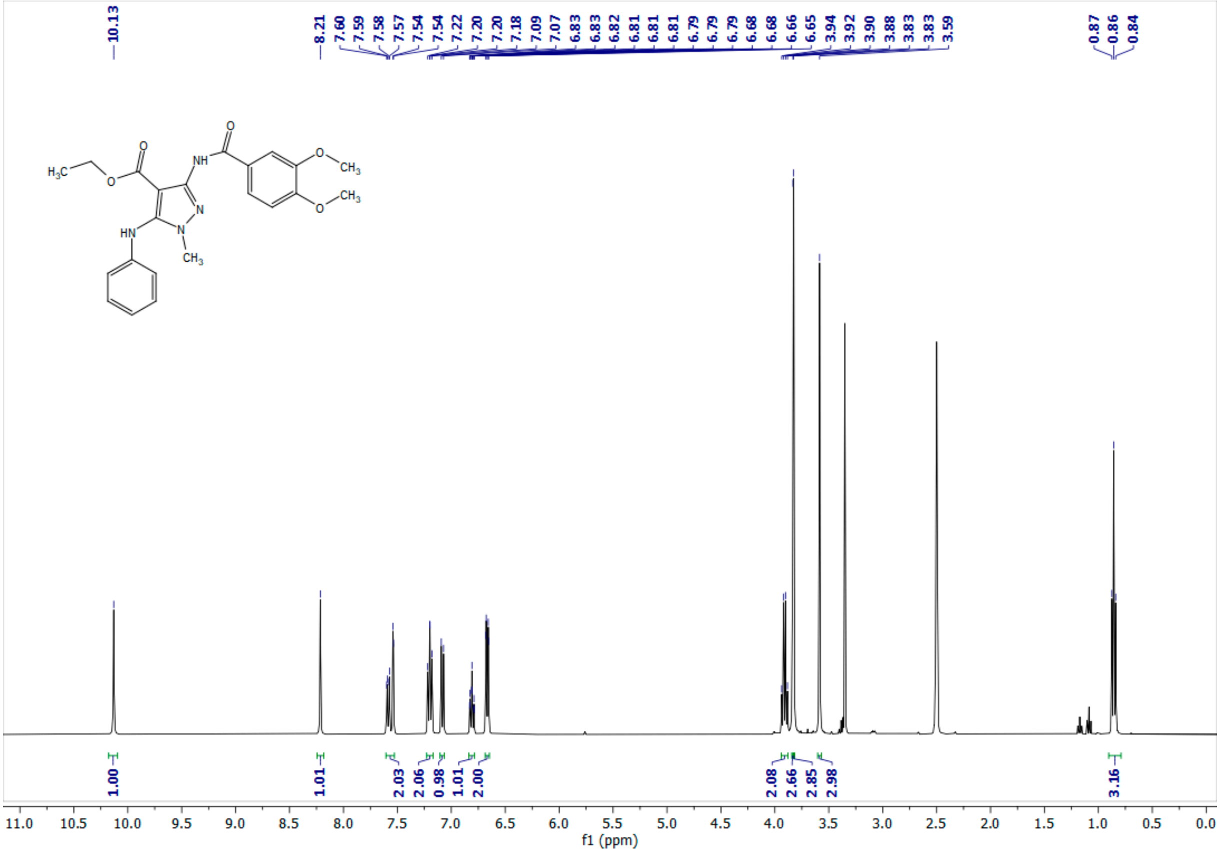

Figure S42. ^{13}C-NMR (101 MHz, d_6-DMSO) spectrum of compound **21**

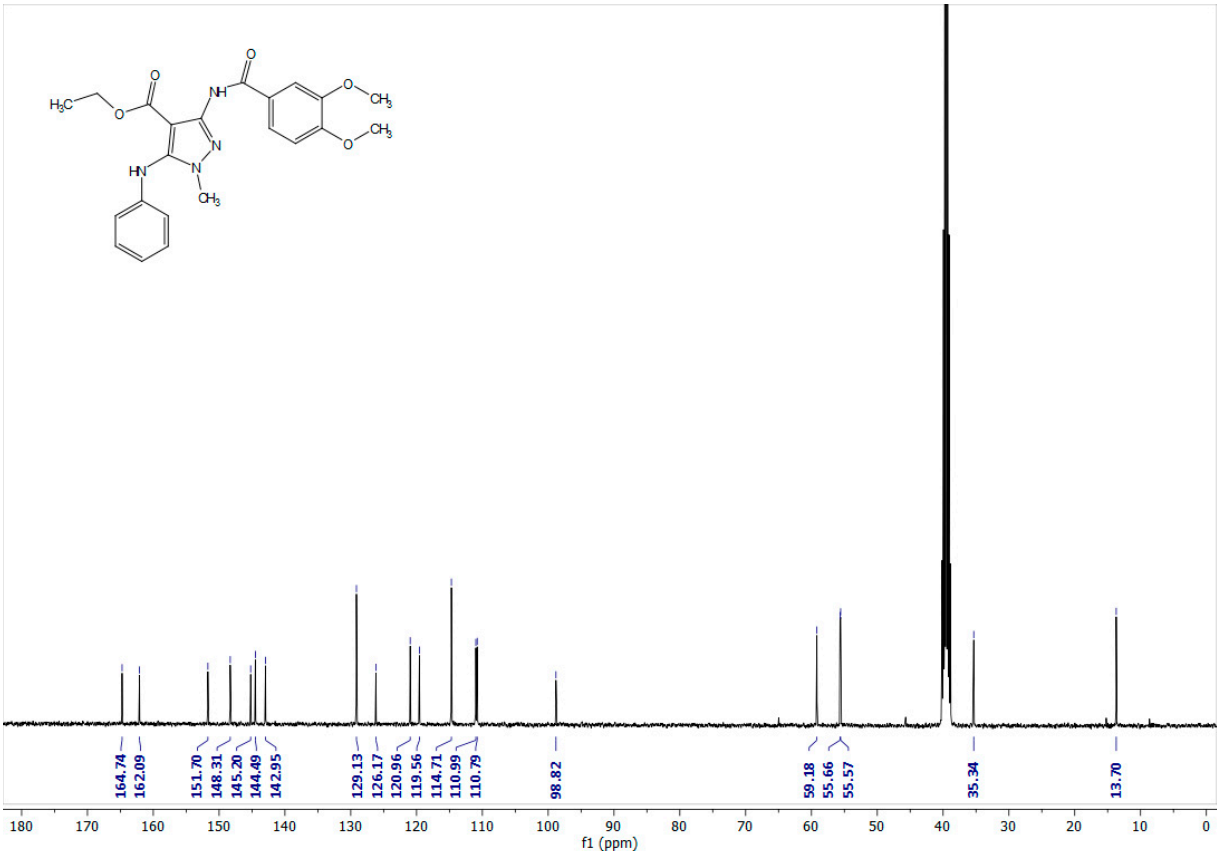

Figure S43. ^{1}H-NMR (400 MHz, d_6-DMSO) spectrum of compound **22**

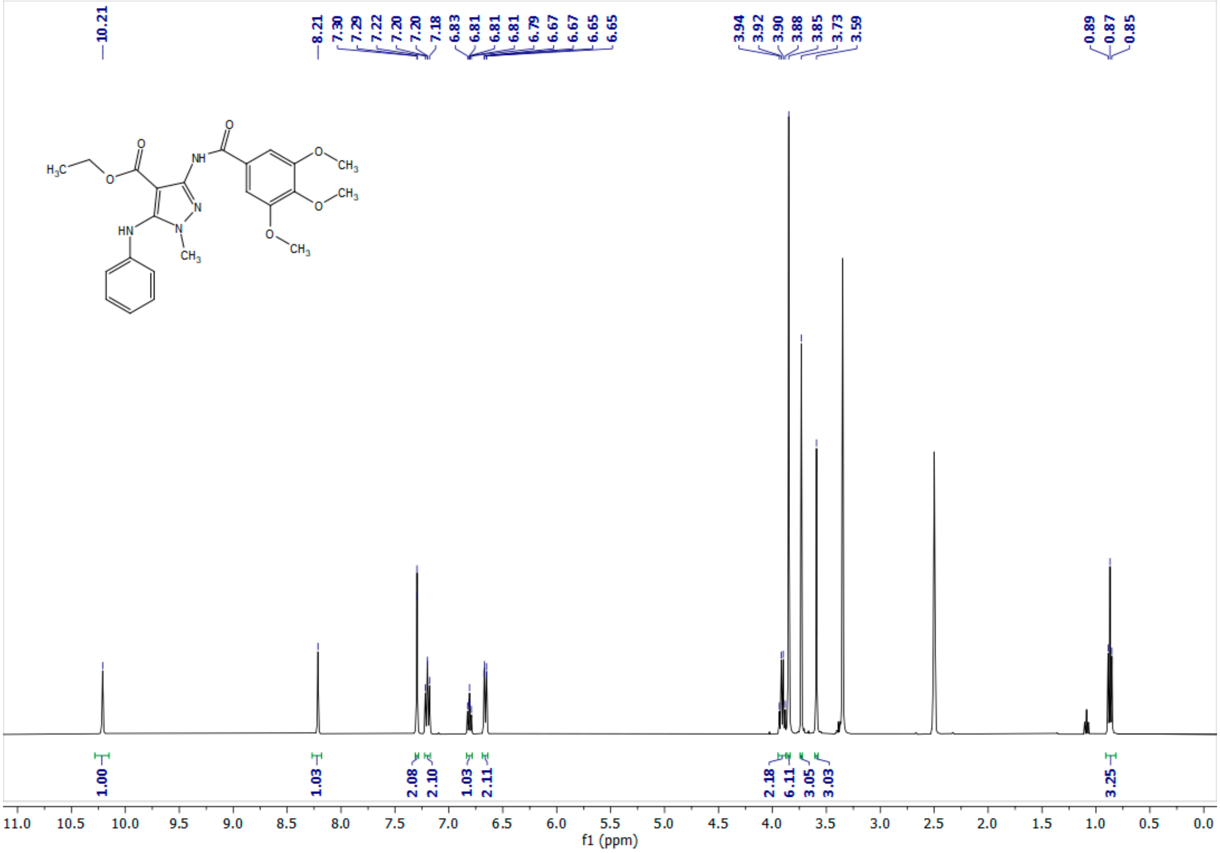

Figure S44. ^{13}C-NMR (101 MHz, d_6-DMSO) spectrum of compound **22**

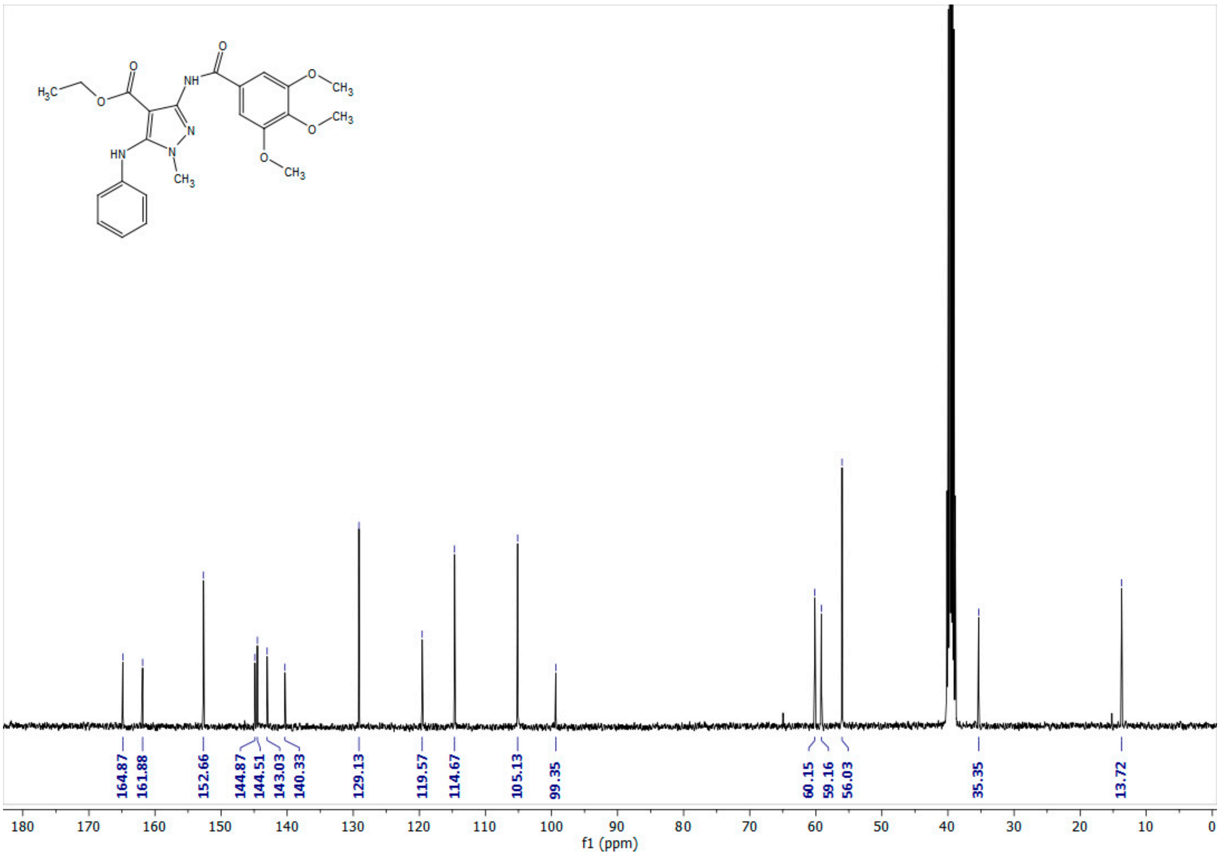

Table S1. Inhibitory effect of compounds **10-22** on platelet aggregation and ROS production

	**IC_{50} (μM±SD)[a]**	
**Cpd**	**Aggregation inhibition**	**ROS production inhibition**
**10a**	204±12	111±9
**10b**	94±7	104±4
**11a**	183±13	123±12
**11b**	129±10	113±7
**11c**	841±43	148±13
**11d**	890±19	182±11
**12a**	265±23	113±7
**12b**	187±16	106±9
**12c**	331±14	193±16
**12d**	433±16	171±9
**13a**	883±39	213±17
**13b**	909±87	433±32
**13c**	313±29	209±15
**13d**	437±25	405±29
**14**	365±28	387±35
**15**	250±19	323±14
**16**	292±28	310±24
**17**	460±35	573±43
**18**	268±25	273±17
**19**	294±26	293±18
**20**	301±24	289±27
**21**	278±27	262±13
**22**	249±22	313±25
**NAC**	ND	872±26
**ASA**	438±18	ND

[a]Reported data are the mean value ± standard deviation (SD) obtained in at least six different experiments each performed in duplicate.
